# Phytochemical Profiling, Antioxidant Activity, and *In Silico* Analyses of *Sterculia villosa* and *Vernonia patula*

**DOI:** 10.1155/2022/3190496

**Published:** 2022-06-06

**Authors:** Chadni Lyzu, Saikat Mitra, Kahkashan Perveen, Zidan Khan, Abu Montakim Tareq, Najat A. Bukhari, Fohad Mabood Husain, Evena Parvin Lipy, Dipa Islam, Mahmuda Hakim, Talha Bin Emran, Marjan Ganjali Dashti

**Affiliations:** ^1^Biomedical and Toxicological Research Institute, Bangladesh Council of Scientific and Industrial Research (BCSIR), Dr. Qudrat-I-Khuda Road, Dhanmondi, Dhaka 1205, Bangladesh; ^2^Department of Pharmacy, Faculty of Pharmacy, University of Dhaka, Dhaka 1000, Bangladesh; ^3^Department of Botany & Microbiology, College of Science, King Saud University, Riyadh-11495, Saudi Arabia; ^4^Department of Pharmacy, International Islamic University Chittagong, Chittagong 4318, Bangladesh; ^5^Department o Food Science and Nutrition, College of Food and Agriculture, King Saud University, Riyadh 11421, Saudi Arabia; ^6^Department of Pharmacy, BGC Trust University Bangladesh, Chittagong 4381, Bangladesh; ^7^Department of Pharmacy, Faculty of Allied Health Sciences, Daffodil International University, Dhaka 1207, Bangladesh; ^8^Department of Biological Sciences, University of Texas at Dallas, 800 W Campbell Road, Richardson 75080, TX, USA

## Abstract

Our study aims to evaluate the chemical profiles and antioxidant activities of a methanolic extract of *Sterculia villosa* bark (MESV) and a methanolic extract of the *Vernonia patula* whole plant (MEVP). The chemical profiling of MESV and MEVP was performed via gas chromatography-mass spectrometry (GC-MS), which identified 52 and 33 chemical compounds, respectively. The 2,2-diphenyl-1-picrylhydrazyl (DPPH) assay indicated that both MESV and MEVP displayed concentration-dependent scavenging activities, and half-maximal inhibitory concentration (IC_50_) values for MEVP, MESV, and ascorbic acid were 305.30, 555.44, and 36.32 *μ*g/mL, respectively. The total flavonoid content (TFC) and total phenolic content (TPC) of MESV were 81.44 ± 2.70 mg quercetin equivalents (QE)/g dry extract and 62.58 ± 1.93 mg gallic acid equivalent (GAE)/g dry extract, whereas these values for MEVP were 291.31 ± 6.61 mg QE/g dry extract and 58.99 ± 3.16 mg GAE/g dry extract, respectively. Molecular docking studies were also evaluated, and absorption, distribution, metabolism, and excretion (ADME) and toxicological properties were assessed. Therefore, these two plants, *S. villosa* and *V. patula*, showed potential options for further advanced studies into oxidative stress.

## 1. Introduction

Plants containing natural bioactive compounds have been used in traditional medicinal practices worldwide since ancient times, and plants represent a source of potential medicines [1]. The scope of plants as a source of new drugs remains generally unexplored, as only a small fraction of approximately 250,000–500,000 plant species have been biologically or pharmacologically screened [2]. Phytochemicals with antioxidant properties are of particular interest because chronic disorders [3] exacerbated by oxidative stress (OS) have become the leading cause of death [4]. Plant-derived compounds possess potent antioxidant properties that may inhibit OS by counteracting reactive oxygen species (ROS) and maintaining redox homeostasis [5, 6]. Several attempts have been undertaken to identify phytochemical compounds [4, 7] and assess their potential as antioxidants [8, 9], antimicrobials [10], antidiabetics, and anti-inflammatory compounds [11, 12]. The therapeutic potential of plants is commonly associated with their antioxidant and anticancer properties [2, 13–16].

Oxidation refers to the removal of electrons during a reaction by an atom, molecule, or ion and can occur following the formation of elevated amounts of ROS. ROS are formed during natural cellular metabolic processes by living organisms, including byproducts of aerobic metabolism. ROS include hydrogen peroxide (H_2_O_2_), superoxide anion (O_2_−), and hydroxyl radicals (OH•), which all have inherent chemical properties and provide reactivity to various biological objectives [17]. ROS are also correlated with the concept of OS, and ROS can damage lipids, proteins, and DNA [18]. OS is a complicated process involving the generation of ROS and reactive nitrogen species (RNS) [19, 20]. ROS are produced by persistent metabolic processes and can regulate various biological and pathological processes, such as lipid peroxidation, immune response, and phagocyte activation [21]. Furthermore, excessive ROS generation can trigger oxidative damage by targeting the unsaturated fatty acids in membranes and thiol groups in proteins [22, 23]. Many chronic health problems have been associated with excessive lipid peroxidation, and free radicals have been implicated in the induction of several neuropsychiatric conditions and might mediate neuronal malfunctions associated with depression [24, 25]. OS has been identified as a significant contributor to the progression of degenerative and chronic diseases, such as malignant growths [26], diabetes, immune problems, joint inflammation, and cardiovascular and neurodegenerative diseases [27].

*Sterculia villosa* (Family: Sterculiaceae, Bengali name: Udal) is a deciduous tree with large, long-stalked, deeply lobed leaves and yellow flowers. *S. villosa* can be found in subtropical and tropical regions, including Bangladesh [7]. The plant is traditionally used as a diuretic and aphrodisiac agent [28] and is often used by Indian people to cure inflammation through traditional medicinal practices [29]. *Vernonia patula* (Family: Asteraceae, Bengali name: Kukshim) is an annual weed that is geographically disseminated throughout Bangladesh and is known as purple fleabane. This plant has significant medicinal value, used for fever reduction, headaches, malaria, common cold, and intestinal and stomach problems [30]. The bioactive compounds derived from these two plants have been reported to have antioxidant activities in prior studies [31, 32]. However, prior studies did not attempt to identify specific bioactive compounds.

Therefore, the present research attempted to identify the bioactive constituents of these two species through gas chromatography-mass spectrometry (GC-MS) analysis and explored the antioxidant efficacy of the compounds found in *S. villosa* and *V. patula*. GC-MS has been widely highlighted as an important analytical tool for secondary metabolite profiling, such as steroids, phenolics, and alkaloids, and can also identify sugars, fatty acids, amino acids, and other macromolecules found in plants and nonplant sources [33–36]. Identifying the bioactive profile may improve the identification of the key components responsible for various biological activities and contribute to the discovery of underlying principles of these effects.

To explore the possible mechanisms of action associated with the compounds identified from *S. villosa* and *V. patula*, we also performed molecular docking and absorption, distribution, metabolism, and excretion (ADME)/toxicity (T) studies to reveal the potential target(s) of the identified antioxidant components.

## 2. Materials and Methods

### 2.1. Chemicals

Phosphate buffer, potassium ferricyanide, trichloroacetic acid, ferric chloride, ascorbic acid,2,2-diphenyl-1-picrylhydrazyl (DPPH), and Folin-Ciocalteu Reagent were all purchased from Sigma-Aldrich, St. Louis, MO, USA.

### 2.2. Collection and Preparation of *S. villosa* and *V. patula* Extracts

Whole *S. villosa* and *V. patula* plants were collected from the Chittagong Hill-Tracts region of Bangladesh, and plants were authenticated and identified by a renowned taxonomist from the Bangladesh Council of Scientific and Industrial Research (BCSIR). The bark of *S. villosa* and the whole *V. patula* plant were washed with distilled water. The plant parts were cut into small pieces and dried. The dried materials were crushed into a fine pure powder using an electric blender. The powder was then stored in separate airtight containers. To obtain the extracts, the powders were placed in airtight containers, hexane was added at a sample to solvent ratio of 1 : 3, and the sample was subjected to uninterrupted stirring at 150 rpm for 90 minutes. The stirring was discontinued, and the sample was allowed to sit for 30 minutes, after which the hexane was decanted from the sample by filtration. Fresh hexane was added to the sample, and the process was repeated three times. After the final repetition, the hexane was decanted, and the sample was filtered under vacuum to completely remove all hexane, resulting in a defatted sample. The defatted crude powder was placed in an airtight container, and aqueous (95%) methanol was added at a sample to solvent ratio of 1 : 10. The sample was then subjected to 7 days of repeated 40/20-minute shaking/sonication cycles of uninterrupted agitation on a shaker machine at 150 rpm and ultrasonic vibrations in a sonicator machine at 55°C. The mixture was then filtered through Whatman #1 filter paper, and the filtrate was collected. This procedure was repeated thrice to extract all phytochemicals from the sample. All obtained filtrates were combined, and the methanol was evaporated in a rotary evaporator machine (Buchi, Postfach, Switzerland). The filtrates were lyophilized to complete dryness at −70°C in a freeze drier (SP Scientific, Stone Ridge, NY, USA), collected into a Petri dish, covered and wrapped properly, and stored at 4°C until further experiments.

### 2.3. GC-MS Analysis

The methanolic extract of *S. villosa* (MESV) and the methanolic extract of *V. patula* (MEVP) were evaluated in a mass spectrometer (TQ 8040, Shimadzu Corporation, Kyoto, Japan) using the electron impact ionization (EI) technique and a gas chromatograph (GC-17A, Shimadzu Corporation) with a merged silica capillary column (Rxi-5 ms; 0.25 m film, 30 m long and internal diameter 0.32 mm) coated with DB-1 (J&W). The oven temperature was set at 70°C (0 min); 10°C, 150°C (5 min); 12°C, 200°C (15 min); and 12°C, 220°C (5 min), with a clamp time of 10 min. The inlet temperature was 260°C. The flow rate of the column was 0.6 mL/min helium gas at constant pressure (90 kPa). The GC to MS interface temperature was 280°C. The MS was used in scanning mode, with a scanning range of 40–350 amu. The ionization mode was EI, and the mass range was 50–550 m/z. One microliter of the sample was injected in the splitless injection mode. The total GC-MS course time was 50 min. The compounds in the peak areas were classified by comparison with the national institute of standards and technology (NIST) GC-MS library version 08-S [37].

### 2.4. Antioxidant Activity

The experiments were performed in triplicate.

#### 2.4.1. 2,2-Diphenyl-1-Picrylhydrazyl (DPPH) Free Radical Scavenging Activity

The extracts were evaluated for antioxidant activity using DPPH, as described in the literature [38]. In this experiment, 3 mg of each extract was added to 1 mL 50% methanol (v/v), and ascorbic acid (0.3 g) stock solution was added to 1 mL 50% methanol (v/v) as a positive control. The serial dilution technique was applied to obtain MEVP, MESV, and ascorbic acid at concentrations of 500, 250, 125, 62.5, 31.25, and 15.625 *μ*g/mL. A 0.1 mL aliquot of each concentration of extract solution in methanol was combined with 1.0 mL freshly formulated DPPH-methanol solution (0.1 mM) and 0.45 mL 50  mM Tris (hydroxymethyl) aminomethane (THAM) hydrochloride buffer (pH 7.40). The reaction was allowed to develop for 30 minutes, and absorbances were estimated at 517 nm. The corresponding inhibition rates were measured using the following equation:

(1) $$\begin{array}{c} \text{DPPH scavenged}\left(\%\right)=\left[\frac{\left(A-B\right)}{A}\right]\times 100, \end{array}$$

where *A* is the absorbance in the presence of extract or standard and *B* is the absorbance of the control.

#### 2.4.2. Total Phenolic Content (TPC)

The total phenolic content (TPC) of MESV and MEVP was determined using an oxidizing agent, Folin-Ciocalteu Reagent (FCR), according to the method described by Ali Reza et al. [39]. A 1 mL volume of FCR was diluted in 9 mL purified water, and then, 2.5 mL diluted FCR was combined with 2.5 mL (20%) of Na_2_CO_3_ and 500 *μ*g/mL extract. Purified water was added to obtain a final volume of 10 mL. The solution was incubated for 20 min (250°C), and the absorbance was observed at 765  nm in triplicate. Gallic acid was used as the standard for the calculation of TPC, and the values were obtained according to the standard gallic acid curve (*y* = 0.0039*x* + 0.0406; *R*^2^ = 0.9981). TPC was determined according to the following equation, in gallic acid equivalents (GAE; mg/g):

(2) $$\begin{array}{c} \text{TPC}=\text{equivalent reagent}\left(\text{Conc.}\right)\times \frac{\text{volume of total content}}{\text{conc. of sample taken}}. \end{array}$$

#### 2.4.3. Total Flavonoid Content (TFC)

The total flavonoid content (TFC) of MESV and MEVP was evaluated as previously described by Ali Reza et al. [39]. The TFC was calculated by mixing 0.5 mL extract with 1.5 mL methanol and adding 0.1 mL AlCl_3_ (10%), 0.1 mL CH_3_CO_2_K (1 M), and 2.8 mL distilled water. The mixture was incubated at 25°C for 30 min, and later, the absorbance was taken at 415 nm. The blank solution contained all of the reagents except for the extract. The TFC calculation was measured in quercetin equivalents (QE; mg/g), using quercetin as the standard.

### 2.5. Statistical Analysis

Values are reported as the mean ± standard error of the mean (SEM; *n* = 3). ^*a*^*P* < 0.05, ^*b*^*P* < 0.01, and ^*c*^*P* < 0.001 are used to identify significant differences for extract values compared with those for ascorbic acid, two-way analysis of variance (ANOVA), followed by Dunnett's test.

### 2.6. In Silico Molecular Docking

#### 2.6.1. Protein Preparation

The 3D structure of urate oxidase (PDB: 1R4U) [40] and glutathione reductase (PDB: 3GRS) [41] was retrieved from the Protein Data Bank (*.pdb* format) [42] to evaluate the antioxidant effect. The 3D protein structure was assembled and refined using the method described by Uddin et al. [43].

#### 2.6.2. Ligand Preparation

Identified compounds from *S. villosa* and *V. patula* were obtained from the PubChem databases in SDF format. Ligprep (Schrödinger v11.1) was used to prepare the ligand by maintaining OPLS3 force field [44]. The possible ionization state was generated at specific pH values (7.0 ± 2.0).

#### 2.6.3. Receptor Grid Generation

Receptor grid generation was performed in Schrödinger v11.1, using the default parameters, with the van der Waals scaling factor and charge cutoff set to 1.00 and 0.25, respectively. A cubic box was placed on the geometrical center of the selected active site of the selected receptor, and a size setting of 14 Å × 14 Å × 14 Å was used for molecular docking.

#### 2.6.4. Glide Standard Precision (SP) Ligand Docking and MM-GBSA Calculation

Standard Precision (SP) flexible docking was performed using Glide (Schrödinger v11.1) [43, 45, 46]. The default parameters for the van der Waals scaling factor (0.80) and partial charge cutoff (0.15) were retained, and the docking score was recorded. The Schrödinger Prime MM-GBSA (OPLS3) was used for determining the binding energy of each ligand and the targeted receptor (kcal/mol) [47–49].

#### 2.6.5. In Silico Study: Determination of Pharmacokinetic Parameters by SwissADME

The pharmacokinetic parameters used to assess the drug-likeness properties of the identified compounds were determined using SwissADME (http://www.swissadme.ch/). An orally active drug requires that each of the compounds adheres to the drug-like properties established by Lipinski and Veber's rule [50].

#### 2.6.6. In Silico Study: Toxicological Properties Prediction by ProTox Webserver

The toxicological properties of the compounds were predicted with the assistance of the ProTox online server. The current study evaluated the toxicity profiles of selected compounds based on mutagenicity, carcinogenicity, hepatotoxicity, and toxicity class [51].

## 3. Results and Discussion

### 3.1. GC-MS Analysis

In a previous study, four triterpenoids were isolated and identified as bauerenyl acetate (I), friedelin (II), epifriedelanol (III), 20 (30)-taraxastene-3 betas, and 21 *α*-diol (IV) [30]. Several phytoconstituents from *S. villosa* and *V. patula* were identified in the current study. The MESV and MEVP may have potential therapeutic properties due to the presence of these bioactive phytoconstituents. The study was performed using GC-MS, one of the most widely used methods for phytoconstituent separation. The investigation of MESV and MEVP via GC-MS identified 52 and 33 phytochemical compounds, respectively (Tables 1 and 2). Figures S1 and S2 depict typical chromatograms for MESV and MEVP, respectively. Figures S3 and S4 depict the typical fragmentation pattern of compounds identified from MESV and MEVP, respectively. The major phytoconstituents in MESV included terpenoids, phytosterols, esters, acids, and other organic compounds. The major compounds isolated from MESV included mebutamate (RT: 24.538), octadecanoicacid, 2-hydroxy-1,3-propanediyl (RT: 23.790), estradiol (RT: 22.871), methotrexate (RT: 22.280), daucol (RT: 21.447), meprobamate (RT: 21.299), hexadecanal (RT: 20.515), glycerol 1-palmitate (RT: 20.134), and cyclohexane, eicosyl- (RT: 19.826). The major compounds isolated from MEVP included silanol, trimethyl-, phosphite (3 : 1) (RT: 31.157), androsta-3,5-dien-3-ol, 17-acetyl-3-O-(t-butyl 4-acetyl-3,5-dimethyl-2-pyrrolecarboxylate) (RT: 31.157), glucitol, 6-O-nonyl- (RT: 25.403), 1-eicosanol (RT: 24.546), nonanal (RT: 23.435), 3,3-dimethylpiperidine (RT: 21.935), epinephrine, (*β*)-, 3TMS derivative (RT: 21.914), undecanal (RT: 20.132),folic acid (RT: 20.190), and hexanal (RT: 19.670).

### 3.2. Antioxidant Activity

**Figure 1:**
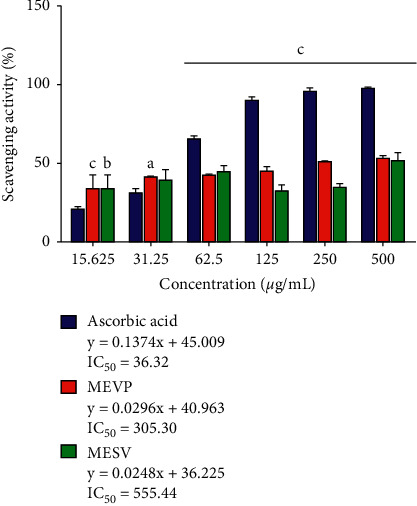
DPPH scavenging activity of methanolic extract of *Sterculia villosa* (MESV) and methanolic extract of *Vernonia patula* (MEVP) compared against the standard compound, ascorbic acid. Values are presented as the mean ± SEM (*n* = 3). ^*a*^*P* < 0.05, ^*b*^*P* < 0.01, and ^*c*^*P* < 0.001; significant compared with ascorbic acid (two-way ANOVA, followed by Dunnett's test).

Compared with the standard antioxidant, ascorbic acid, the inhibitory rate of MEVP gradually increased with increasing concentrations, ranging from 15.625 to 500 *μ*g/mL. A similar increase in inhibition was observed for increasing concentrations of MESV in the same concentration range. The maximum scavenging activity was observed at 500 *μ*g/mL for all three tested compounds, with values of 52.42%, 53.89%, and 98.33% for MESV, MEVP, and ascorbic acid, respectively (Figure 1). The IC_50_ values for MEVP, MESV, and ascorbic acid were determined to be 305.30, 555.44, and 36.32 *μ*g/mL, respectively.

The extracts were found to be potently active in the DPPH scavenging activity assay. The antioxidant activity of MESV and MEVP against DPPH is thought to be attributable to their hydrogen-donating capacity, suggesting that the extracts have the capacity to donate protons and can be used as primary antioxidants. The TPC and TFC were assessed using the regression equations for gallic acid (*y* = 0.0039*x* + 0.0406; *R*^2^ = 0.9981) and quercetin (*y* = 0.0102*x* − 0.0637; *R*^2^ = 0.9693), respectively. The TFC for MESV was higher (81.44 ± 2.70 mg QE/g dry extract) than the TPC (62.58 ± 1.93 mg GAE/g dry extract; Table 3). In MEVP, the TFC was higher (291.31 ± 6.61 mg QE/g dry extract) than the TPC (58.99 ± 3.16 mg GAE/g dry extract; Table 3).

Based on the TFC and TPC assays, the *S. villosa* and *V. patula* extracts contained significant amounts of flavonoid and phenolic contents, indicating that these plants' phenolic components may predominantly consist of flavonoids in glycosidic forms; glycosidic flavonoids tend to concentrate in polar solvents, which are more effective than less polar solvents for the removal of phenolic compounds from plant materials [52, 53]. Previous research showed that the presence of phenolic compounds, such as flavonoids, correlates with high levels of antioxidant activity and health benefits [54].

### 3.3. Molecular Docking Study

**Figure 2:**
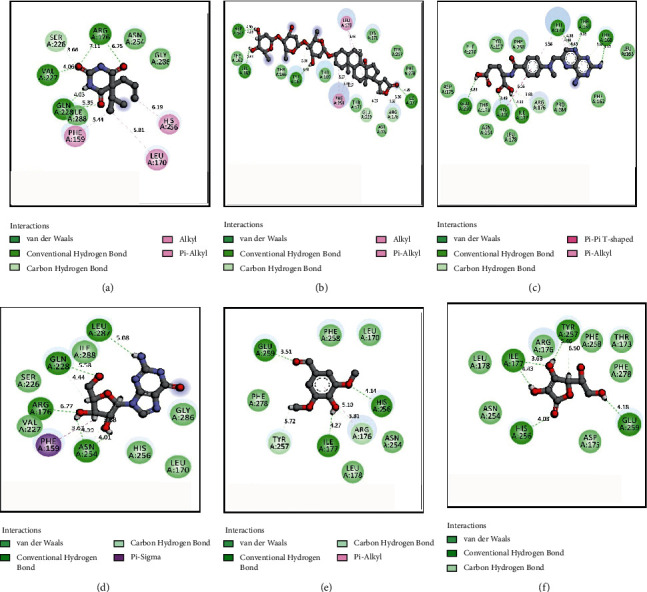
2D representations of the best docking scores between urate oxidase (PDB: 1R4U) and (a) aprobarbital: conventional hydrogen bond (ARG-176, VAL-227, GLN-228), van der Waals bond (ILE-288), and alkyl and pi-alkyl bond (PHE-159, LEU-170, HIS-256); (b) digitoxin: conventional hydrogen bond (ASP-165, LEU-163, TYR-167, and ILE-177), carbon–hydrogen bond (ARG-176, GLU-259, TYR-167, and ASP-165), and alkyl and pi-alkyl bond (LEU-170, PHE-258); (c) methotrexate: conventional hydrogen bond (GLU-259, HIS-256, ILE-177, THR-168, THR-169, and LEU-170), pi-pi T-shaped bond (HIS-256), and pi-alkyl bond (LEU-170); (d) guanosine: conventional hydrogen bond (LEU-287, ARG-176, and ASN-254), carbon–hydrogen bond (GLN-228, ASN-254), and pi-sigma bond (PHE-159); (e) benzaldehyde, 4-hydroxy-3,5-dimethoxy-: conventional hydrogen bond (HIS-256, ILE-177, and GLU-259), carbon–hydrogen bond (ARG-176), and pi-alkyl bond (ARG-176); (f) ascorbic acid (control): conventional hydrogen bond (TYR-257, ILE-177, HIS-256, and GLU-259) and carbon–hydrogen bond (TYR-257).

**Figure 3:**
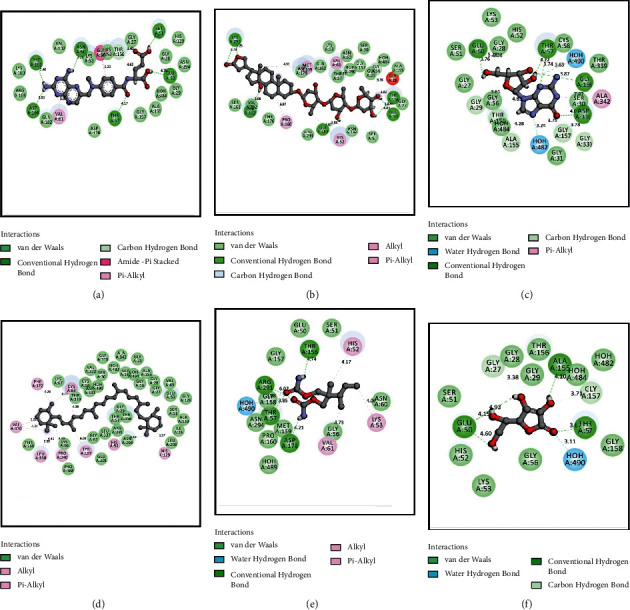
2D representations of the best docking scores between glutathione reductase (PDB: 3GRS) and (a) methotrexate: conventional hydrogen bond (SER-51, ASN-60, PHE-181, ASP-104, THR-57, and GLU-50), carbon–hydrogen bond (THR-156), amide-pi stacked bond (GLY-50), and pi-alkyl bond (VAL-61); (b) digitoxin: conventional hydrogen bond (THR-156, GLU-50, GLY-158, THR-162, and LYS-296), alkyl and pi-alkyl bond (MET-159, PRO-160, HIS-52, and VAL-61), and carbon–hydrogen bond (GLY-56); (c) guanosine: conventional hydrogen bond (GLY-158, THR-57, ASP-331, and GLU-50), water hydrogen bond (HOH-482), carbon–hydrogen bond (GLY-157, ALA-155, THR-156, GLY-29, GLU-50, and GLY-330), and pi-alkyl bond (ALA-342); (d) *β*-carotene: alkyl and pi-alkyl bond (LEU-338, VAL-370, PRO-340, TYR-197, CYS-63, HIS-52, and HIS-129); (e) mebutamate: conventional hydrogen bond (ASP-178, THR-57, ARG-291, and THR-156), water hydrogen bond (HOH-490), and alkyl and pi-alkyl bond (VAL-61, LYS-53, and HIS-52); (f) ascorbic acid (control): conventional hydrogen bond (ALA-155, THR-57, and GLU-50), water hydrogen bond (HOH-490), and carbon–hydrogen bond (GLY-157).

**Figure 4:**
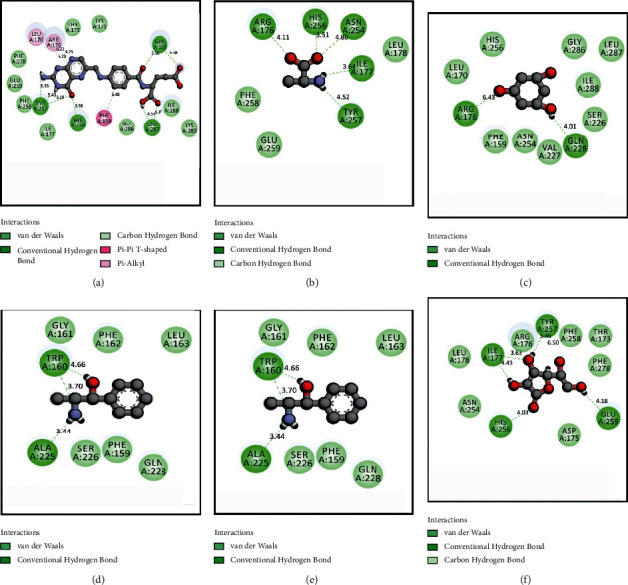
2D representations of the best docking scores between urate oxidase (PDB: 1R4U) and (a) folic acid: conventional hydrogen bond (GLN-228, LEU-287, HIS-256, and TYR-257), pi-alkyl bond (ARG-176), and pi-pi T-shaped bond (PHE-159); (b) D-alanine: conventional hydrogen bond (ARG-176, HIS-256, ASN-254, ILE-177, and TYR-257); (c) phloroglucitol: conventional hydrogen bond (ARG-176, GLN-228); (d) (−)-norephedrine: conventional hydrogen bond (TRP-160, ALA-225); (e) norpseudoephedrine: conventional hydrogen bond (TRP-160, ALA-225); (f) ascorbic acid (control): conventional hydrogen bond (TYR-257, ILE-177, HIS-256, GLU-259).

**Figure 5:**
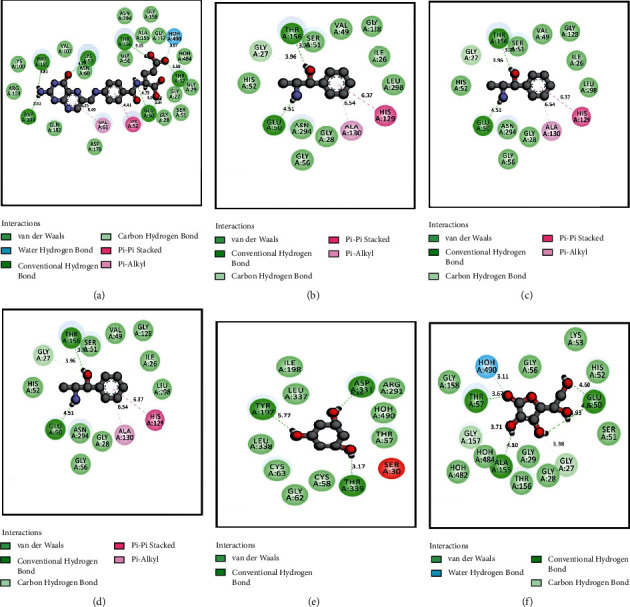
2D representations of the best docking scores between glutathione reductase (PDB: 3GRS) and (a) folic acid: conventional hydrogen bond (PHE-181, LYS-53, THR-156, THR-57, GLU-50, and ASP-104), water hydrogen bond (HOH-490), pi-pi stacked bond (HIS-52), and pi-alkyl bond (VAL-61); (b) (−)-norephedrine: conventional hydrogen bond (THR-156, GLU-50), pi-pi stacked bond (HIS-129), pi-alkyl bond (ALA-130), and carbon–hydrogen bond (GLY-27); (c) norpseudoephedrine: conventional hydrogen bond (THR-156, GLU-50), pi-pi stacked bond (HIS-129), pi-alkyl bond (ALA-130), and carbon–hydrogen bond (GLY-27); (d) cathine: conventional hydrogen bond (THR-156, GLU-50), pi-pi stacked bond (HIS-129), pi-alkyl bond (ALA-130), and carbon–hydrogen bond (GLY-27); (e) phloroglucitol: conventional hydrogen bond (ASP-331, TYR-197, and THR-339); (f) ascorbic acid (control): conventional hydrogen bond (GLU-50, ALA-155, THR-57), water hydrogen bond (HOH-490), and carbon–hydrogen bond (GLY-157, GLY-27).

The results for the molecular docking simulation study for the five compounds and control with the highest docking scores identified from *S. villosa* and *V. patula* extracts are shown in Tables 4 and 5, respectively. To evaluate the antioxidant attributes, the selected compounds from each plant extract were subjected to docking against urate oxidase (PDB: 1R4U) and glutathione reductase (PDB: 3GRS). For the compounds identified in *S. villosa*, the five selected compounds, ordered according to docking score for urate oxidase (PDB: 1R4U), were as follows: aprobarbital > digitoxin > methotrexate > guanosine > benzaldehyde, 4-hydroxy-3,5-dimethoxy-. Then, the order for docking scores when docked with glutathione reductase (PDB: 3GRS) in case of compounds identified in *S. villosa* is as follows: methotrexate > digitoxin > guanosine > *β*-carotene > mebutamate. For *V. patula* extract, the five selected compounds according to docking scores for urate oxidase (PDB: 1R4U) were as follows: folic acid > d-alanine > phloroglucitol > (−)-norephedrine ≥ norpseudoephedrine. When the docking simulation was carried out on the compounds from *V. patula* with glutathione reductase (PDB: 3GRS), the following order of docking score was found: folic acid > (−)-norephedrine ≥ norpseudoephedrine ≥ cathine > phloroglucitol. The molecular docking simulations between the selected compounds and the protein are further demonstrated in Tables 6 and 7. Furthermore, the 2D representations of the ligand–protein interactions are presented in Figures 2 and 3 for compounds in *S. villosa* and in Figures 4 and 5 for compounds in *V. patula*.

### 3.4. ADEME and Toxicological Study

The ADME properties are markers of pharmacokinetic characteristics and were used to assess oral bioavailability based on the Lipinski and Veber rules. The data for each compound were retrieved from the SwissADME online server, as shown in Tables 8 and 9 for *S. villosa* and *V. patula*, respectively. Digitoxin was found to violate three of the parameters, and *β*-carotene violated two parameters, while one parameter of the Lipinski rule was violated by methotrexate and guanosine, among compounds identified in *S. villosa*. Only folic acid, which violated one parameter, was found to violate these rules among the compounds identified in *V. patula*.

The toxicity profiles for each of the selected compounds in the two extracts are evaluated through the ProTox online server and are presented in Tables 10 and 11. These results showed that none of the selected compounds from *S. villosa* are associated with the hepatotoxic property. *β*-carotene was found to have mutagenicity while two of the compounds, namely, aprobarbital and mebutamate, were associated with carcinogenic properties among the compounds identified in *V. patula*.

In this study, molecular docking simulations were performed to associate and reciprocate the in vitro experimental findings. The extracts from *S. villosa* and *V. patula* were subjected to GC-MS to identify chemical compounds, and those compounds with potential pharmacological activity were subjected to a molecular docking simulation against urate oxidase (PDB: 1R4U) and glutathione reductase (PDB: 3GRS) to evaluate the antioxidant properties in silico. The five compounds from each plant extract with the highest docking scores against two distinctive receptors were selected for further analysis. Aprobarbital had the highest docking score (−6.266) among the compounds identified in *S. villosa* when the docking simulation was preceded against urate oxidase (PDB: 1R4U). It exhibits a better score than the control, ascorbic acid (−4.655). Aprobarbital formed conventional hydrogen bonds with the following amino acid residues in urate oxidase: ARG-176, VAL-227, and GLN-228. Aprobarbital also forms alkyl and pi-alkyl bonds with HIS-256, LEU-170, and PHE-159 and a carbon–hydrogen bond with SER-226 when binding with the active site of urate oxidase to exert an antioxidant effect. When the compounds were docked against glutathione reductase and compared to the control, methotrexate was found to have a significant binding affinity (−8.457). When bonded with methotrexate, it formed a conventional hydrogen bond with SER-51, ASN-60, PHE-181, ASP-104, THR-57, and GLU-50. One carbon–hydrogen bond with THR-156, one amide-pi stacked bond with GLY-50), and one pi-alkyl bond with VAL-61 were also reported. Folic acid possessed the best result (−6.038) for the compounds identified in *V. patula* in case of urate oxidase (PDB: 1R4U). Folic acid formed conventional hydrogen bonds with TYR-257, HIS-256, LEU-287, and GLN-228. A pi-pi bond was formed with PHE-159, and a pi-alkyl bond was formed with LEU-170 and ARG-176. Folic acid also possessed better ligand–protein interaction (−8.243) in case of glutathione reductase (PDB: 3GRS). It has formed a conventional hydrogen bond with PHE-181, LYS-53, THR-156, THR-57, GLU-50, and ASP-104, one water hydrogen bond with HOH-490, one pi-pi stacked bond with HIS-52, and one pi-alkyl with VAL-61. All of the compounds having the highest docking score were significantly better than the control in terms of ligand–protein interaction. This suggested that these compounds have good potential as a promising antioxidant agent. All of the compounds were also subjected to the evaluation of ADME and toxicological properties. The Lipinski rule of five states that orally administered agents should have the following properties: molecular weight <500 amu, hydrogen bond acceptor sites <10, hydrogen bond donor sites <5, and lipophilicity value (Log *P*) ≤ 5. Veber's rules recommend a number of rotatable bonds ≤10 and topological polar surface area ≤140. According to these rules, a compound or potential medicinal agent cannot violate all of the parameters while still presenting good oral bioavailability [55, 56]. Among the two compounds with the highest docking scores identified for each plant species, aprobarbital did not violate any of the parameters, whereas folic acid and methotrexate had violated one parameter of the Lipinski rule of five, which is within the acceptable range. Thus, these compounds are considered safe for *in vivo* administration to an animal model. In the *S. villosa* extract, all other compounds, except digitoxin, methotrexate, guanosine, and *β*-carotene, also met all of the criteria for the Lipinski rule. In the *V. patula* extract, all compounds meet all components of the rules except for folic acid. Additionally, the toxicity prediction showed that none of the identified compounds in *S. villosa* is played hepatotoxicity. When it comes to carcinogenicity, all of the compounds were free from carcinogenic properties except aprobarbital and mebutamate. In addition to that, only *β*-carotene was associated with mutagenic properties. By contrast, in the *V. patula* extract, all of the compounds were free from mutagenic, carcinogenic, and hepatotoxic properties. Therefore, the selected compounds from *S. villosa* and *V. patula* may represent promising antioxidant agents, as further supported by the molecular docking study.

## 4. Conclusions

This study reported the potential antioxidant effects of methanolic bark extract of *S. villosa* and methanolic whole-plant extract of *V. patula*; this might be due to their chemical constituents. These chemical compounds may offer antioxidant activities, as assessed by the molecular docking study. Further advanced studies remain necessary to identify the potential compounds responsible for antioxidant activities displayed by these two plants, *S. villosa* and *V. patula*.

## Figures and Tables

**Table 1:** Tentative compounds identified from the methanolic extract of *Sterculia villosa* by gas chromatography-mass spectrometry (GC-MS) analysis.

Sl. no.	Name	Molecular formula	Nature	RT	m/z	Area

1	Heptanal	C_7_H_14_O	Aldehyde	4.146	44.00	879969
2	Benzaldehyde, 2-methyl	C_8_H_8_O	Aldehyde	4.872	120.00	359567
3	Glucitol, 6-O-nonyl	C_15_H_32_O_6_	Sugar alcohol	4.118	73.00	665996
4	L-Arabinitol	C_5_H_12_O_5_	Sugar alcohol	6.119	44.00	19425
5	*α*-Isomethyl ionone	C_14_H_22_O	Ketone	6.134	150.00	531076
6	Eucalyptol	C_10_H_18_O	Monoterpenoid	6.566	154.00	273748
7	Vanillin	C_8_H_8_O_3_	Phenolic aldehyde	7.246	151.00	375524
8	Prednisone	C_21_H_26_O_5_	Glucocorticoid	7.922	44.00	18826
9	Bioallethrin	C_19_H_26_O_3_	Ester	7.974	137.00	267302
10	Sorbitol	C_6_H_14_O_6_	Sugar alcohol	8.872	137.00	507045
11	*β*-D-glucopyranose, 4-O-*β*-D-galactopyranosyl	C_12_H_22_O	Carbohydrate	9.665	43.00	94028
12	Santolinatriene	C_10_H_16_	Hydrocarbon	9.314	180.00	285758
13	Vanillin, acetate	C_10_H_10_O_4_	Phenyl acetate	9.603	151.00	205675
14	Guanosine	C_10_H_13_N_5_O_5_	Purine nucleoside	9.603	151.00	205675
15	Trans-11-Tetradecenyl acetate	C_16_H_30_O_2_	Fatty acid	9.665	43.00	148058
16	D-Galactonic acid, *γ*-lactone	C_6_H_10_O_6_	Acid	10.092	73.00	77114
17	Isopulegol	C_10_H_18_O	Terpenoid alcohol	10.359	127.00	99917
18	Benzaldehyde, 4-hydroxy-3,5-dimethoxy	C_9_H_10_O_4_	Aldehyde	10.463	182.00	393615
19	Naphthalene, 2-butyldecahydro-	C_14_H_26_	Hydrocarbon	10.697	137.00	256786
20	3-buten-2-one, 3-methyl-4-(3,5,6-trimethyl-3-cyclohexen-1-yl)	C_14_H_22_O	Alkane	10.852	43.00	71620
21	Cis-p-Mentha-2,8-dien-1-ol	C_10_H_18_OS	Monoterpenoid	10.949	167.00	162613
22	Trans-Sesquisabinene hydrate	C_15_H_26_O	Sesquiterpenoid	10.949	167.00	162613
23	2-methoxy-6-methylaniline	C_8_H_11_NO	Amine	11.439	137.00	4066243
24	*β*-carotene	C_40_H_56_	Carotene	11.440	43.00	140490
25	Aprobarbital	C_10_H_14_N_2_O_3_	Barbiturate derivatives	11.697	167.00	179001
26	Spiro[3.4]octan-5-one	C_8_H_12_O	Ketone	12.006	124.00	283819
27	2-dodecen-1-yl(-)succinic anhydride	C_16_H_26_O_3_	Anhydride	12.130	196.00	61469
28	Phytol	C_20_H_40_O	Diterpene alcohol	12.520	43.00	22833
29	Digitoxin	C_41_H_64_O_13_	Dardenolide glycoside	12.520	43.00	22833
30	Chrysanthemic acid	C_10_H_16_O_2_	Fatty acid	13.455	44.00	26500
31	*n*-hexadecanoic acid	C_16_H_32_O_2_	Fatty acid	13.459	43.00	490715
32	*β*-Asarone	C_12_H_16_O_3_	Phenylpropanoid	14.068	208.00	181791
33	Benzenepropanoic acid, 2,5-dimethoxy	C_11_H_14_O_4_	Organic acid	14.166	167.00	1864637
34	Decanoic acid, 2,3-dihydroxypropyl ester	C_15_H_30_O_4_	Organic acid	14.167	43.00	135871
35	9,12-octadecadienoic acid, methyl ester, (E,E)	C_19_H_34_O_2_	Monodecanoylglycerol	15.180	67.00	321453
36	7-hexadecenoic acid, methyl ester, (Z)	C_17_H_32_O_2_	Fatty acid methyl ester	15.244	55.00	182330
37	Citronellol	C_10_H_20_O	Monoterpenoid	15.348	71.00	332135
38	Undec-10-ynoic acid	C_11_H_18_O_2_	Fatty acid	15.490	43.00	139829
39	Undecanal	C_10_H_21_CHO	Aldehyde	16.205	73.00	40669
40	6-octadecenoic acid, methyl ester, (Z)	C_19_H_36_O_2_	Fatty acid	17.385	44.00	11503
41	Dodecanal	C_12_H_24_O	Aldehyde	18.284	44.00	14479
42	Nerolidol	C_15_H_26_O	Sesquiterpene	19.001	69.00	148917
44	Cyclohexane, eicosyl	C_26_H_52_	Cycloalkane	19.826	83.00	225697
45	Glycerol 1-palmitate	C_19_H_38_O_4_	Monoacylglycerols	20.134	43.00	311470
46	Hexadecanal	C_16_H_32_O	Aldehyde	20.515	149.00	114505
47	Meprobamate	C_9_H_18_N_2_O_4_	Carbamate	21.299	83.00	196437
48	Daucol	C_15_H_26_O_2_	Oxanes	21.447	151.00	211187
49	Methotrexate	C_20_H_22_N_8_O_5_	Antimetabolites	22.280	44.00	20613
50	Estradiol	C_18_H_24_O_2_	Steroid	22.871	272.00	407509
51	Octadecanoic acid, 2-hydroxy-1,3-propanediyl ester	C_39_H_72_	Fatty acid	23.790	43.00	126991
52	Mebutamate	C_10_H_20_N_2_O_4_	Carbamate	24.538	207.00	124804
53	Androsta-3,5-dien-3-ol, 17-acetyl-3-O-(t-butyldimethylsilyl)	C_27_H_44_O_2_Si	Steroids	25.401	207.00	39398

**Table 2:** Tentative compounds identified from the methanolic extract of *Vernonia patula* by gas chromatography-mass spectrometry (GC-MS) analysis.

Sl. no.	Name	Molecular formula	Nature	RT	m/z	Area

1	Cystine	C_6_H_12_N_2_O_4_S_2_	Amino acid	8.235	44.00	13166
2	D-alanine	C_3_H_7_NO_2_	*α*-amino acid	9.045	44.00	18522
3	Propanamide	C_3_H_7_NO	Amide	9.630	44.00	13580
4	(−)-norephedrine	C_9_H_13_NO	Sympathomimetic agent	9.765	44.00	16785
5	Norpseudoephedrine	C_9_H_13_NO	Alkaloid	9.765	44.00	16785
6	Dl-phenylephrine	C_9_H_13_NO	Sympathomimetic amine	9.765	44.00	16785
7	Octodrine	C_8_H_19_ N	Amine	10.040	44.00	3381
8	1,2-ethanediamine, N-(2-aminoethyl)	C_4_H_13_N_3_	Amine	10.480	44.00	16968
9	Chlorodifluoroacetamide	C_2_H_3_ClFNO	Amide	11.250	44.00	7827
10	Cathine	C_9_H_13_NO	Alkaloid	11.916	44.00	9982
11	Phloroglucitol	C_6_H_6_O_3_	Polyphenol	12.666	44.00	31436
12	2-octynoic acid	C_8_H_12_O_2_	Fatty acid	12.945	44.00	39571
13	Glutaraldehyde	C_5_H_8_O_2_	Aldehyde	13.275	44.00	42367
14	Methyl stearate	C_19_H_38_O_2_	Fatty acid methyl ester	13.457	74.00	972212
15	Dibutyl phthalate	C_16_H_22_O_4_	Ester	13.454	44.00	30188
16	DL-cystine	C_6_H_12_N_2_O_4_S_2_	Amino acid	14.154	44.00	5343
17	Epinephrine, (*β*)-, 3TMS derivative	C₉H₁₃NO₃	Catecholamine	14.894	73.00	367947
18	1-Dodecyne	C_12_H_24_	Alkene	14.752	44.00	5199
19	10-Undecenal	C_11_H_20_O	Aldehyde	15.489	55.00	142292
20	Phytol	C_20_H_40_O	Diterpene alcohol	15.348	71.00	227781
21	Piperazine	C_4_H_10_N_2_	Amine	15.341	44.00	34992
22	D-Galactonic acid, *γ*-lactone	C_6_H_10_O_6_	Sugar acid	16.727	44.00	23032
23	3,3′-Iminobispropylamine	C_6_H_17_N_**3**_	Nitrile	18.149	44.00	34648
24	Glutaraldehyde	C_5_H_8_O_2_	Aldehyde	18.805	44.00	27858
25	Hexanal	C_6_H_12_O	Aldehyde	19.670	44.00	17611
26	Folic acid	C_19_H_19_N_7_O_6_	Vitamin	20.190	44.00	28606
27	Undecanal	C_11_H_22_O	Aldehyde	20.132	43.00	145198
28	Epinephrine, (*β*)-, 3TMS derivative	C_18_H_37_NO_3_Si_3_	Hormone	21.914	73.00	295654
29	3,3-dimethylpiperidine	C_7_H_15_ N	Alkaloid	21.935	44.00	6210
30	Nonanal	C_9_H_18_O	Aldehyde	23.435	44.00	12399
31	1-eicosanol	C_20_H_42_O	Fatty alcohol	24.546	59.00	7793425
32	Glucitol, 6-O-nonyl	C_15_H_32_O_6_	Alcohol	25.403	73.00	294332
33	Androsta-3,5-dien-3-ol, 17-acetyl-3-O-(t-butyl 4-acetyl-3,5-dimethyl-2-pyrrolecarboxylate)	C_13_H_19_NO_3_	Ester	31.157	207.00	67432

**Table 3:** Total phenolic content (TPC) and total flavonoid content (TFC) of methanolic extract of *Sterculia villosa* (MESV) and methanolic extract of *Vernonia patula* (MEVP).

Subject	TPC (mg GAE/g extract)	TFC (mg QE/g extract)
MESV	62.58 ± 1.93	81.44 ± 2.70
MEVP	58.99 ± 3.16	291.31 ± 6.61
Regression equation	*y* = 0.0039*x* + 0.0406; *R*^2^ = 0.9981	*y* = 0.0102*x* − 0.0637; *R*^2^ = 0.9693

**Table 4:** Molecular docking scores for identified compounds in *Sterculia villosa.*

Sl. no.	Compounds	1R4U (kcal/mol)	1R4U (MM-GBSA)	3GRS (kcal/mol)	3GRS (MM-GBSA)

1	Heptanal	—	—	−2.166	−25.325
2	Benzaldehyde, 2-methyl	−4.477	−26.2735	−5.789	−25.545
3	Glucitol, 6-O-nonyl	−3.25	−42.1814	−5.897	−62.624
4	L-Arabinitol	−3.335	−34.4304	−4.144	−28.63
5	*α*-Isomethyl ionone	−3.844	−33.9885	−5.359	−33.515
6	Eucalyptol	−4.195	−19.6369	−4.072	−12.525
7	Vanillin	−4.841	−29.0303	−5.561	−27.446
8	Prednisone	—	—	—	—
9	Bioallethrin	−2.75	−31.6861	−4.487	−35.818
10	Sorbitol	−2.968	−31.4369	−4.206	−36.397
11	*β*-D-glucopyranose, 4-O-*β*-D-galactopyranosyl	—	—	—	—
12	Santolinatriene	—	—	−3.349	−22.086
13	Vanillin, acetate	−4.874	−29.5427	−5.452	−35.791
14	Guanosine	−5.706	−42.3794	−7.029	−54.903
15	Trans-11-tetradecenyl acetate	+1.755	−35.467	−0.89	−54.627
16	D-galactonic acid, *γ*-lactone	−5.106	−37.522	−5.364	−42.959
17	Isopulegol	−3.824	−32.1878	−6.033	−37.453
18	Benzaldehyde, 4-hydroxy-3,5-dimethoxy	−5.606	−37.7501	−5.56	−29.723
19	Naphthalene, 2-butyldecahydro	—	—	−5.423	−28.689
20	3-buten-2-one, 3-methyl-4-(3,5,6-trimethyl-3-cyclohexen-1-yl)-	−3.845	−32.3274	−4.502	−32.991
21	Cis-p-mentha-2,8-dien-1-ol	−3.743	−21.7445	−4.478	−16.731
22	Trans-sesquisabinene hydrate	−3.878	−24.5584	−4.885	−31.788
23	2-methoxy-6-methylaniline	−4.43	−26.2716	−5.738	−24.997
24	*β*-carotene	−2.503	−48.5021	−6.133	−52.201
25	Aprobarbital	−6.266	−42.6018	−4.711	−23.22
26	Spiro[3.4]octan-5-one	−4.707	−24.2297	−4.684	−24.887
27	2-dodecen-1-yl(-)succinic anhydride	−1.704	−41.2219	−3.745	−53.132
28	Phytol	−1.004	−48.5855	−4.22	−36.0226
29	Digitoxin	−6.249	−61.3721	−7.396	−66.1619
30	Chrysanthemic acid	−3.833	−33.8038	−4.539	−32.3551
31	n-hexadecanoic acid	+0.47	−42.1025	−0.504	−44.2263
32	*β*-asarone	−4.783	−40.5895	−5.033	−34.2406
33	Benzenepropanoic acid, 2,5-dimethoxy	−3.937	−42.1905	−5.359	−39.4505
34	Decanoic acid, 2,3-dihydroxypropyl ester	+0.681	−44.3904	−0.803	−52.772
35	9,12-octadecadienoic acid, methyl ester, (E,E)	+0.566	−45.4668	−1.483	−55.2765
36	7-hexadecenoic acid, methyl ester, (Z)	+0.652	−44.276	−1.171	−55.5193
37	Citronellol	−1.556	−26.2224	−3.03	−24.686
38	Undec-10-ynoic acid	+2.834	−38.9058	+2.186	−42.1098
39	6-octadecenoic acid, methyl ester, (Z)	−0.038	−44.8963	−0.423	−53.3609
40	Dodecanal	+2.452	−35.2648	+1.413	−40.0126
41	Nerolidol	−0.608	−34.4431	−2.001	−42.6634
42	Cyclohexane, eicosyl	−1.655	−46.9117	−2.967	−44.6644
43	Glycerol 1-palmitate	−2.579	−43.5815	−5.126	−47.6573
44	Hexadecanal	+1.286	−43.6651	−0.438	−46.5658
45	Meprobamate	−5.174	−45.9961	−5.719	−42.9626
46	Daucol	—	—	—	—
47	Methotrexate	−5.849	−61.4026	−8.457	−58.4485
48	Estradiol	−5.068	−33.4644	−5.8	−33.9768
49	Octadecanoic acid, 2-hydroxy-1,3-propanediyl	—	—	−1.109	−51.6491
50	Mebutamate	−5.359	−40.317	−6.05	−29.296
51	Androsta-3,5-dien-3-ol, 17-acetyl-3-O-(t-butyl)	—	—	—	—
52	Ascorbic acid (control)	−4.655	−37.8208	−5.965	−33.9373

**Table 5:** Molecular docking scores for identified compounds in *Vernonia patula*.

Sl. No.	Compounds	1R4U (kcal/mol)	1R4U (MM-GBSA)	3GRS (kcal/mol)	3GRS (MM-GBSA)
1	Cystine	−4.03	−41.7648	−3.399	−36.8961
2	D-alanine	−5.953	−15.5837	−5.036	−15.1612
3	Propanamide	−2.634	−18.48	−3.847	−26.3392
4	(−)-norephedrine	−5.79	−25.9004	−6.047	−29.0084
5	Norpseudoephedrine	−5.79	−25.9004	−6.047	−29.0084
6	Dl-phenylephrine	−4.823	−31.1556	−5.544	−34.4102
7	Octodrine	−2.424	−26.1646	−3.653	−25.4794
8	1,2-ethanediamine, N-(2-aminoethyl)	−2.227	−19.1913	−3.182	−22.3522
9	Chlorodifluoroacetamide	−4.071	−15.6083	−5.072	−23.672
10	Cathine	−5.79	−25.9004	−6.047	−29.0084
11	Phloroglucitol	−5.858	−25.276	−5.854	−24.6614
12	2-octynoic acid	−2.58	−32.7105	−3.556	−29.6861
13	Glutaraldehyde	−3.026	−19.8291	−2.555	−25.0008
14	Methyl stearate	+1.332	−40.0523	−0.447	−51.2734
15	Dibutyl phthalate	−1.334	−37.2661	−2.763	−45.1367
16	Epinephrine, *β*-, 3TMS derivative	—	—	—	—
17	1-dodecyne	+5.184	−27.4357	+3.225	−39.3134
18	10-undecenal	+2.981	−32.3244	+2.797	−34.283
19	Phytol	−1.004	−48.5855	−4.22	−36.0226
20	Piperazine	−4.933	−18.7799	−4.204	−21.361
21	D-galactonic acid, *γ*-lactone	−5.106	−37.522	−5.364	−42.9587
22	3,3′-iminobispropylamine	—	—	—	—
23	Glutaraldehyde	−3.026	−19.8291	−2.555	−25.0008
24	Hexanal	−1.698	−24.1357	−2.712	−19.7671
25	Folic acid	−6.038	−49.7423	−8.243	−55.9741
26	Undecanal	+1.756	−32.3068	+1.482	−39.983
27	3,3-dimethylpiperidine	—	—	—	—
28	Nonanal	−1.868	−28.3973	−1.738	−32.1394
29	1-Eicosanol	+1.381	−54.2076	−2.03	−52.5786
30	Ascorbic acid (control)	−4.655	−37.8208	−5.965	−33.9373

**Table 6:** Interactions and bond distances between selected compounds identified in *Sterculia villosa *** **and the receptors following: urate oxidase (PDB: 1R4U) and glutathione reductase (PDB: 3GRS) binding sites.

Proteins	Ligands	Hydrogen bond interactions		Hydrophobic interactions	
		Amino acid residue	Distance (Å)	Amino acid residue	Distance (Å)
1R4U	Aprobarbital	ARG-176	2.27, 2.35	HIS-256	4.39
		VAL-227	1.89	LEU-170	5.20
		GLN-228	2.02, 2.02	PHE-159	4.13
		—	—	SER-226	3.66
	Digitoxin	LEU-163	2.09	ASP-165	2.69
		ASP-165	1.79	TYR-167	2.70
		TYR-167	2.17	PHE-258	5.49, 4.36, 4.21
		ILE-177	2.38	LEU-170	5.20, 5.39
		—	—	GLU-259	2.38
		—	—	ARG-176	2.83. 2.34
		—	—	TYR-167	2.70
		—	—	LEU-170	5.20. 5.39
	Methotrexate	GLU-259	3.06	HIS-256	4.72
		HIS-256	1.98	ARG-176	2.71
		ILE-177	1.88	LEU-170	4.81, 4.48, 4.44
		LEU-170	2.72	THR-168	2.72
		THR-169	2.54	—	—
		THR-168	2.14	—	—

	Guanosine	ASN-254	1.98. 2.01	PHE-159	2.69
		ARG-176	1.90	GLN-228	2.42
		GLN-228	1.96	—	—
		LEU-287	2.50	—	—
	Benzaldehyde, 4-hydroxy-3,5-dimethoxy	HIS-265	2.58	ARG-176	2.73, 5.03
		ILE-177	1.99	TYR-257	2.34
		GLU-259	2.14	—	—
	Ascorbic acid	TYR-257	5.46	TYR-257	6.50
		ILE-177	3.63, 4.43	—	—
		HIS-256	4.03	—	—
		GLU-259	4.18	—	—
3GRS	Methotrexate	PHE-181	5.46	VAL-61	5.99, 4.88
		ASP-104	3.81	GLY-50	5.20
		ASN-60	3.18	THR-156	4.62, 3.40
		THR-57	4.17	—	—
		GLU-50	4.76	—	—
		SER-51	3.01	—	—
	Digitoxin	LYS-296	5.25, 4.24	MET-159	4.99
		THR-162	3.08	PRO-160	6.97
		GLY-158	3.93	HIS-158	4.87
		THR-156	4.50	VAL-61	5.19
		GLU-50	3.87	GLY-56	3.78
		—	—	GLU-50	4.43
		—	—	HIS-52	4.87
	Guanosine	GLU-50	3.76	GLU-50	5.28, 5.38
		HOH-482	3.24	GLY-56	3.61
		ASP-331	4.05	THR-156	4.85
		GLY-158	3.87	ALA-155	4.28
		THR-57	4.22, 3.74	GLY-157	3.75
		HOH-490	3.63	GLY-330	3.78
		—	—	ALA-342	7.47
	*β*-Carotene	—	—	CYS-63	4.63, 4.21
		—	—	PHE-372	6.26
		—	—	VAL-370	5.21, 4.21
		—	—	LEU-338	5.55, 4.45
		—	—	PRO-340	4.49
		—	—	TYR-197	6.03
		—	—	HIS-52	6.18
		—	—	HIS-129	5.57
	Mebutamate	THR-156	4.74	VAL-61	4.73
		ARG-291	6.02	LYS-53	4.06
		HOH-490	3.68	HIS-52	4.17
		THR-57	3.85	—	—
		ASP-178	4.21	—	—
	Ascorbic acid	GLU-50	4.60, 4.15, 4.93	GLY-157	3.71
		HOH-490	3.11	GLY-27	3.38
		THR-57	3.67	—	—
		ALA-155	4.10	—	—

**Table 7:** Interactions and bond distances between selected compounds identified in *Vernonia patula* and the receptors following: urate oxidase (PDB: 1R4U) and glutathione reductase (PDB: 3GRS) binding sites.

Proteins	Ligands	Hydrogen bond interactions		Hydrophobic interactions	
		Amino acid residue	Distance (Å)	Amino acid residue	Distance (Å)
1R4U	Folic acid	TYR-257	2.18, 1.94, 2.62	LEU-170	5.08
		HIS-256	2.54	ARG-176	4.60, 4.96
		LEU-287	1.70, 2.00	—	—
		GLN-288	2.24, 2.36	—	—
	D-alanine	ARG-176	4.11	—	—
		HIS-256	3.51	—	—
		ASN-254	4.86	—	—
		ILE-177	3.64	—	—
		TYR-257	4.52	—	—
	Phloroglucitol	ARG-176	6.43	—	—
		GLN-228	4.01	—	—
	(−)-norephedrine	TRP-160	1.79, 2.04	—	—
		ALA-225	1.97	—	—
	Norpseudoephedrine	TRP-160	1.79, 2.04	—	—
		ALA-225	1.97	—	—
	Ascorbic acid	HIS-256	4.03	TYR-257	6.50
		GLU-259	4.18	—	—
		TYR-257	5.46	—	—
		ILE-177	4.43, 3.63	—	—
3GRS	Folic acid	PHE-181	4.83	VAL-61	6.25, 5.49
		ASP-104	3.62	HIS-52	4.41
		LYS-53	4.79	GLU-50	4.67
		GLU-50	4.75, 3.36	—	—
		THR-57	3.59	—	—
		THR-156	5.15	—	—
		HOH-490	3.67	—	—
	(−)-norephedrine	THR-156	3.73	GLY-27	3.96
		GLU-50	4.51	ALA-130	6.54
		—	—	HIS-129	6.37
	Norpseudoephedrine	THR-156	3.73	GLY-27	3.96
		GLU-50	4.51	ALA-130	6.54
		—	—	HIS-129	6.37
	Cathine	THR-156	3.73	GLY-27	3.96
		GLU-50	4.51	ALA-130	6.54
		—	—	HIS-129	6.37
	Phloroglucitol	TYR-197	5.77	—	—
		THR-339	3.17	—	—
		ASP-331	4.49	—	—
	Ascorbic acid	THR-57	3.67	GLY-157	3.71
		ALA-155	4.10	GLY-27	3.38
		GLU-50	4.93, 4.15	—	—
		HOH-490	3.11	—	—

**Table 8:** Physicochemical properties associated with good oral bioavailability for the isolated compounds from *Sterculia villosa*.

Compounds	Lipinski rules				Lipinski's violations	Veber rules	
	MW	HBA	HBD	Log *P*		nRB	TPSA
	<500	<10	<5	≤5	≤1	≤10	≤140
Aprobarbital	210.23	3	2	0.82	0	3	75.27
Digitoxin	764.94	13	5	2.61	3	7	182.83
Methotrexate	454.44	9	5	−0.50	1	10	210.54
Guanosine	283.24	7	5	−2.02	1	2	159.51
Benzaldehyde, 4-hydroxy-3,5-dimethoxy	182.17	4	1	0.93	0	3	55.76
Mebutamate	232.28	4	2	1.05	0	8	104.64
*β*-carotene	536.87	0	0	11.11	2	10	0

Here, MW, molecular weight (g/mol); HBA, hydrogen bond acceptor; HBD, hydrogen bond donor; Log *P*, lipophilicity; nRB: number of rotatable bonds; TPSA: topological polar surface area.

**Table 9:** Physicochemical properties associated with good oral bioavailability for the isolated compound from *Vernonia patula*.

Compounds	Lipinski rules				Lipinski's violations	Veber rules	
	MW	HBA	HBD	Log *P*		nRB	TPSA
	<500	<10	<5	≤5	≤1	≤10	≤140
Folic acid	441.40	9	6	−0.36	1	10	213.28
D-alanine	89.09	3	1	−1.46	0	1	63.32
(−)-norephedrine	151.21	2	2	1.11	0	2	46.25
Norpseudoephedrine	151.21	2	2	1.11	0	2	46.25
Cathine	151.21	2	2	1.11	0	2	46.25
Phloroglucitol	132.16	3	3	−0.30	0	0	60.69

Here, MW, molecular weight (g/mol); HBA, hydrogen bond acceptor; HBD, hydrogen bond donor; Log *P*, lipophilicity; nRB: number of rotatable bonds; TPSA: topological polar surface area.

**Table 10:** Toxicological properties of the identified compounds from *Sterculia villosa.*

Compounds	Mutagenicity	Carcinogenicity	Hepatotoxicity	Toxicity class
Aprobarbital	NM	Carcinogenic	NH	II
Digitoxin	NM	NC	NH	I
Methotrexate	NM	NC	NH	I
Guanosine	NM	NC	NH	II
Benzaldehyde, 4-hydroxy-3,5-dimethoxy-	NM	NC	NH	IV
Mebutamate	NM	Carcinogenic	NH	IV
*β*-carotene	Mutagenic	NC	NH	IV

NM: nonmutagenic; NC: noncarcinogenic; NH: nonhepatotoxic; Class I (LD50 ≤ 5); Class II (5 < LD50 ≤ 50); Class III (50 < LD50 ≤ 300); Class IV (300 < LD50 ≤ 2000); Class V (2000 < LD50 ≤ 5000); Class VI (LD50 > 5000).

**Table 11:** Toxicological properties of the identified compounds from *Vernonia patula.*

Compound	Mutagenicity	Carcinogenicity	Hepatotoxicity	Toxicity class
Folic acid	NM	NC	NH	III
D-alanine	NM	NC	NH	III
(−)-norephedrine	NM	NC	NH	III
Norpseudoephedrine	NM	NC	NH	III
Cathine	NM	NC	NH	III
Phloroglucitol	NM	NC	NH	VI

NM: nonmutagenic; NC: noncarcinogenic; NH: nonhepatotoxic; Class I (LD50 ≤ 5); Class II (5 < LD50 ≤ 50); Class III (50 < LD50 ≤ 300); Class IV (300 < LD50 ≤ 2000); Class V (2000 < LD50 ≤ 5000); Class VI (LD50 > 5000).

## Data Availability

Available data are presented in the manuscript.

## Supplementary Materials

Supplementary Materials The following are available online at http://www.mdpi.com/xxx/s1. Figure S1: total ionic chromatogram (TIC) of methanol extract of Sterculia villosa (MESV). Figure S2: total ionic chromatogram (TIC) of methanol extract of Vernonia patula (MEVP). Figure S3: fragmentation pattern of compounds identified from the methanol extract of Sterculia villosa (MESV). Figure S4: fragmentation pattern of compounds identified from the methanol extract of Vernonia patula (MEVP).
