# Supplementary material for: Phytochemical Profiling, Antioxidant Activity, and In Silico Analyses of Sterculia villosa and Vernonia patula
Source: Evid Based Complement Alternat Med. 2022 Jun 6;2022:3190496. doi: 10.1155/2022/3190496 (PMC9192300; doi:10.1155/2022/3190496)
Supplement: Supplementary Materials — The following are available online at http://www.mdpi.com/xxx/s1. Figure S1: total ionic chromatogram (TIC) of methanol extract of Sterculia villosa (MESV). Figure S2: total ionic chromatogram (TIC) of methanol extract of Vernonia patula (MEVP). Figure S3: fragmentation pattern of compounds identified from the methanol extract of Sterculia villosa (MESV). Figure S4: fragmentation pattern of compounds identified from the methanol extract of Vernonia patula (MEVP). [file 3190496.f1.docx]

**Supplementary Materials**

Phytochemical Profiling, Antioxidant Activity, and In Silico Analyses of *Sterculia villosa* and *Vernonia patula*

Chadni Lyzu ^1^, Saikat Mitra ^2^, Zidan Khan ^3^, Abu Montakim Tareq ^3^, Ameer Khusro ^4^, Evena Parvin Lipy ^1^, Dipa Islam ^1^, Mahmuda Hakim ^1^ and Talha Bin Emran ^5,^*

^1^ Biomedical and Toxicological Research Institute, Bangladesh Council of Scientific and Industrial Research (BCSIR), Dr. Qudrat-I-Khuda Road, Dhanmondi, Dhaka 1205, Bangladesh; tithy_bmb@yahoo.com (C.L.), evena80@yahoo.com (E.P.L.), dipa_ifst@yahoo.com (D.I.), mahmuda.silvi@yahoo.com (M.H.)

^2^ Department of Pharmacy, Faculty of Pharmacy, University of Dhaka, Dhaka 1000, Bangladesh; saikatmitradu@gmail.com (S.M)

^3^ Department of Pharmacy, International Islamic University Chittagong, Chittagong 4318,

Bangladesh; zidankhan9090@gmail.com (Z.K.), montakim0.abu@gmail.com (A.M.T.)

^4^ Research Department of Plant Biology and Biotechnology, Loyola College, Chennai, Tamil Nadu, India; armankhan0301@gmail.com (A.K.)

^5^ Department of Pharmacy, BGC Trust University Bangladesh, Chittagong 4381, Bangladesh

* Correspondence: talhabmb@bgctub.ac.bd; Tel.: +88-01819-942214


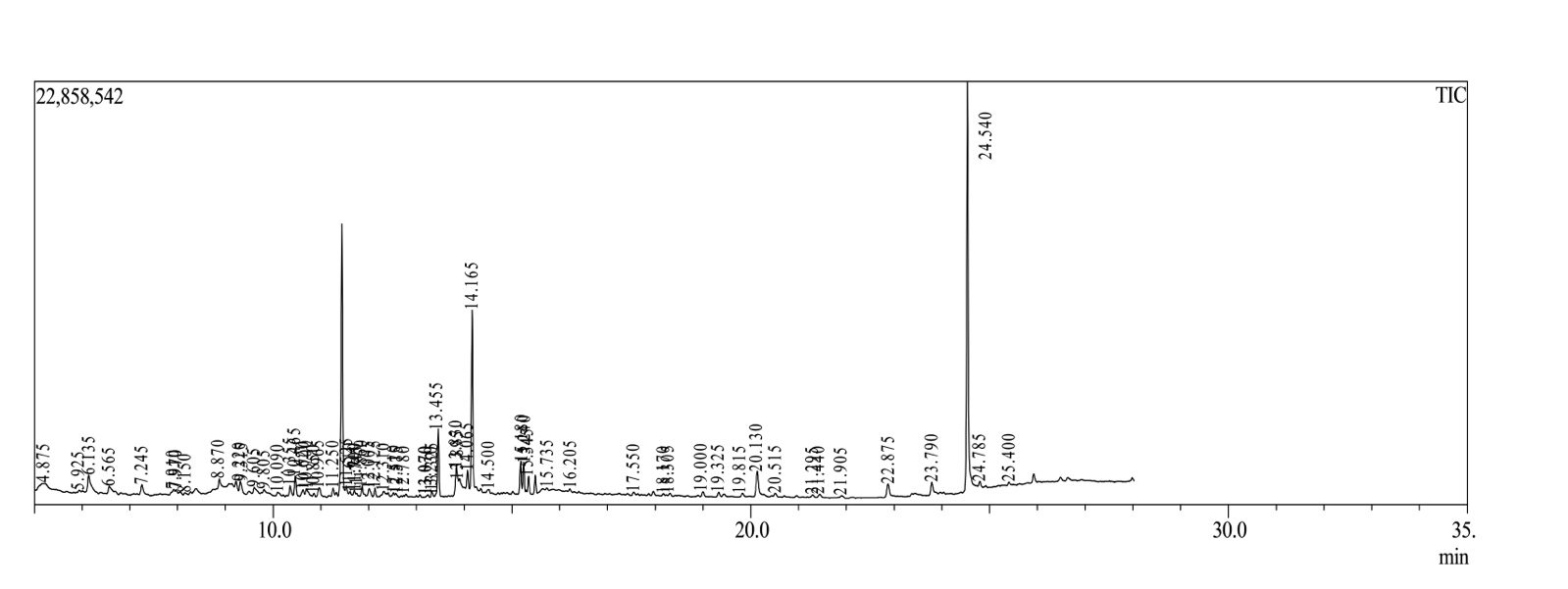


**Figure 1.** Total ionic chromatogram (TIC) of methanol extract of *Sterculia villosa* (MESV).


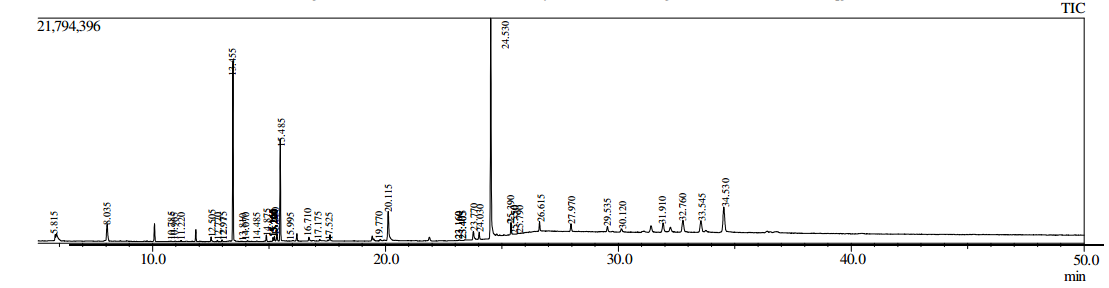


**Figure 2.** Total ionic chromatogram (TIC) of methanol extract of *Vernonia patula* (MEVP)

| Heptanal | 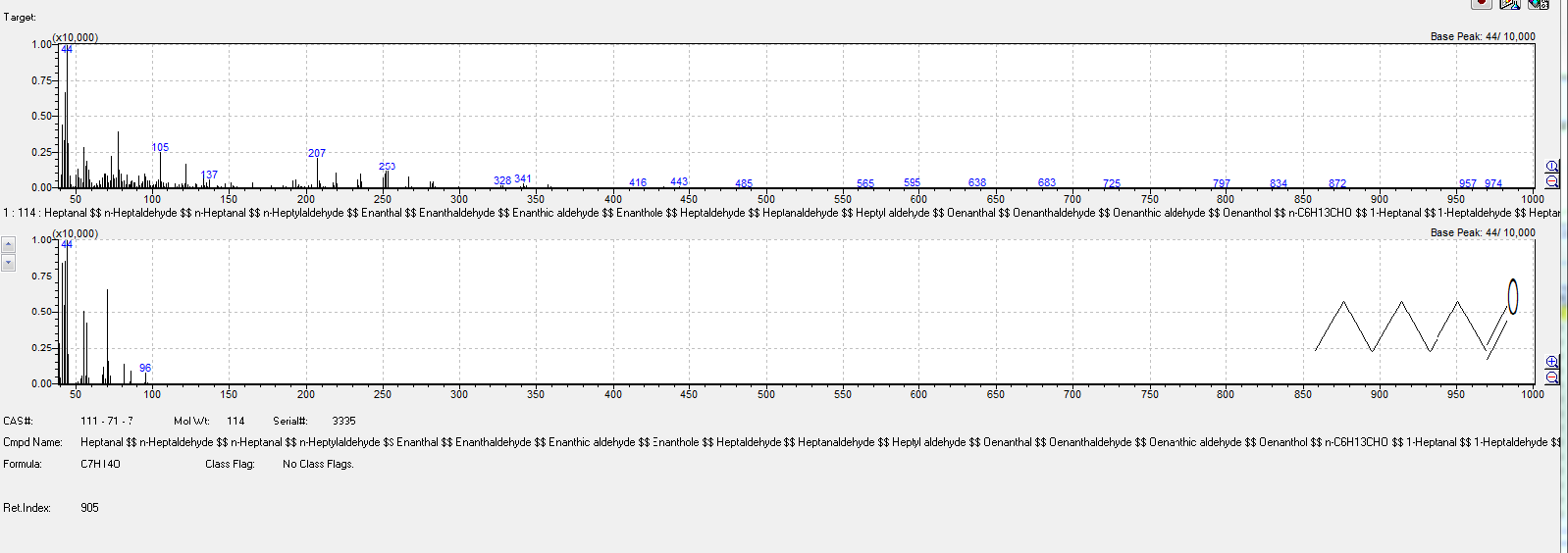 |
| --- | --- |
| Benzaldehyde-2-methyl | 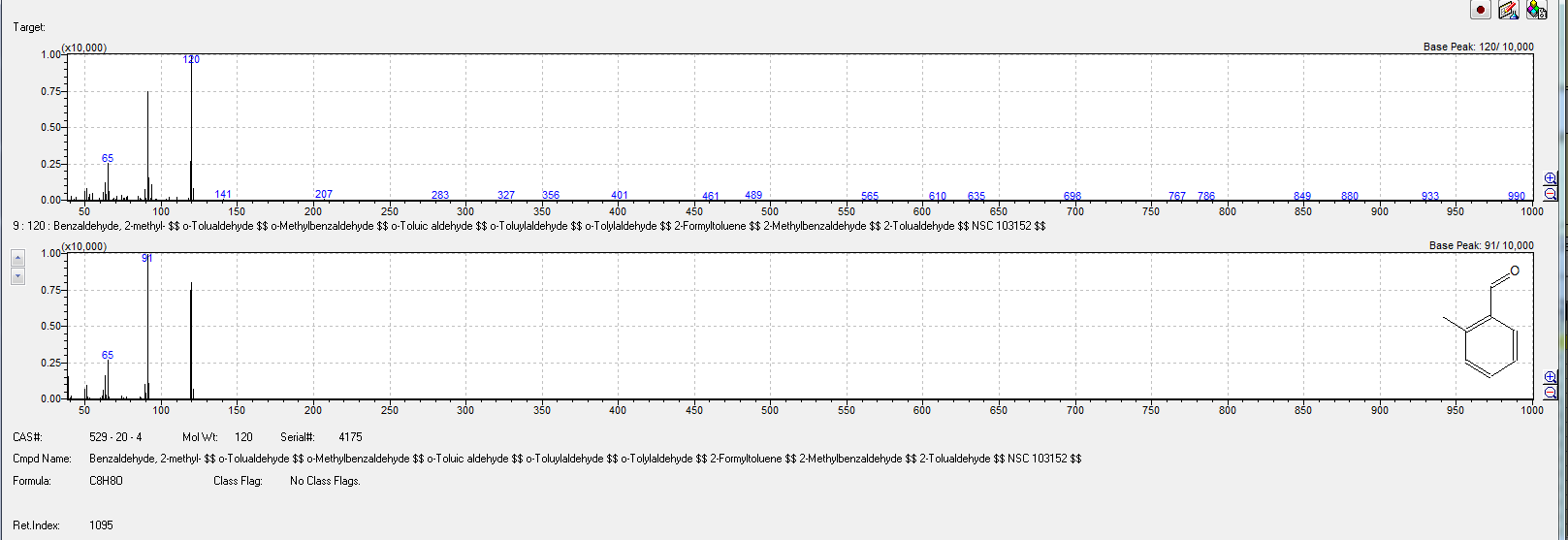 |
| Glucitol | 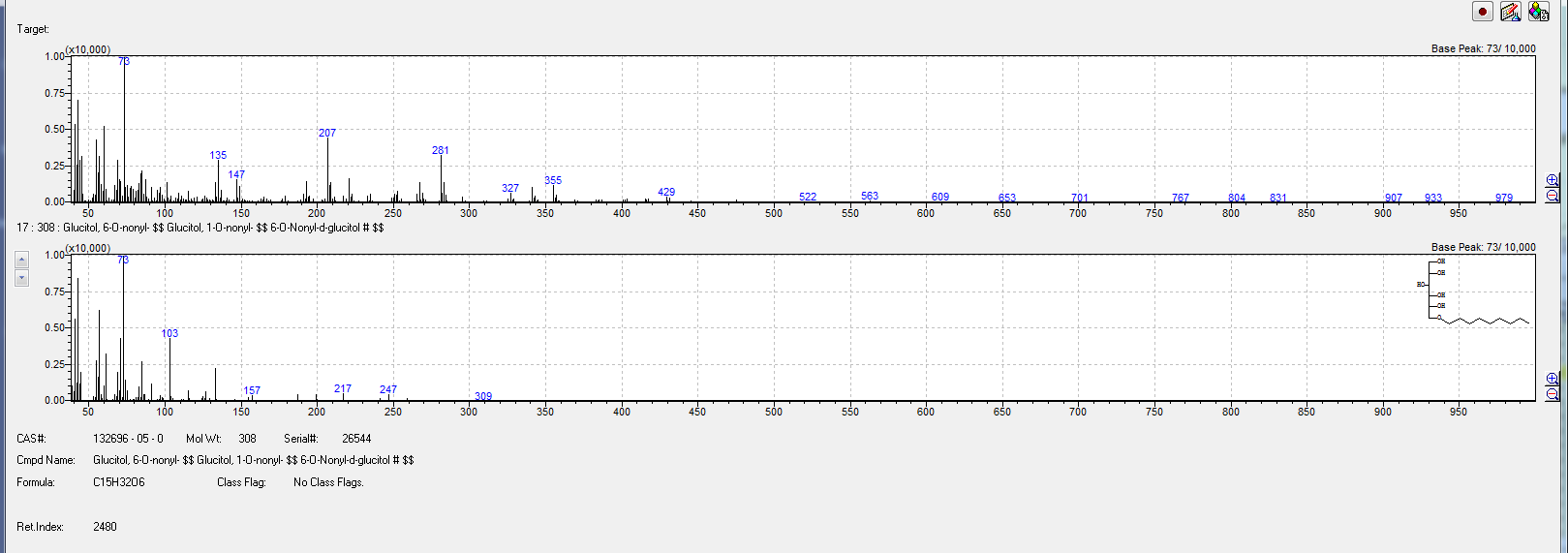 |
| L-Arabinitol | 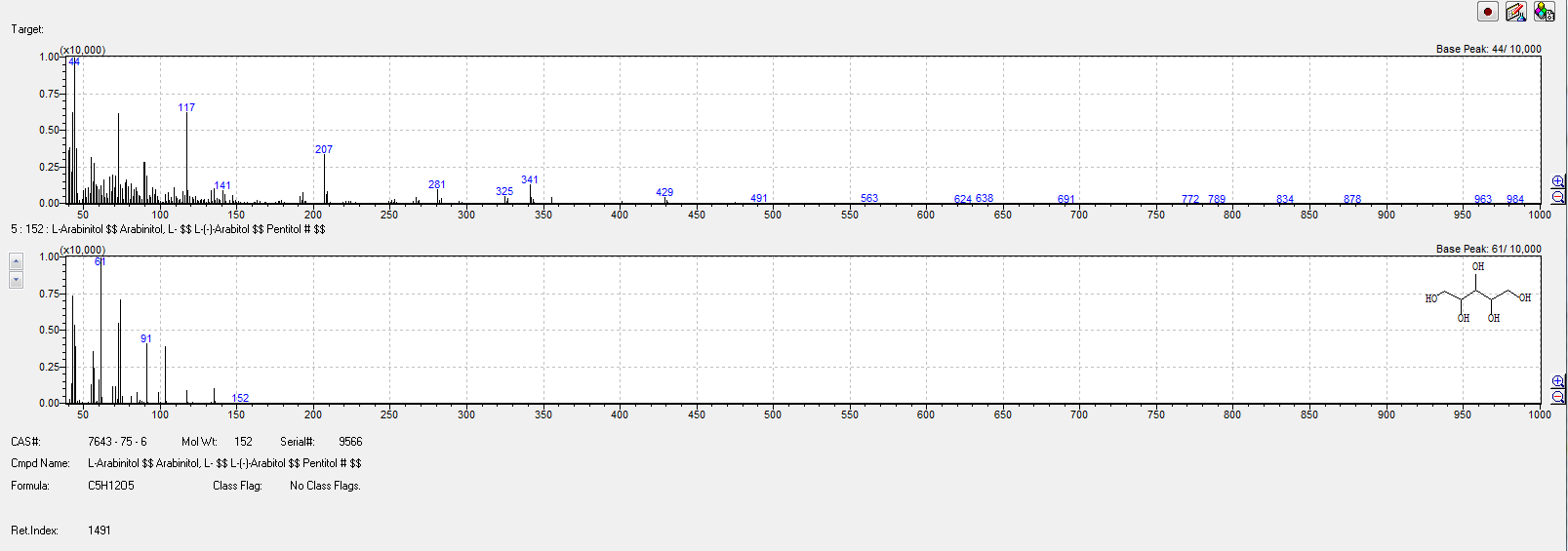 |
| alpha.Isomethyl Ionone | 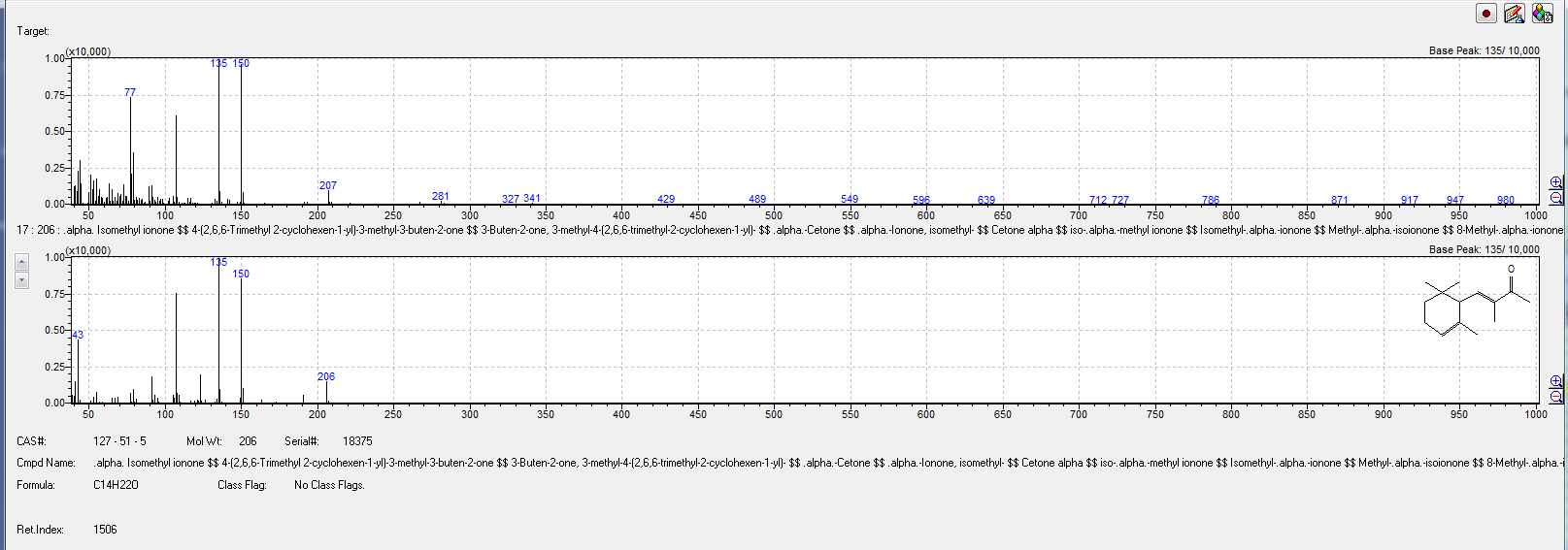 |
| Eucalyptol | 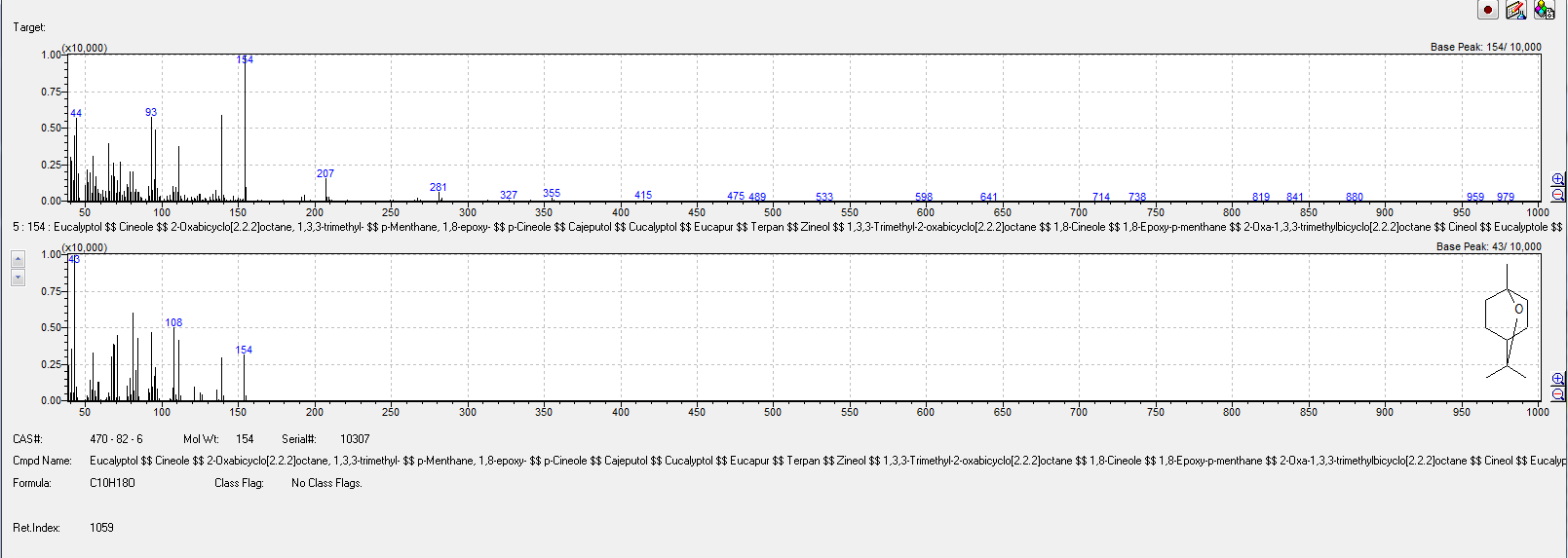 |
| Vanillin | 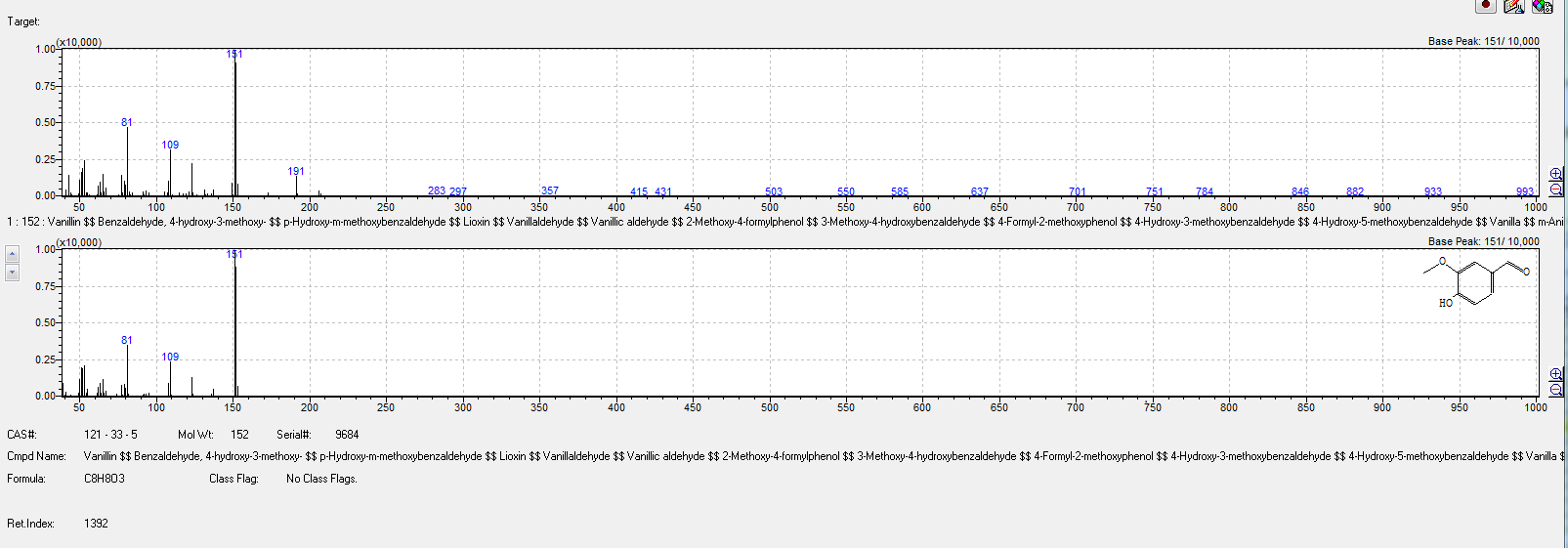 |
| Prednisone | 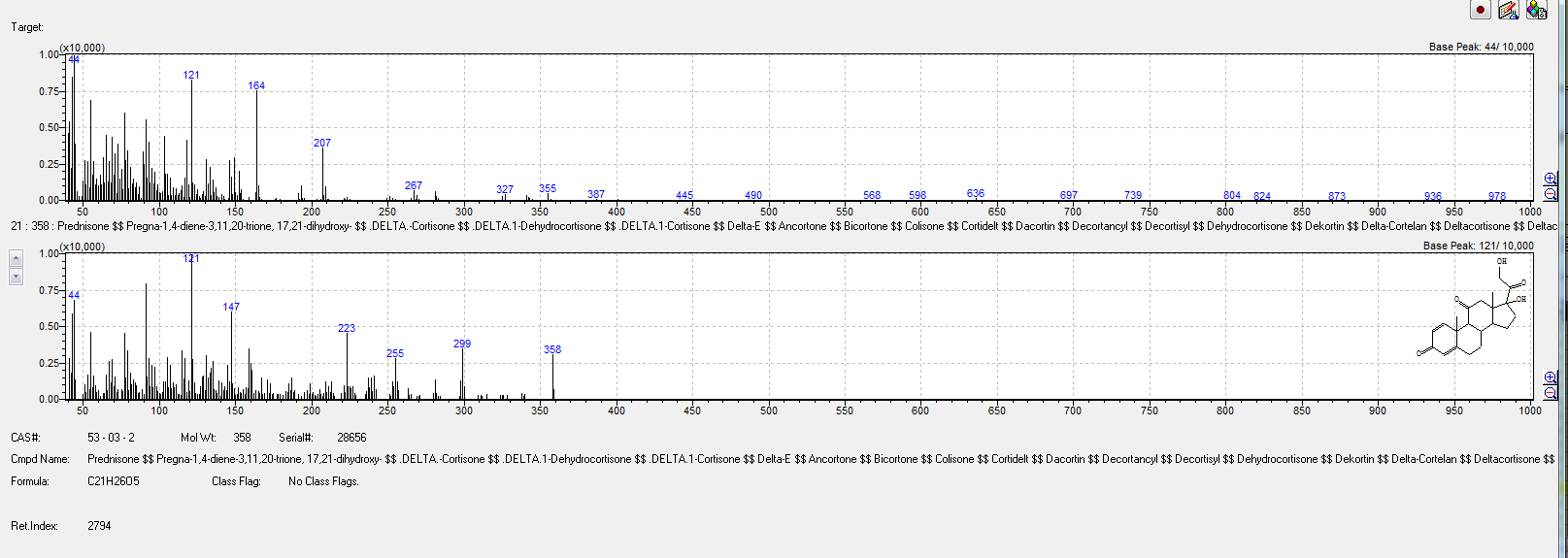 |
| Bioallethrin | 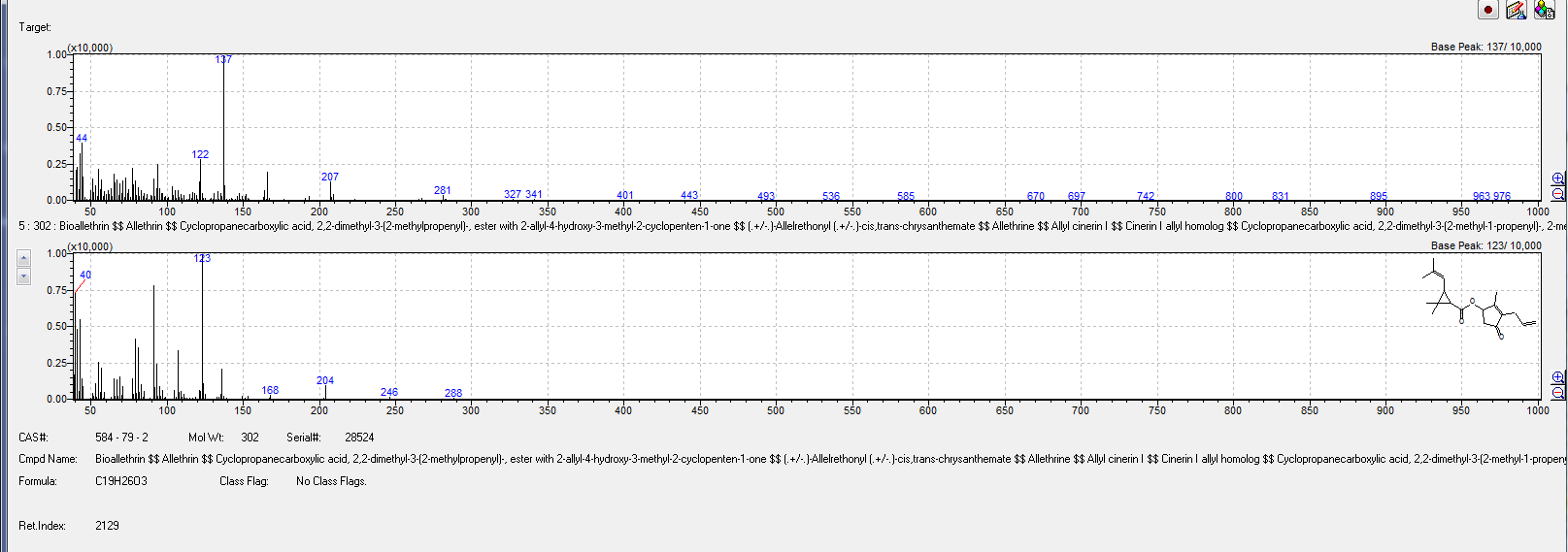 |
| Sorbitol | 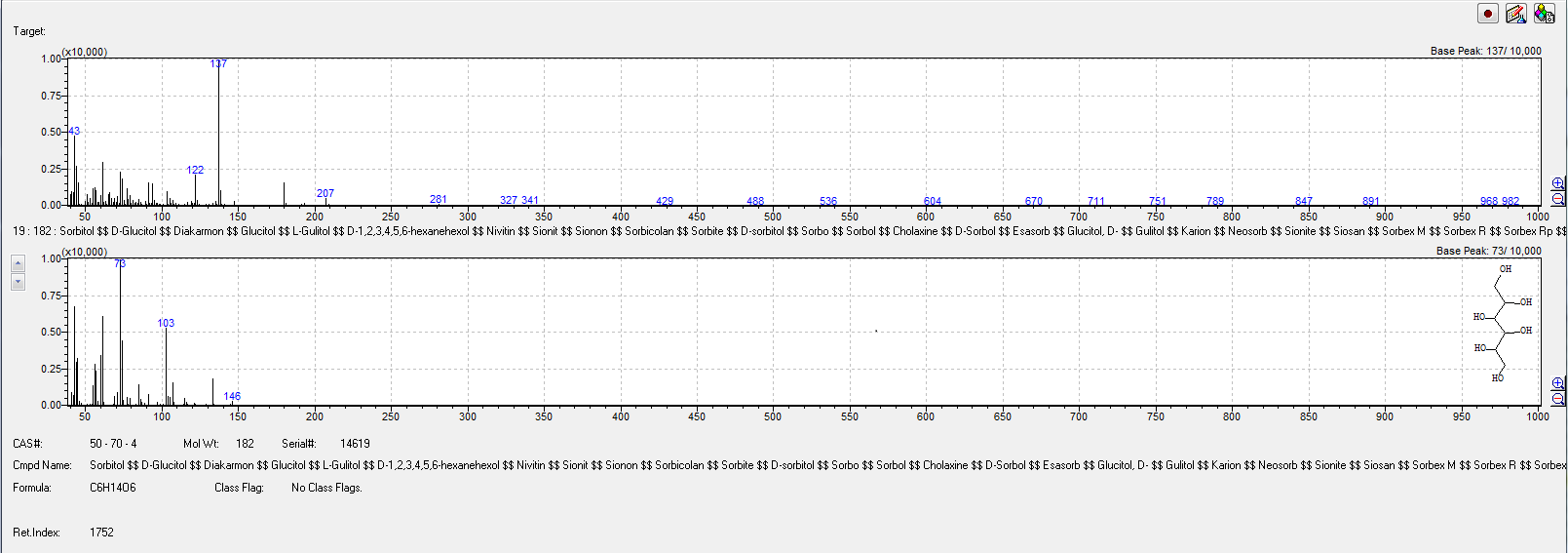 |
| Beta-D-Glucopyranose | 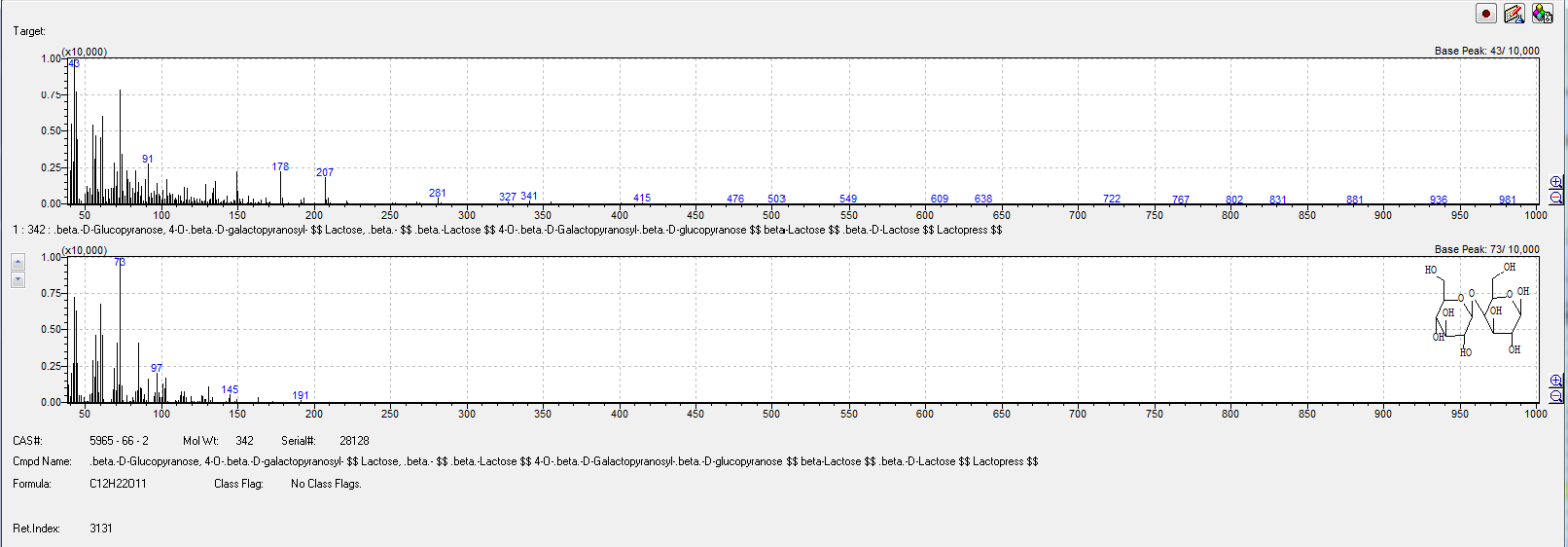 |
| Santolina triene | 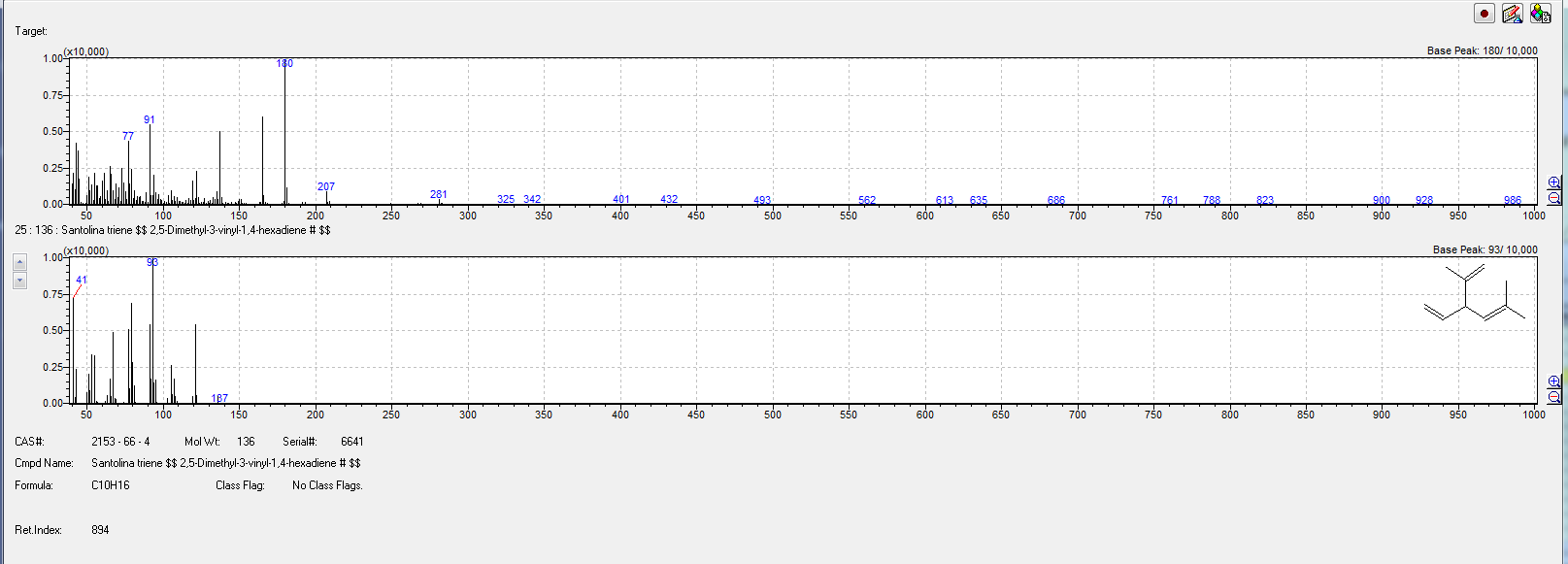 |
| Vanillin, acetate | 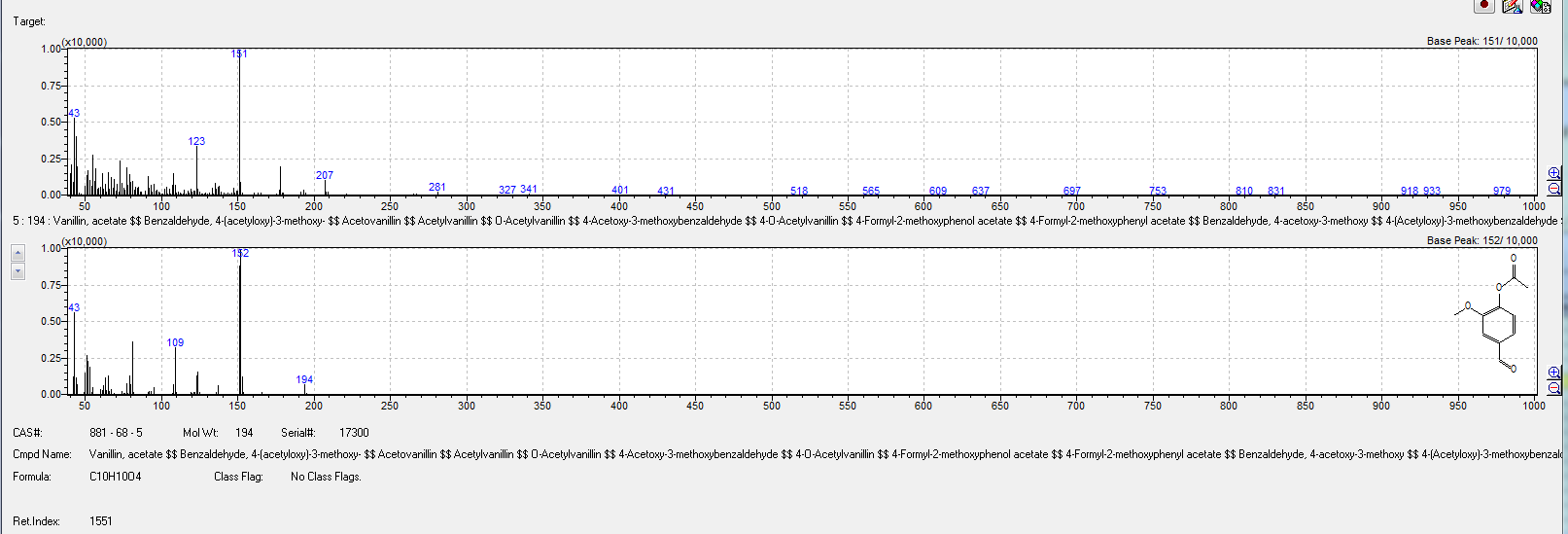 |
| Guanosine | 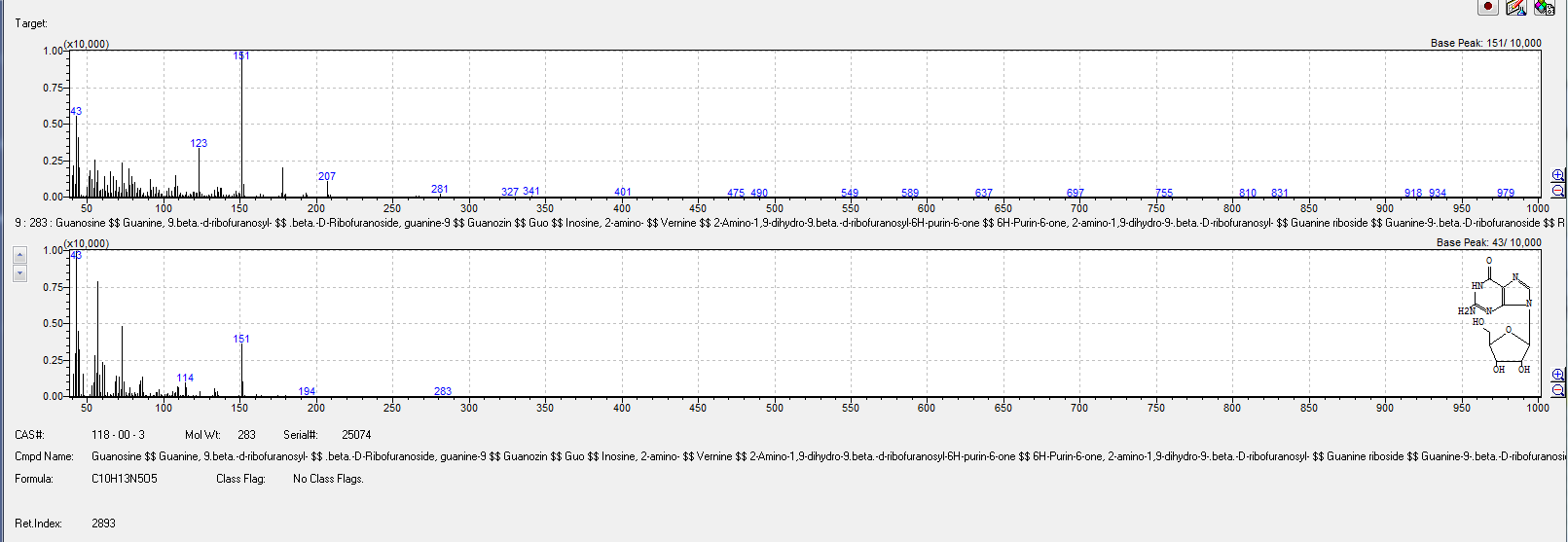 |
| trans-11-Tetradecenyl acetate | 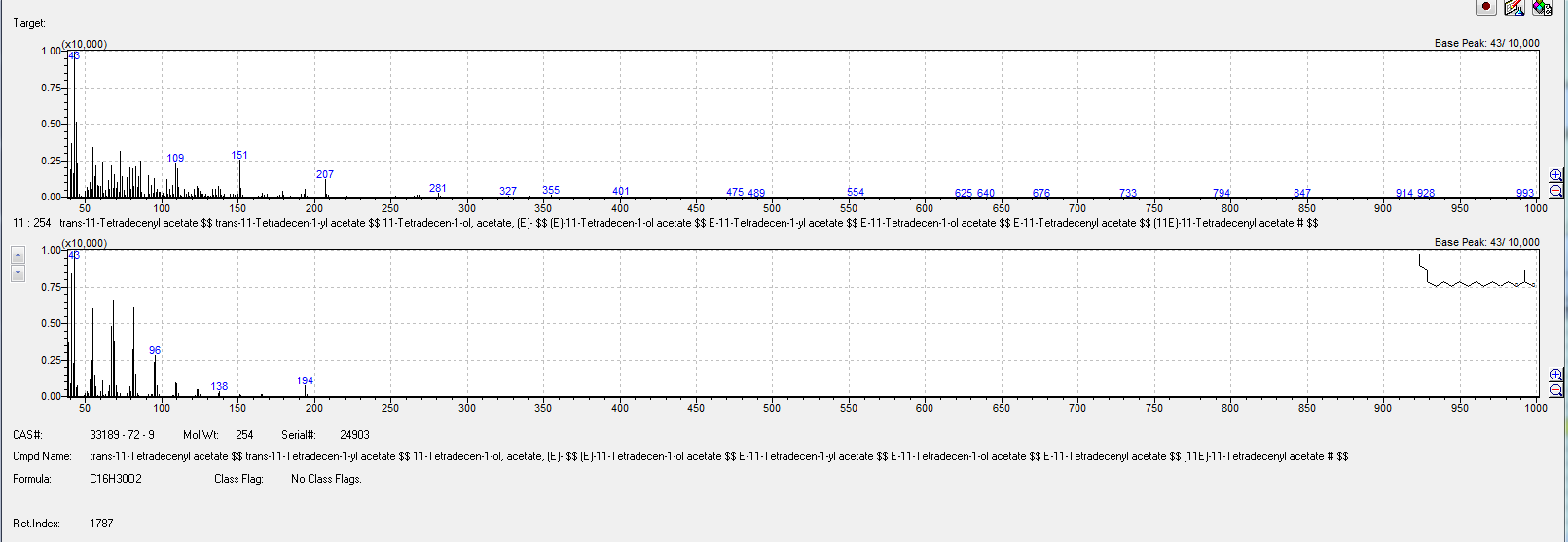 |
| D-Galactonic acid, gamma-lactone | 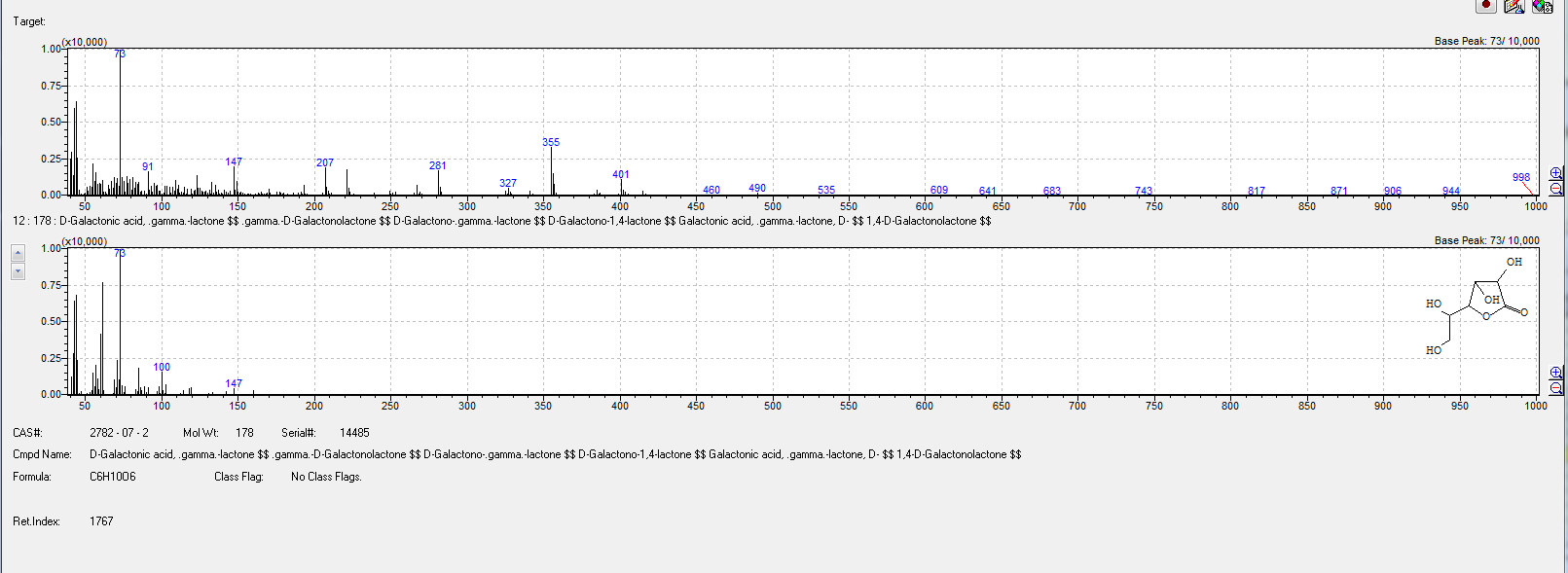 |
| Isopullegol | 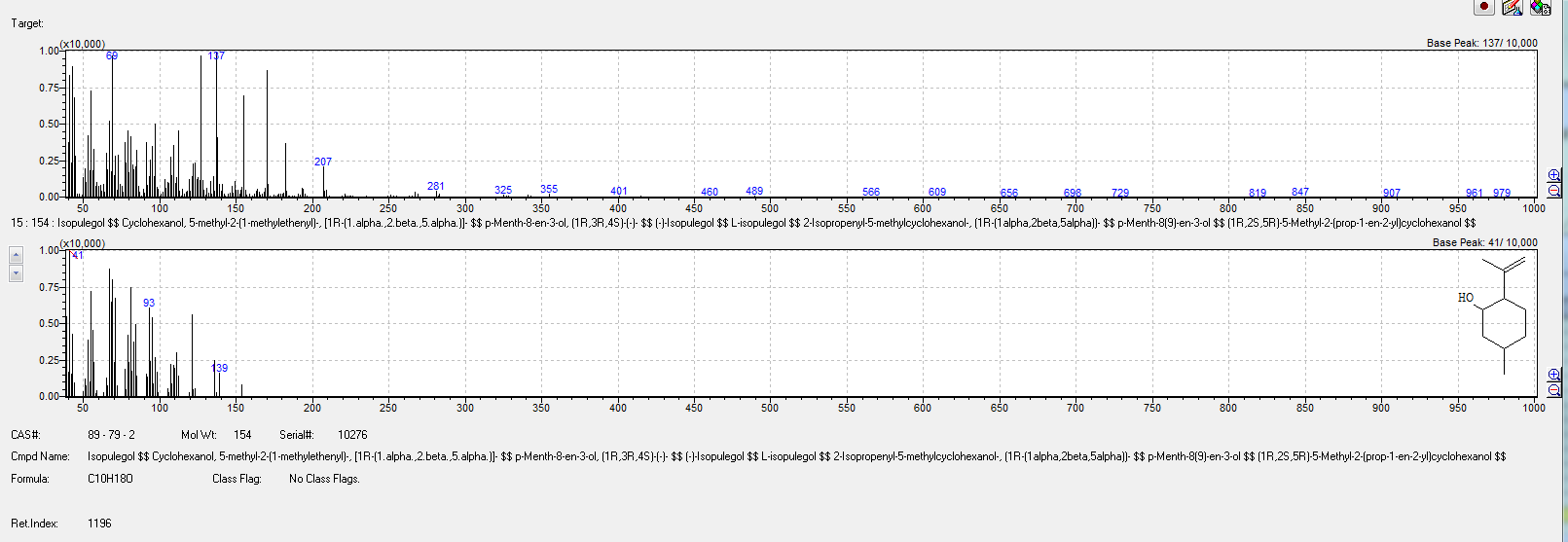 |
| Benzaldehyde | 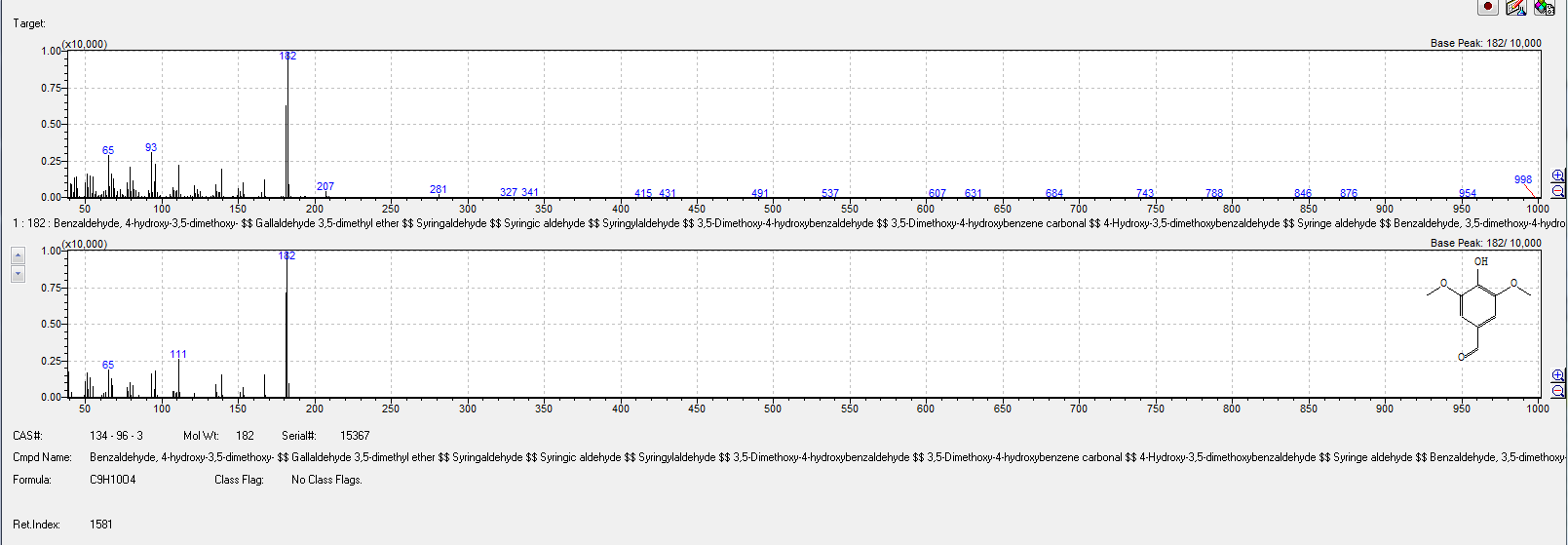 |
| Nephthalene | 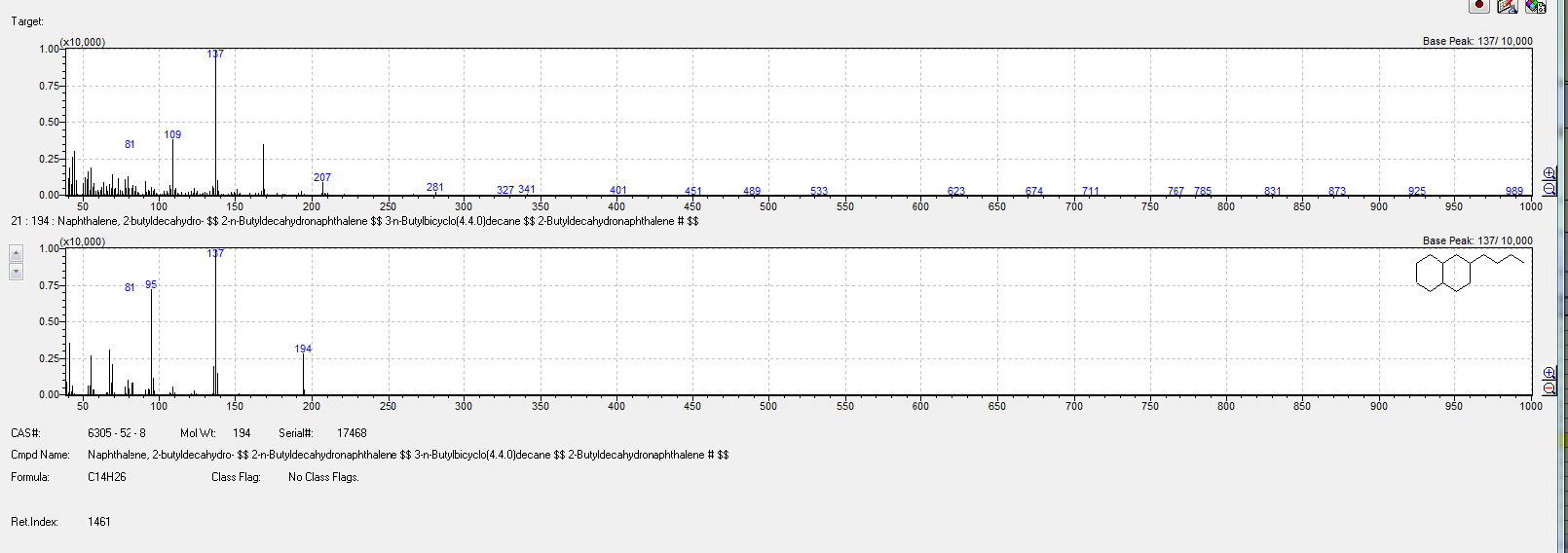 |
| 3-Buten-2-one, 3-methyl-4-(3,5,6-trimethyl-3,5-dimethoxy- | 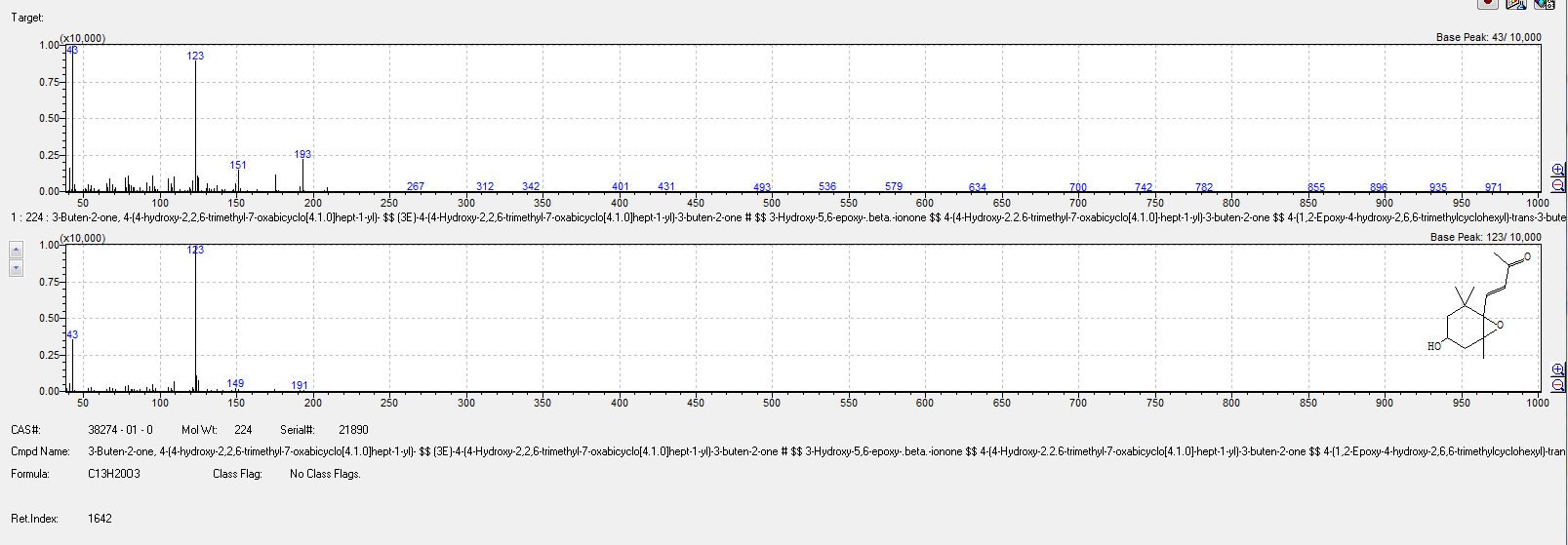 |
| Cis-p-Mentha2,8-dien-1-ol | 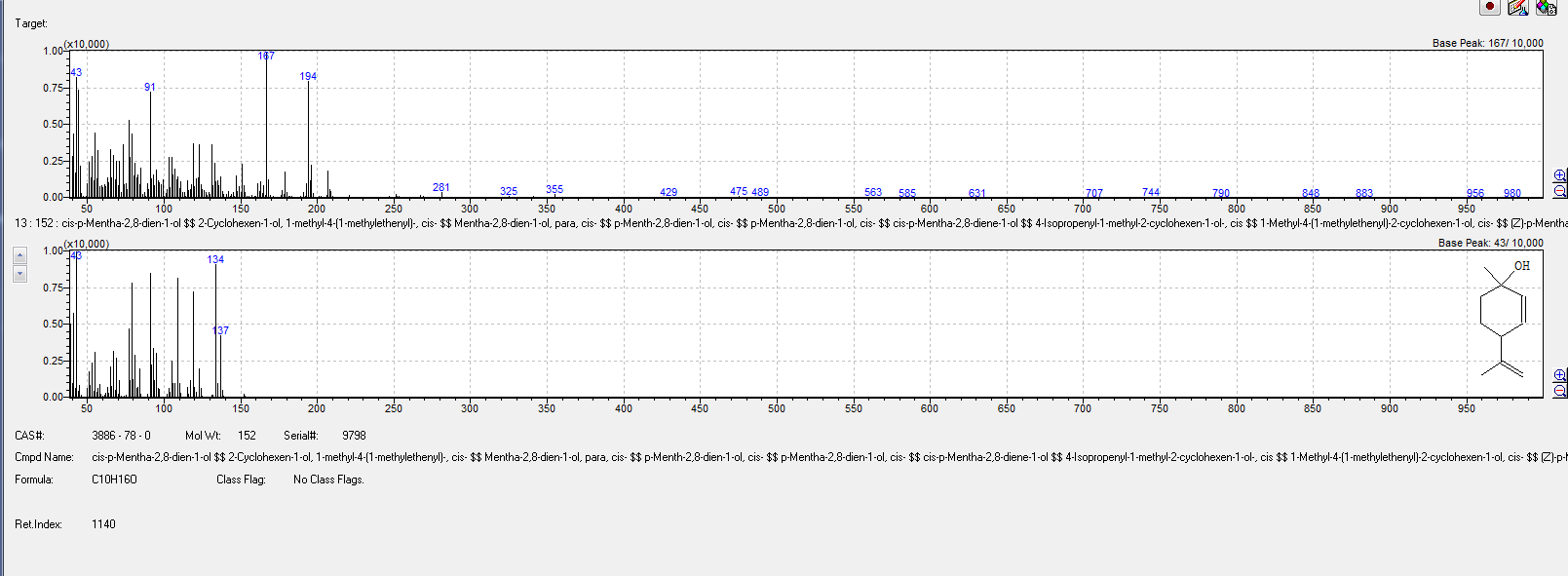 |
| trans Sesquisabinene hydrate | 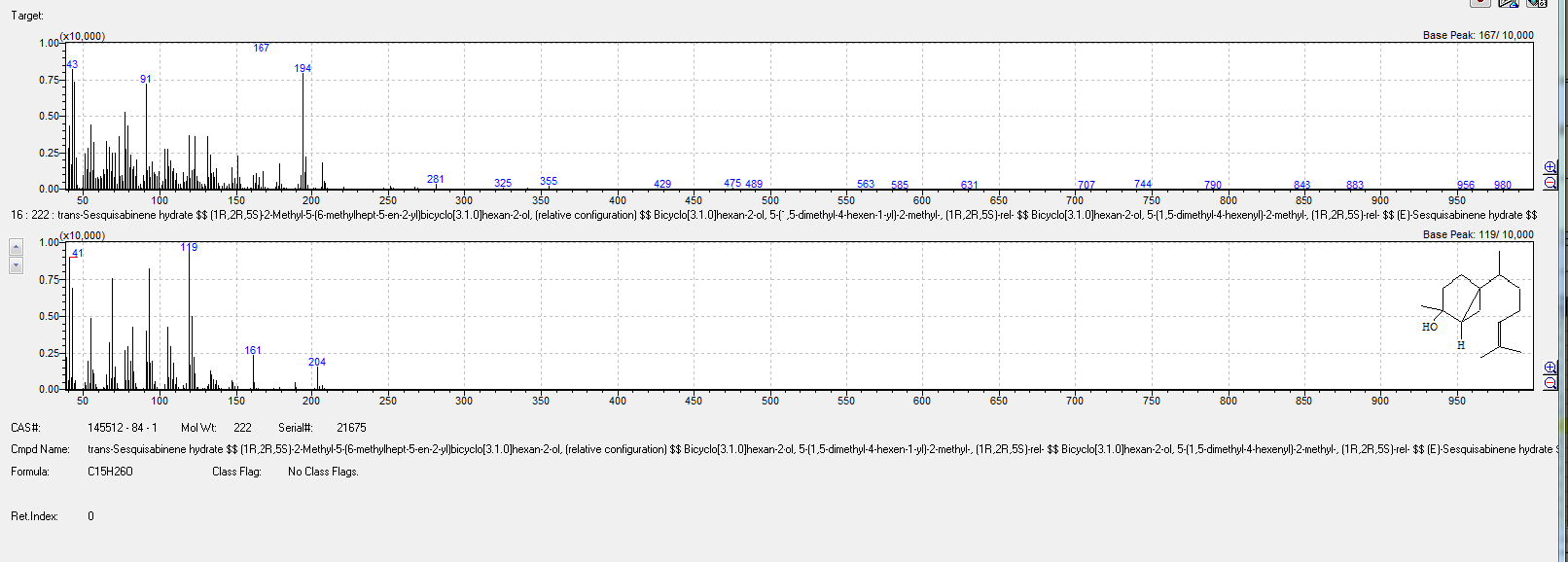 |
| 2-methoxy-6-methylaniline | 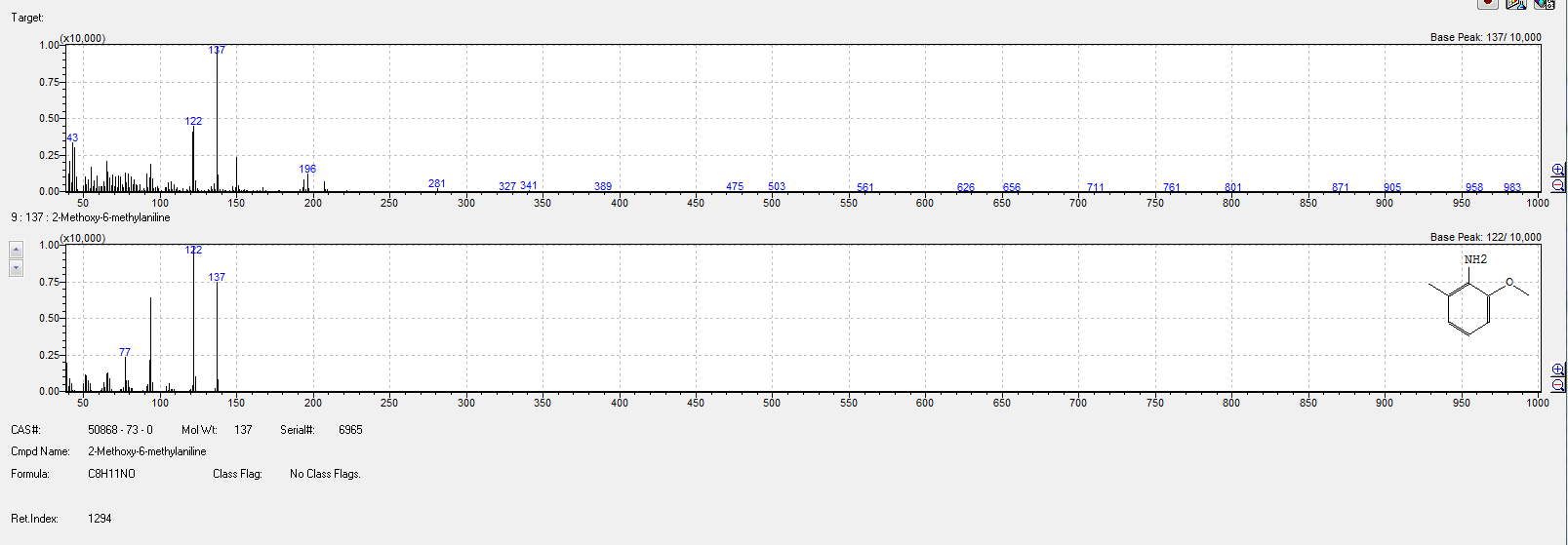 |
| Beta carotene | 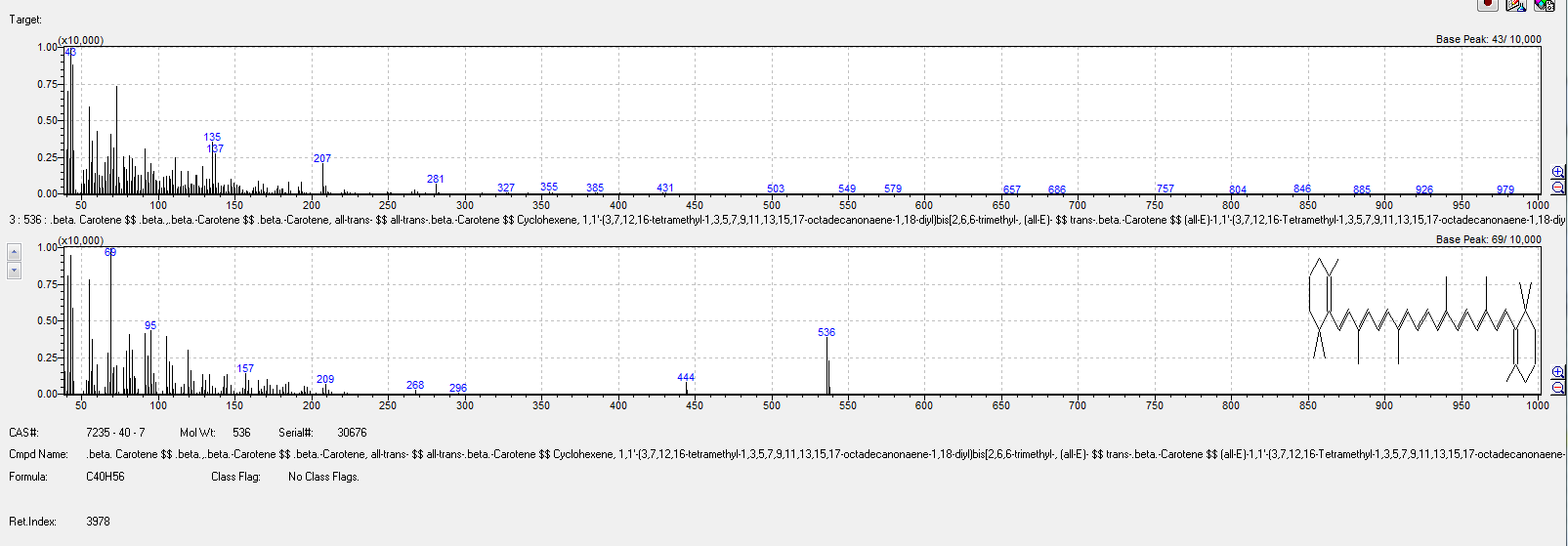 |
| Aprobarbital | 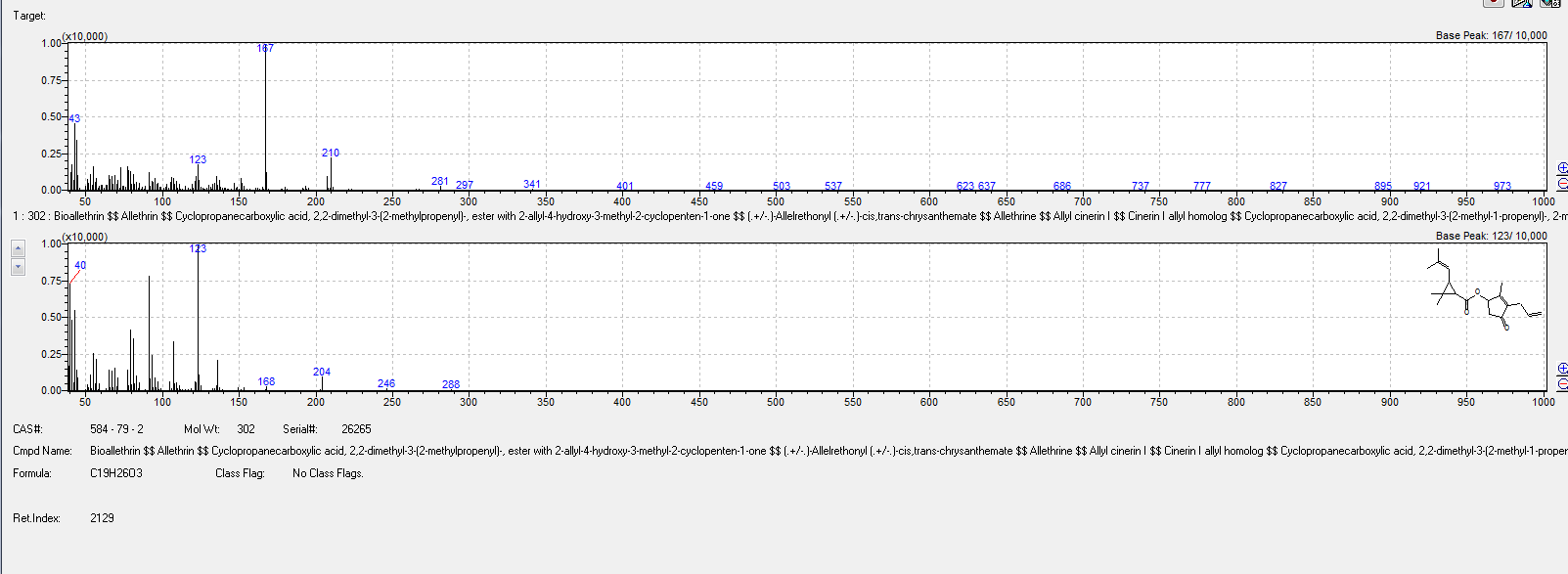 |
| Spiro[3,4]octan-5-one | 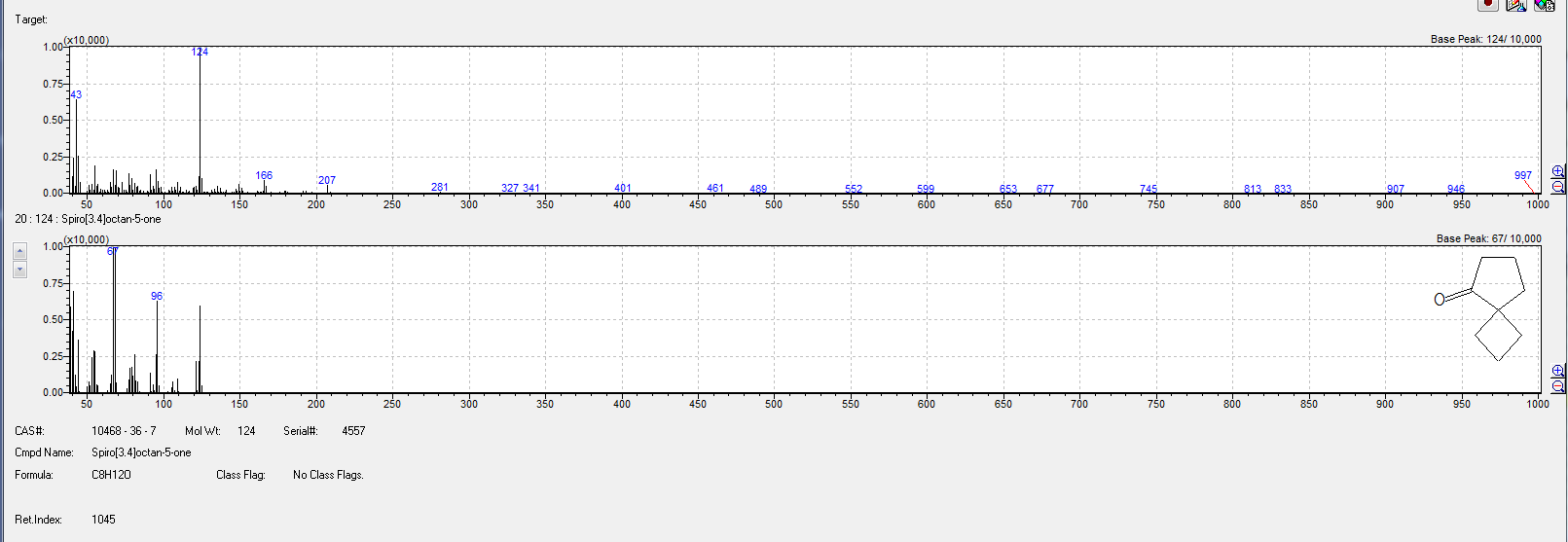 |
| 2-Dodecen-1-yi(-)succinic anhydride | 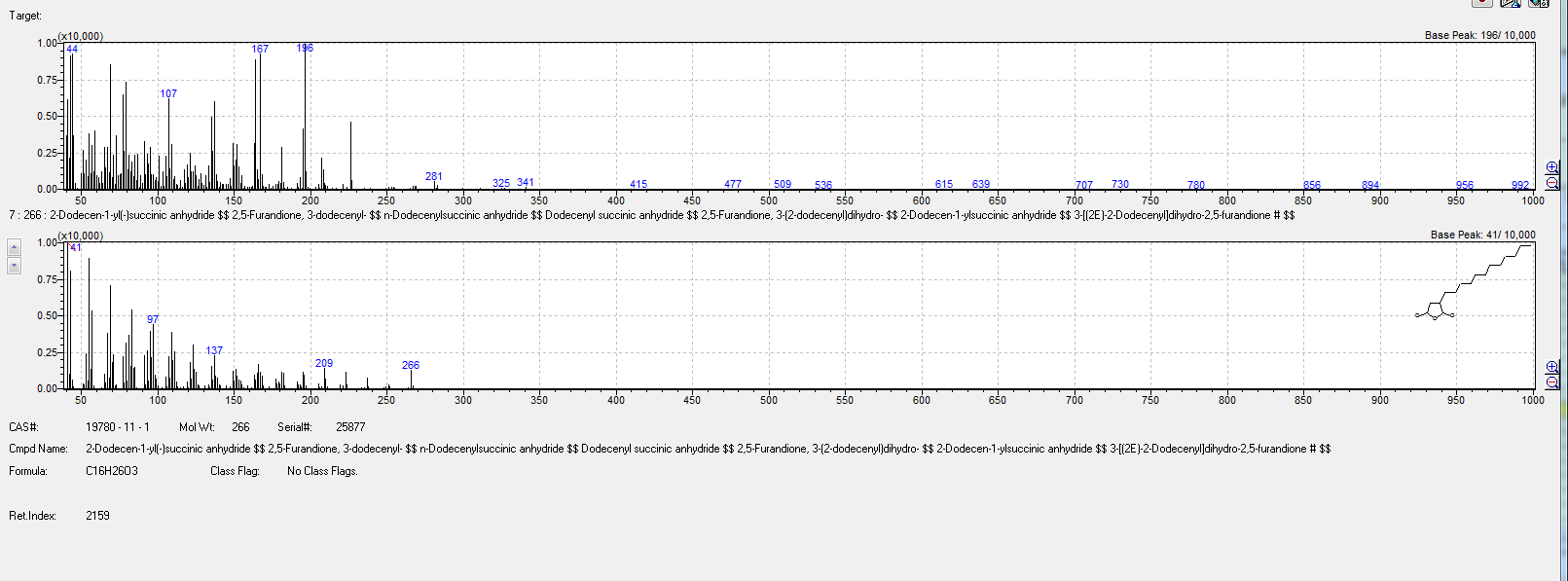 |
| Phytol | 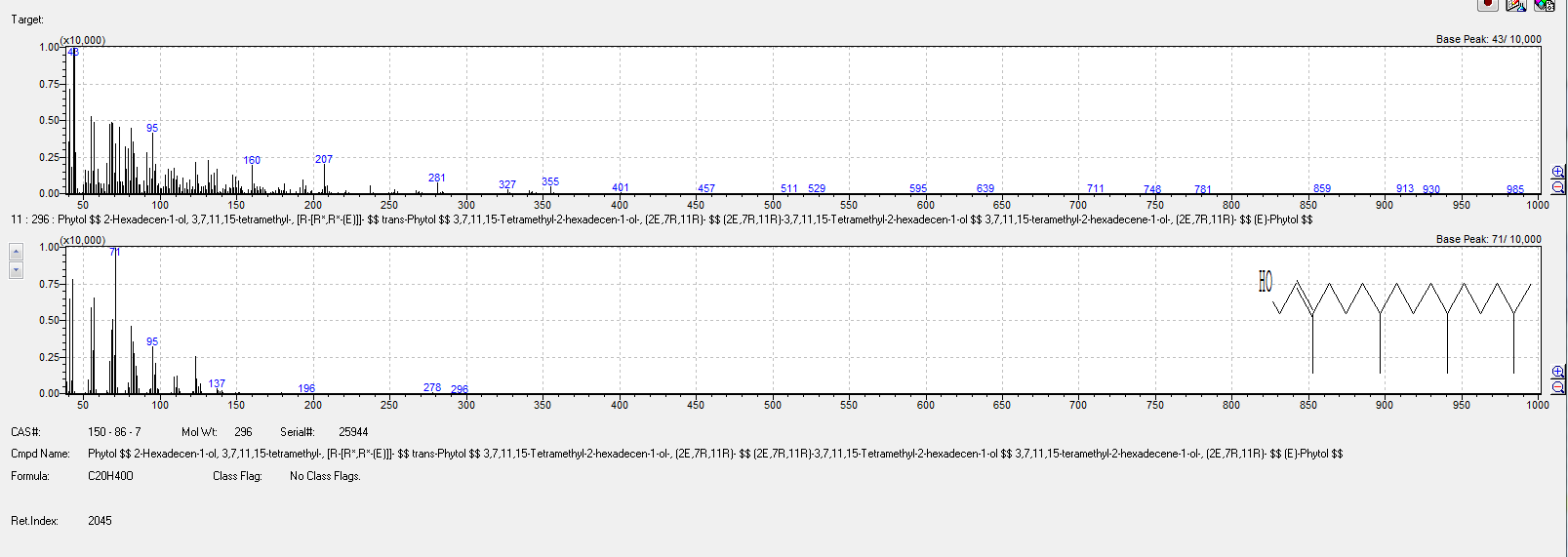 |
| Digitoxin | 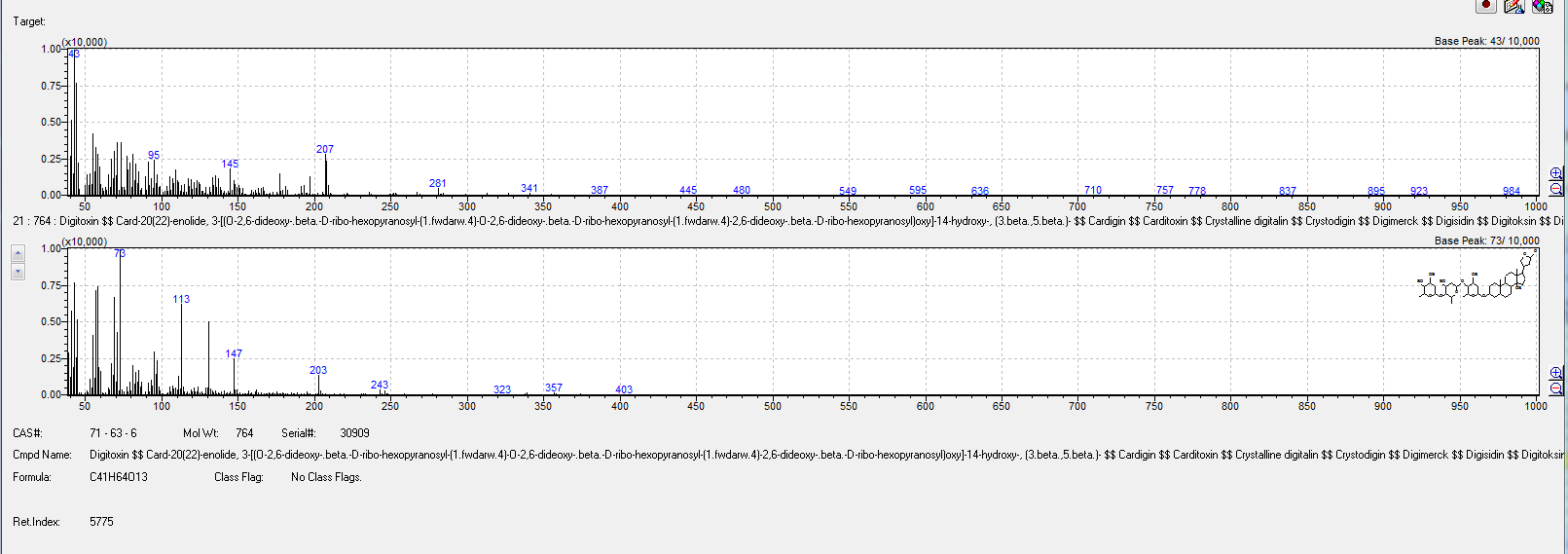 |
| Crysanthemic acid | 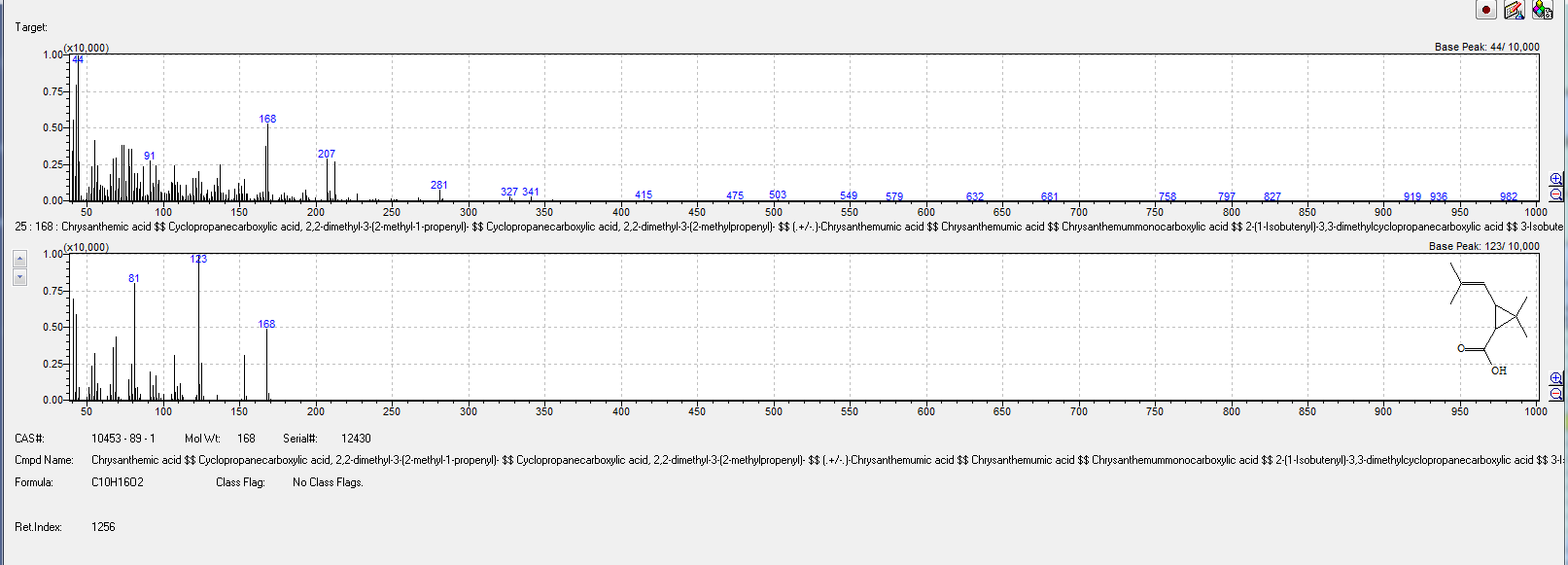 |
| n-Hexadecanoic acid | 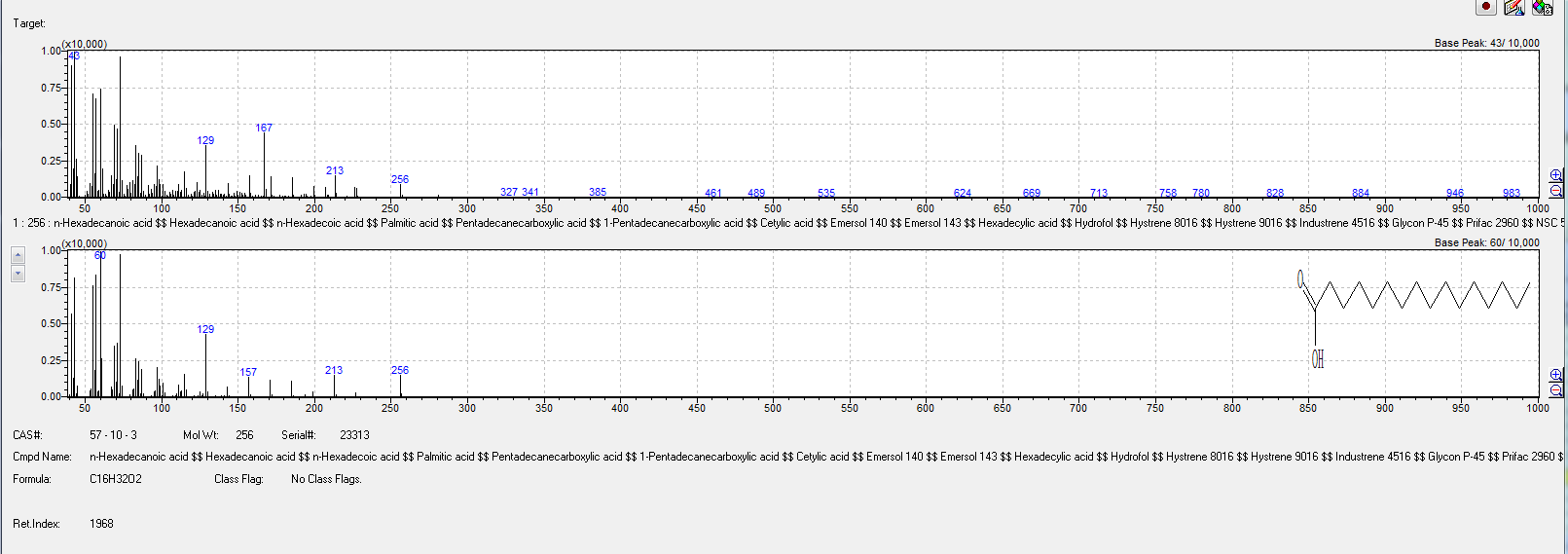 |
| Beta asarone | 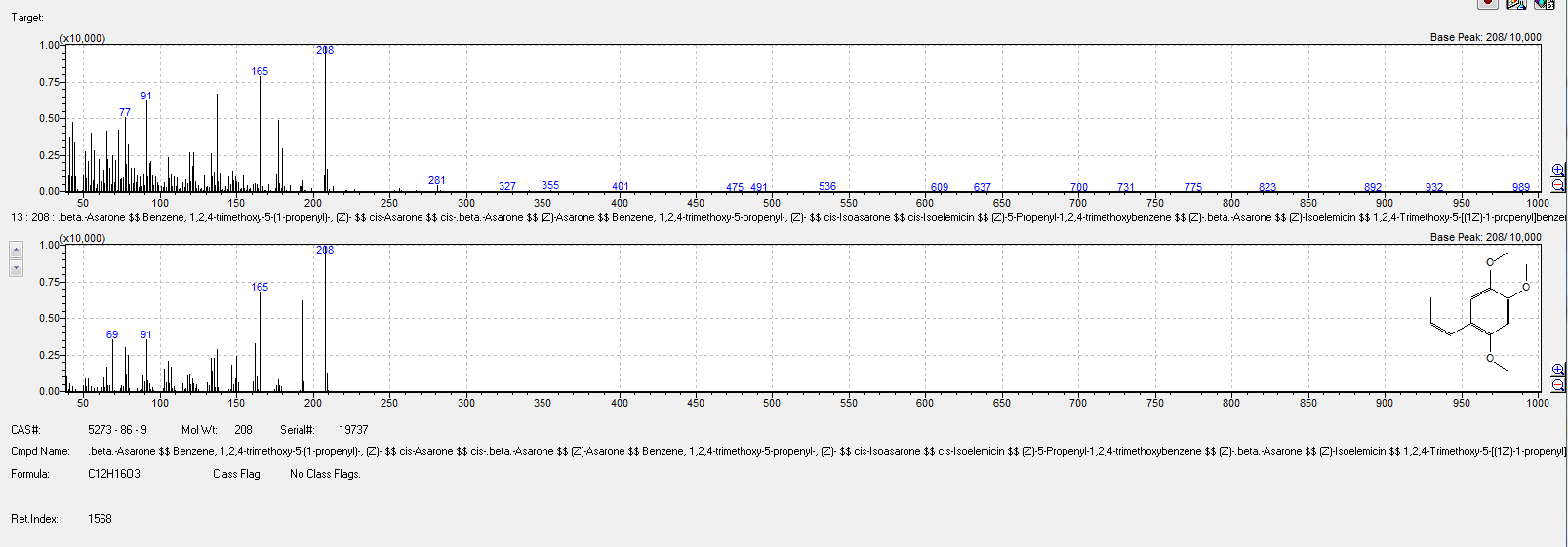 |
| Benzene propanoic acid, 2,3-dihydroxypropyl ester | 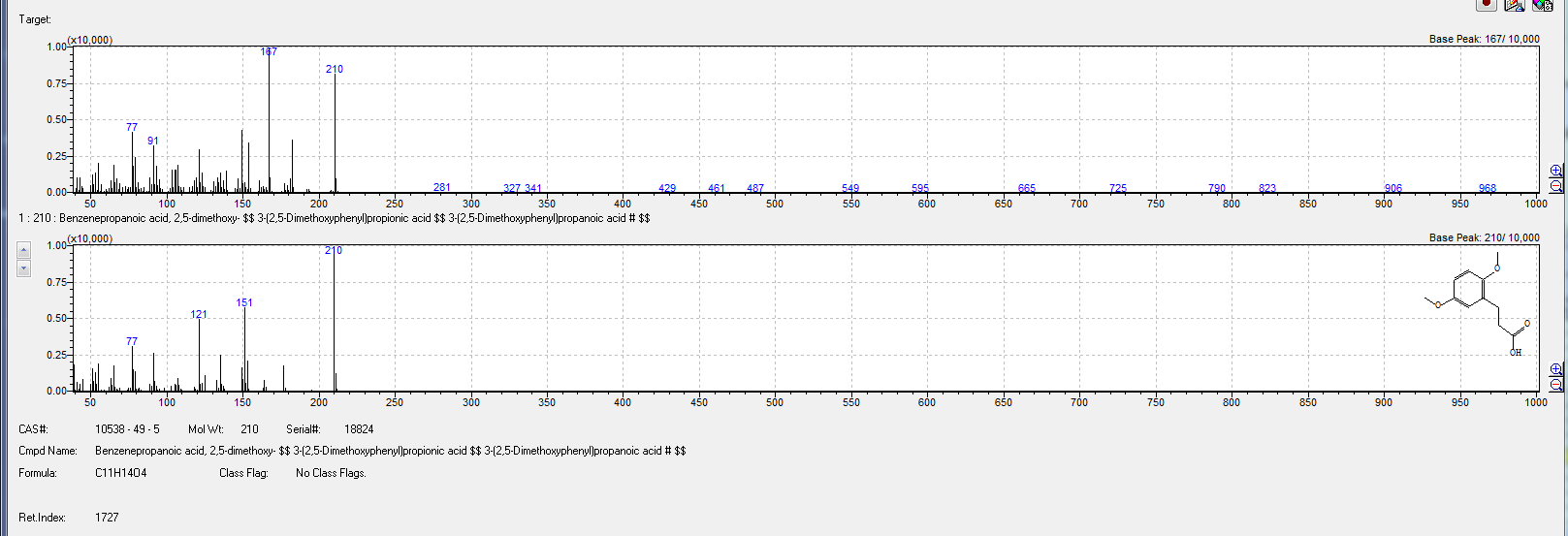 |
| Decanoic acid, 2,3-dihydroxypropyl ester | 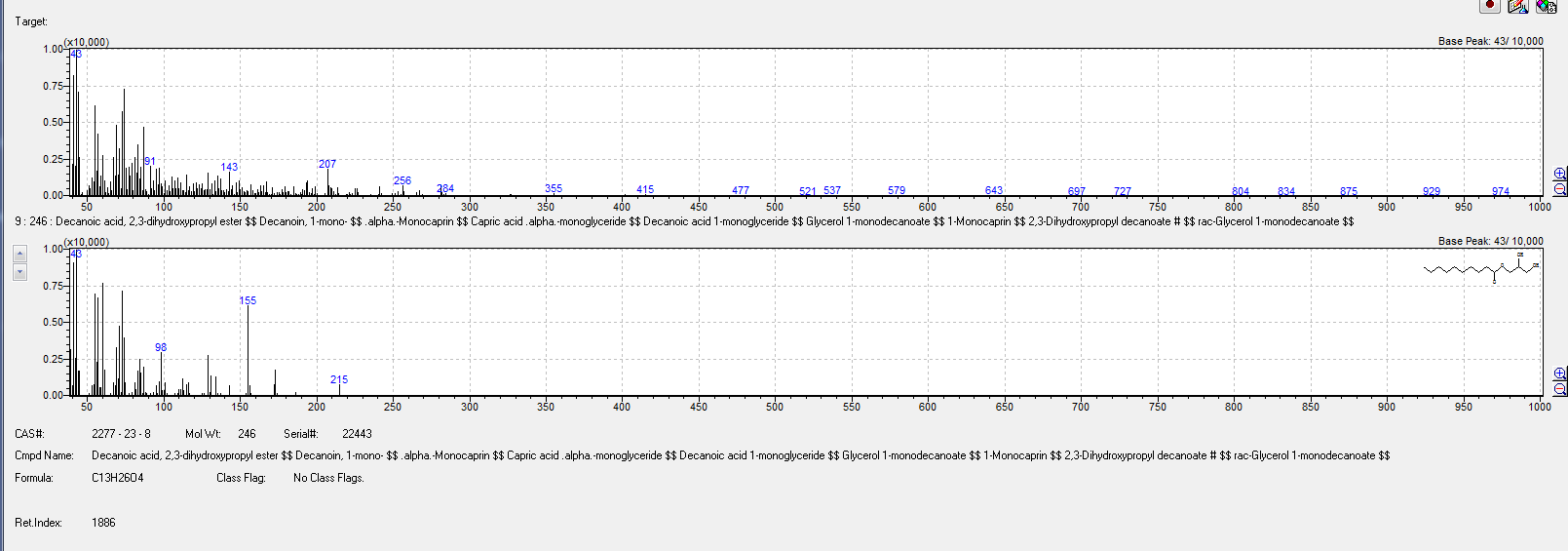 |
| 9,12- Octadecanoic acid, methyl ester | 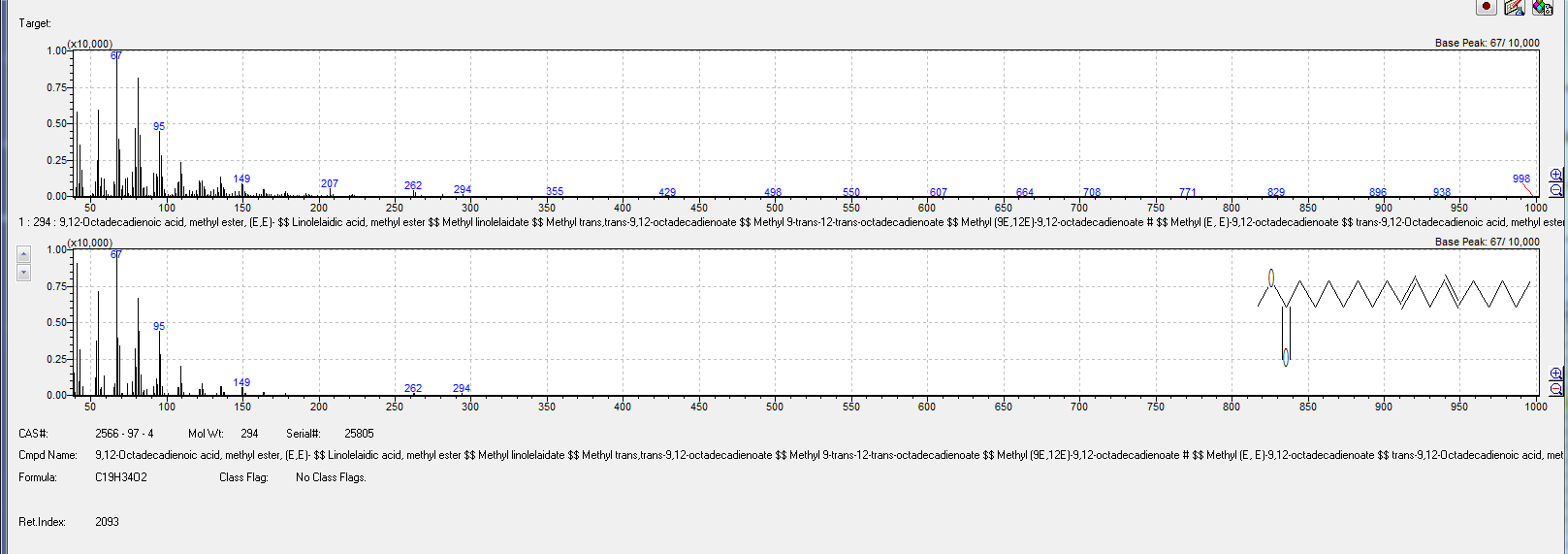 |
| 7-Hexadecenoic acid, methyl ester | 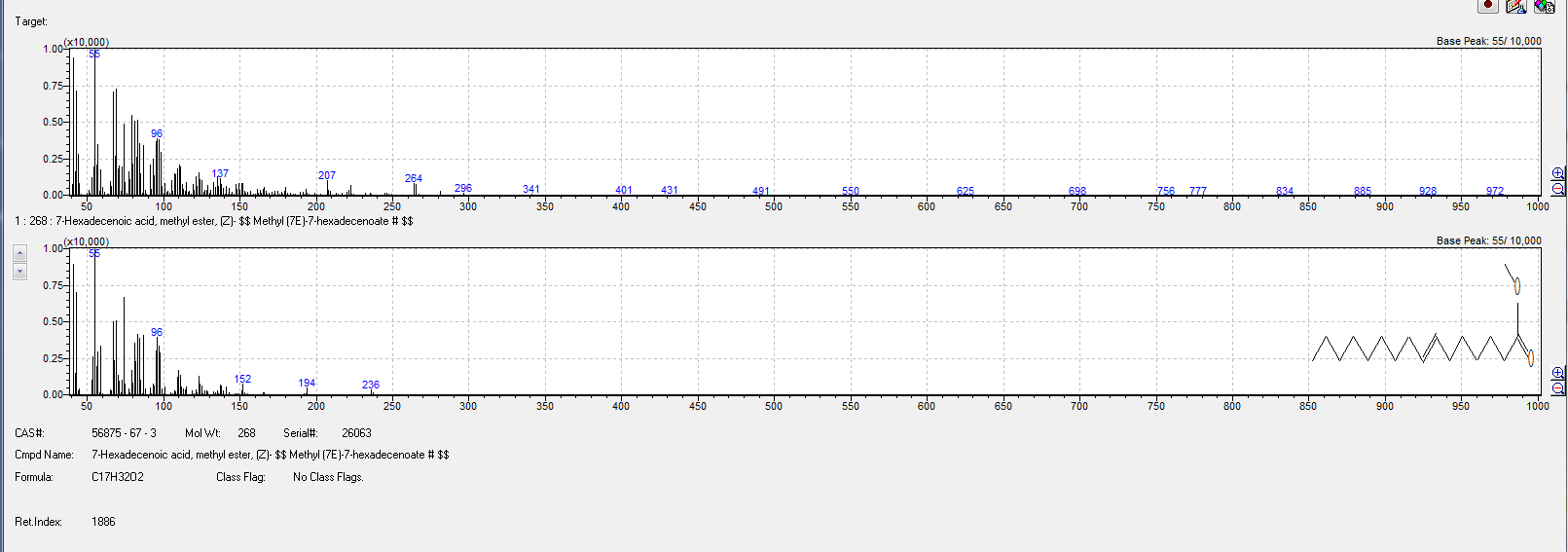 |
| Citronellol | 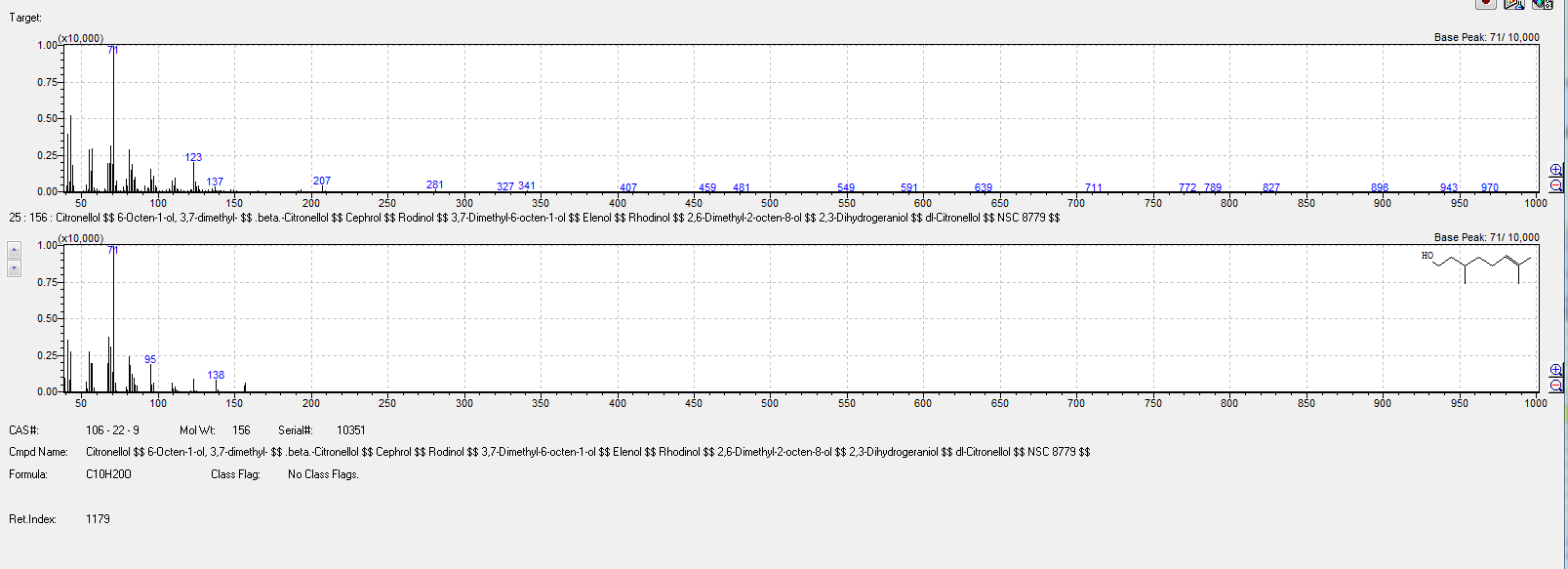 |
| Undec-10-ynoic acid | 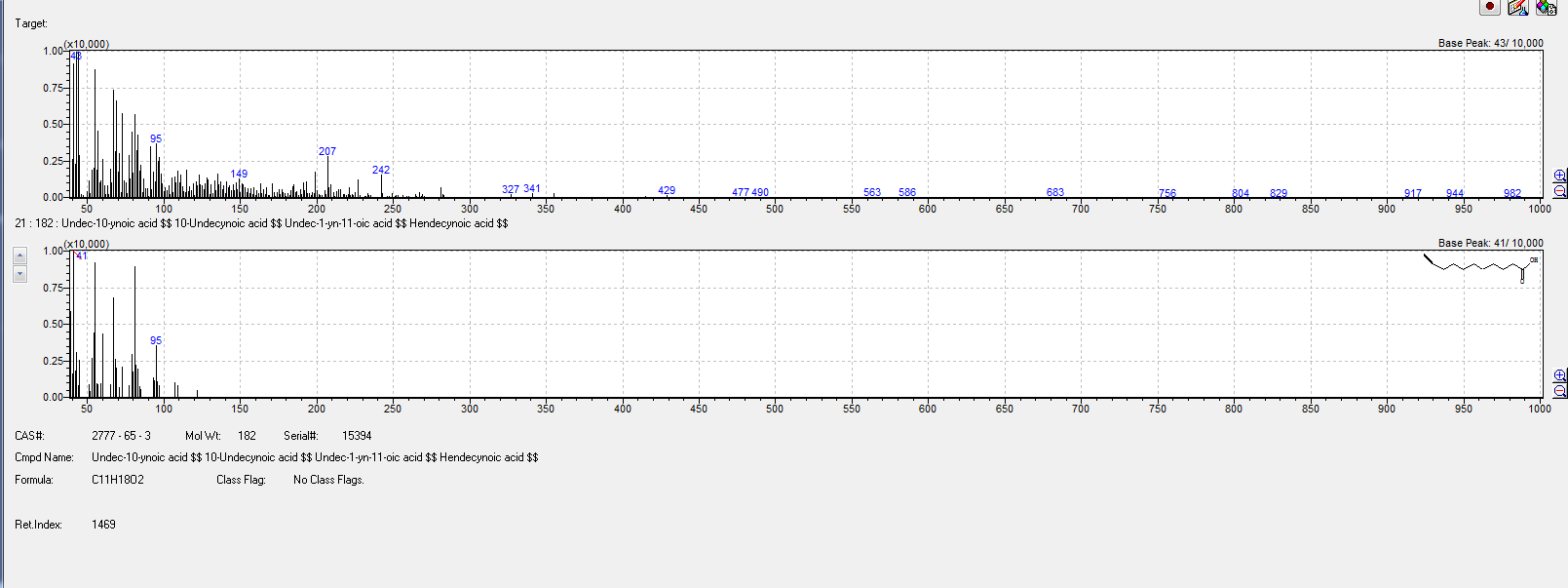 |
| 6-octadecenoic acid, methyl ester | 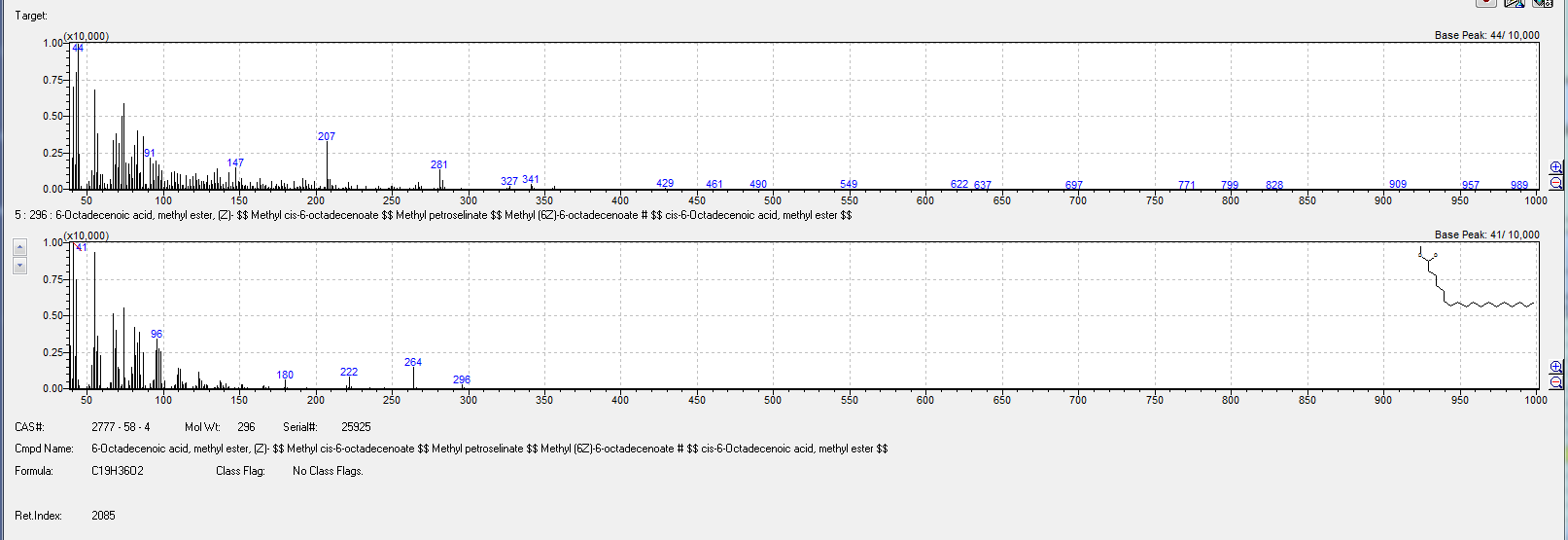 |
| Dodecanal | 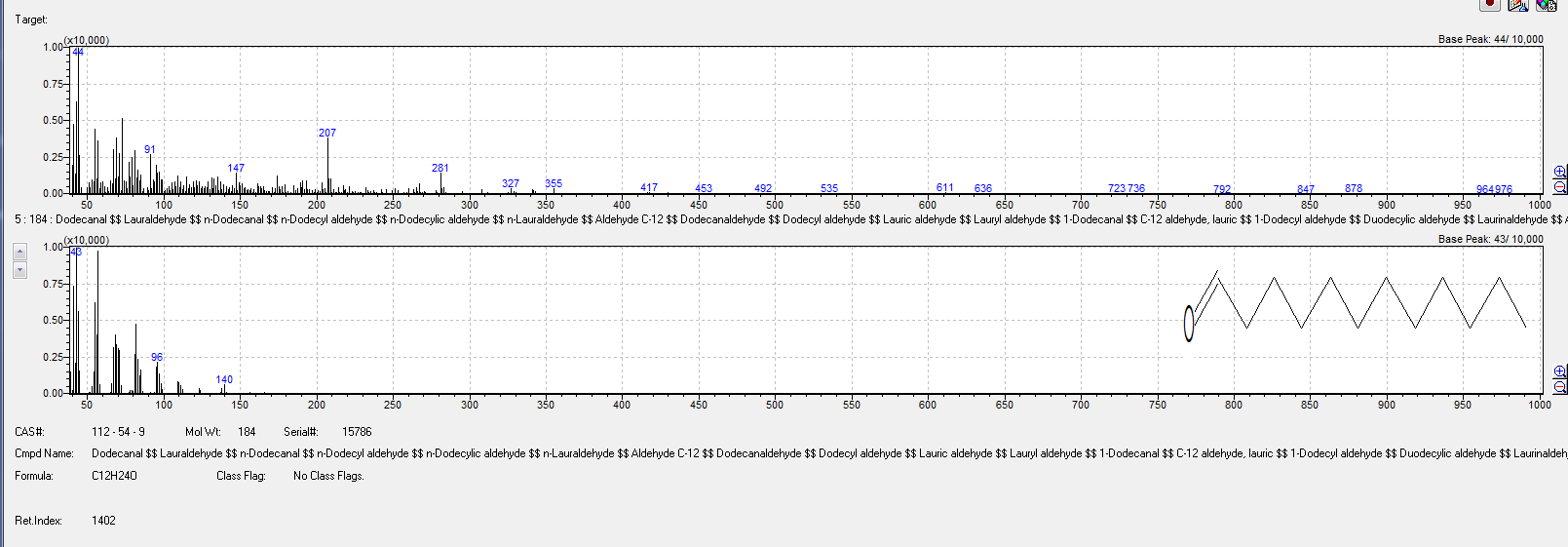 |
| Nerolidol | 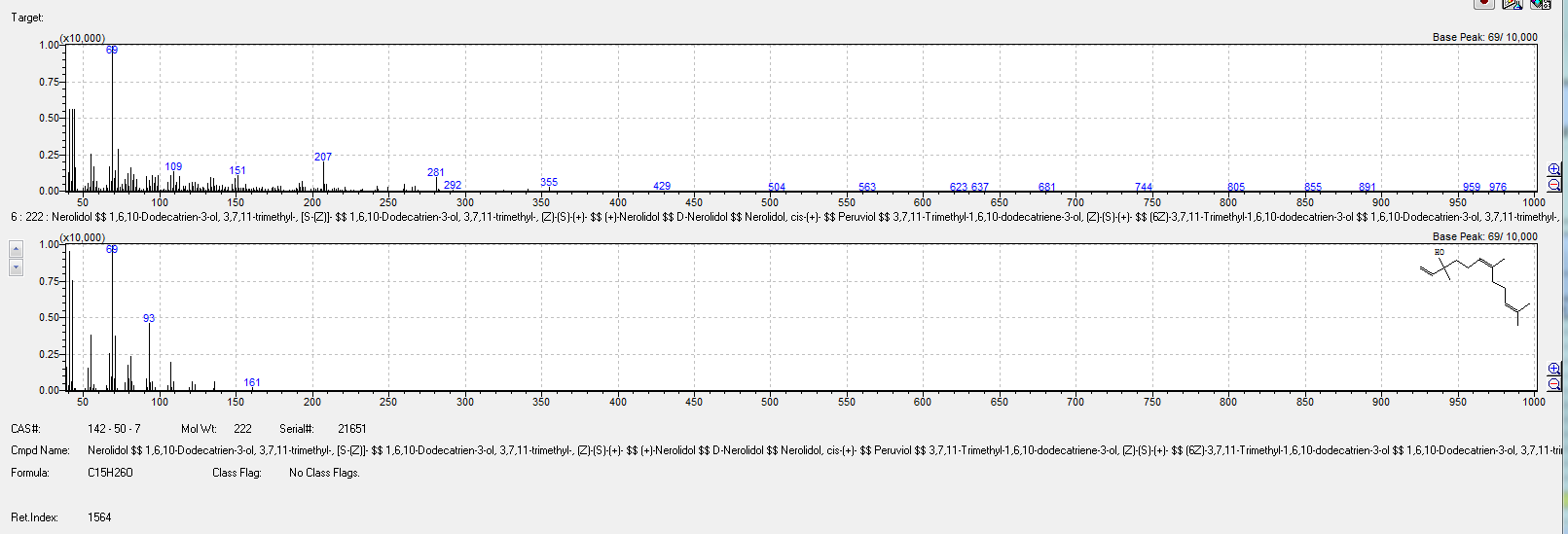 |
| Cyclohexane, eicosyl- | 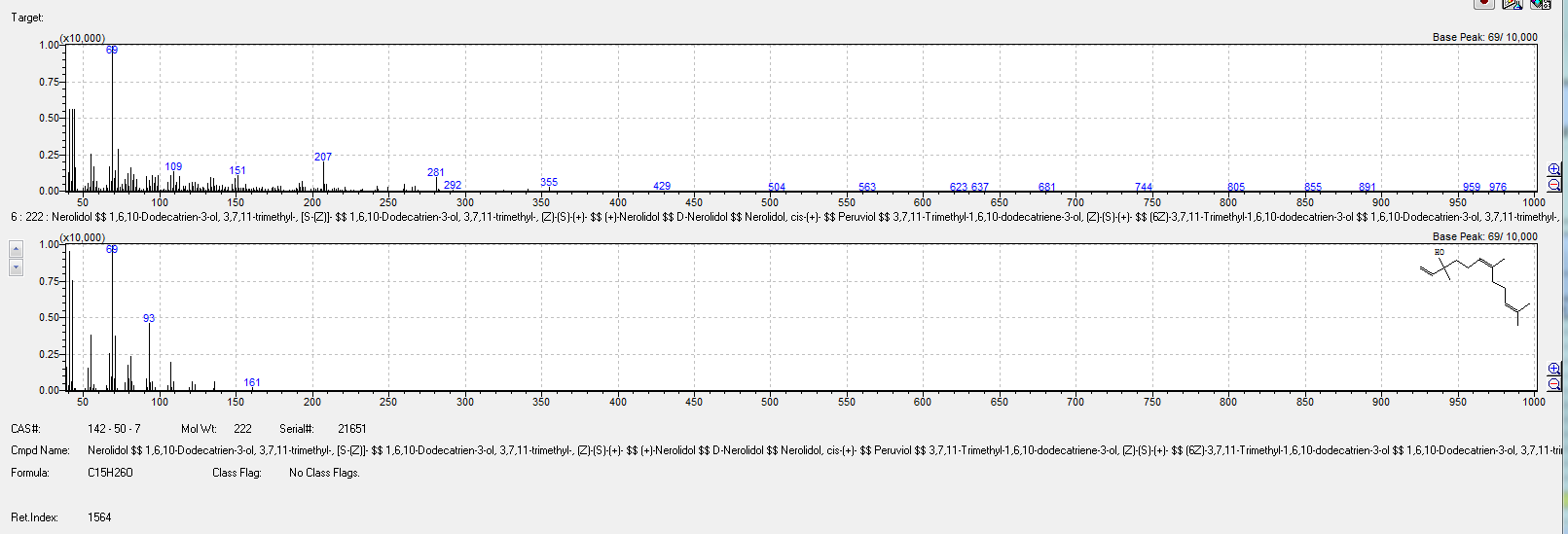 |
| Glyserol 1-palmitate | 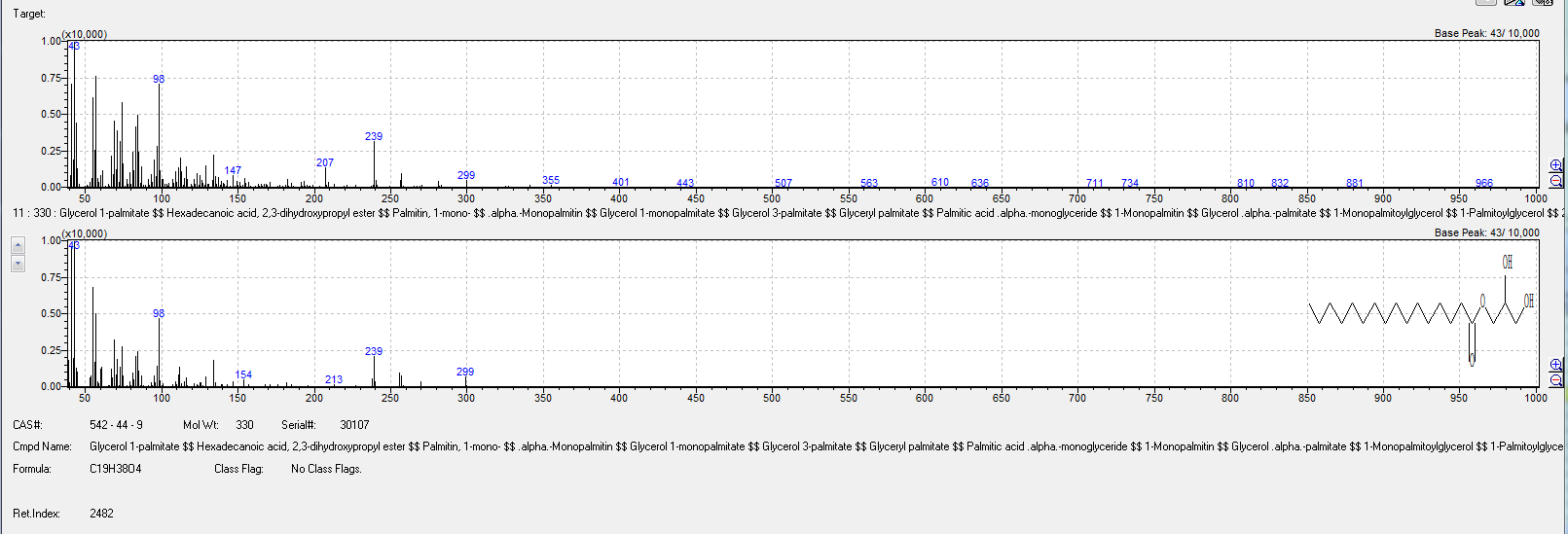 |
| Hexadecanal | 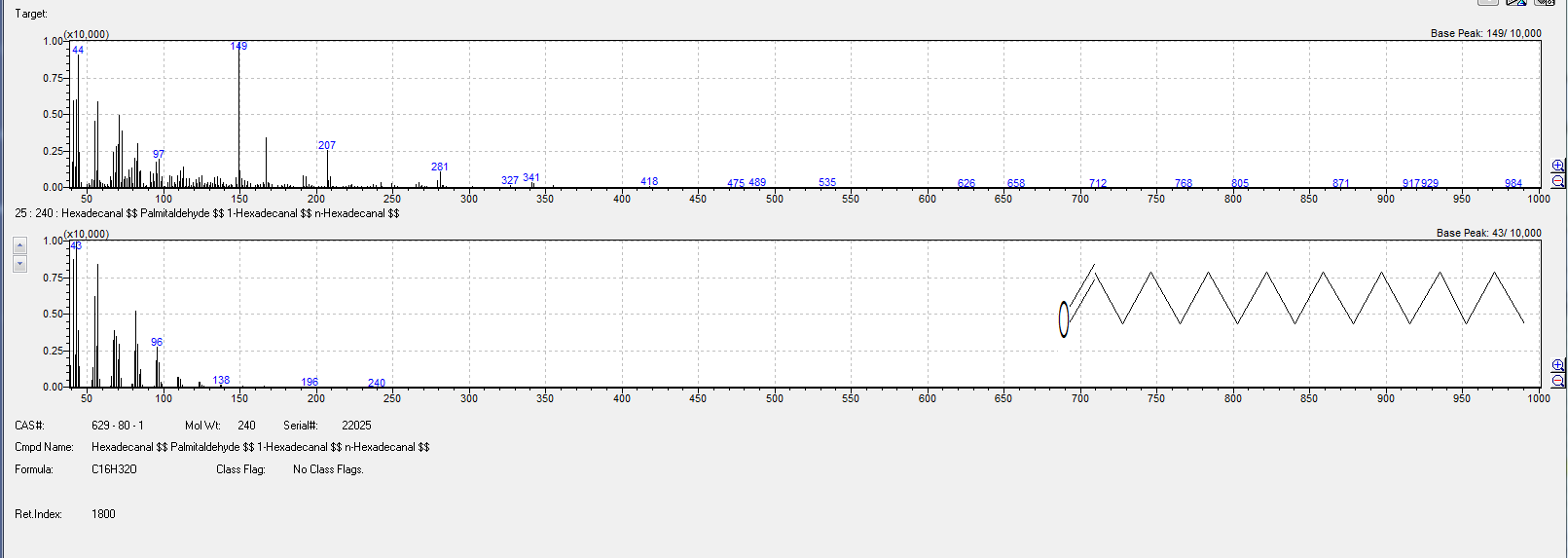 |
| Meprobamate | 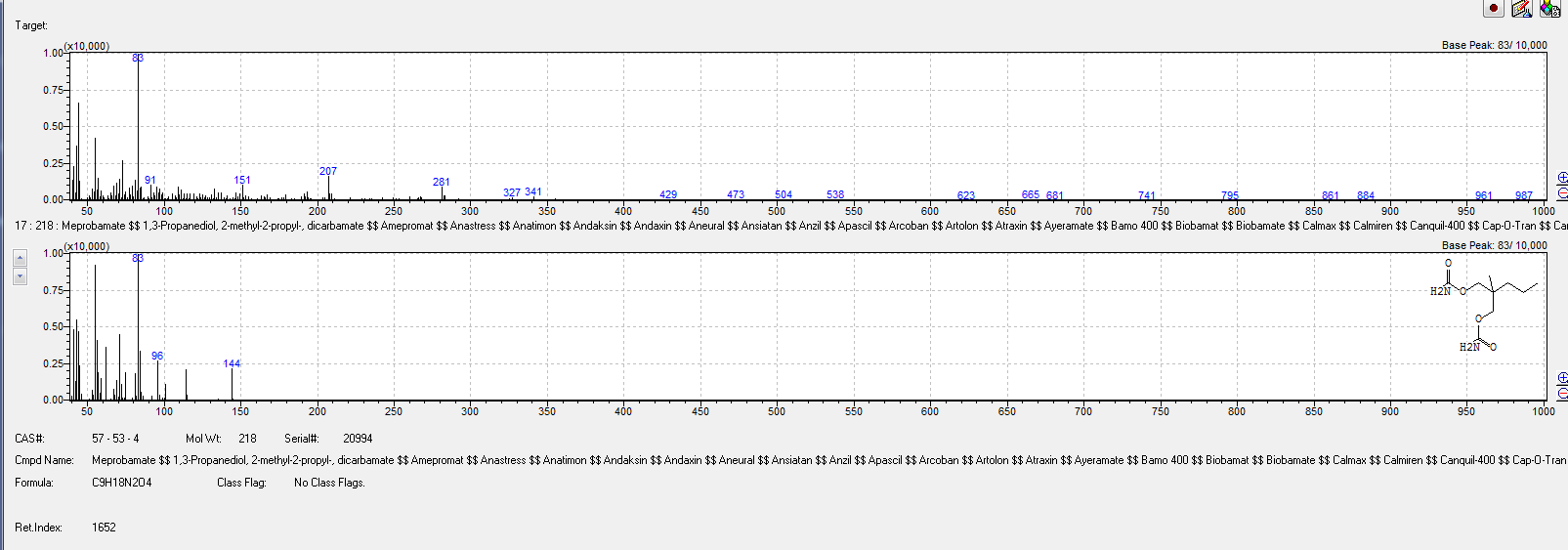 |
| Daucol | 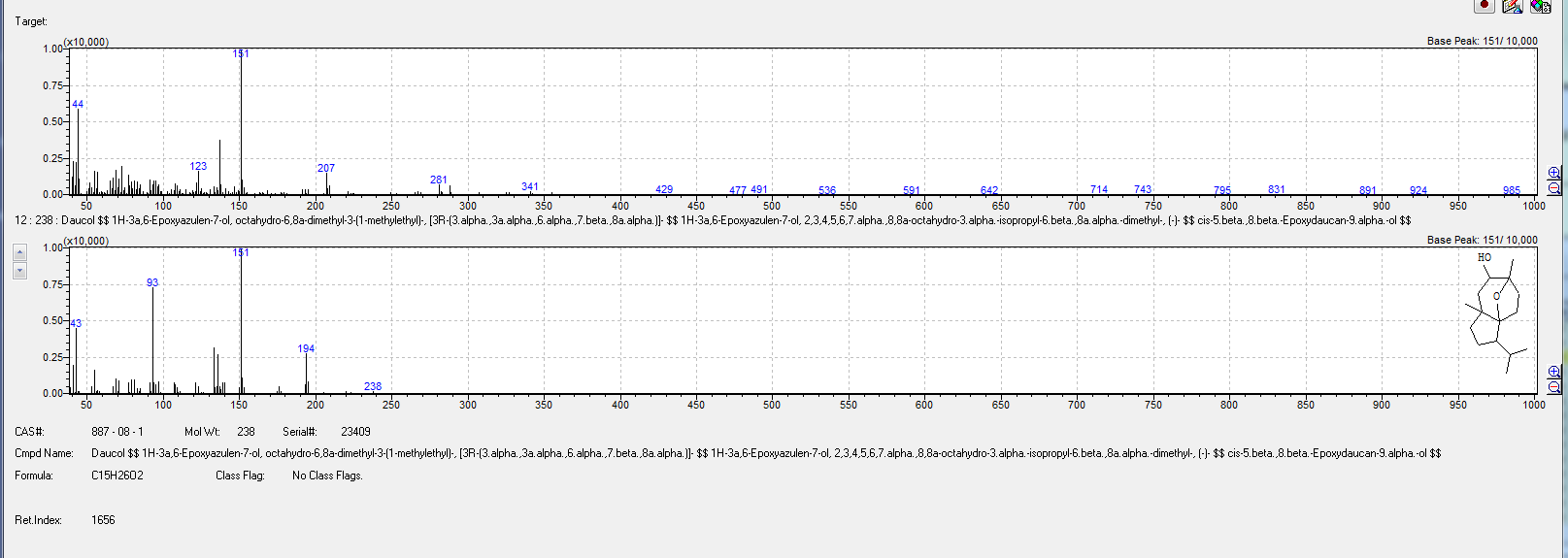 |
| Methotrexate | 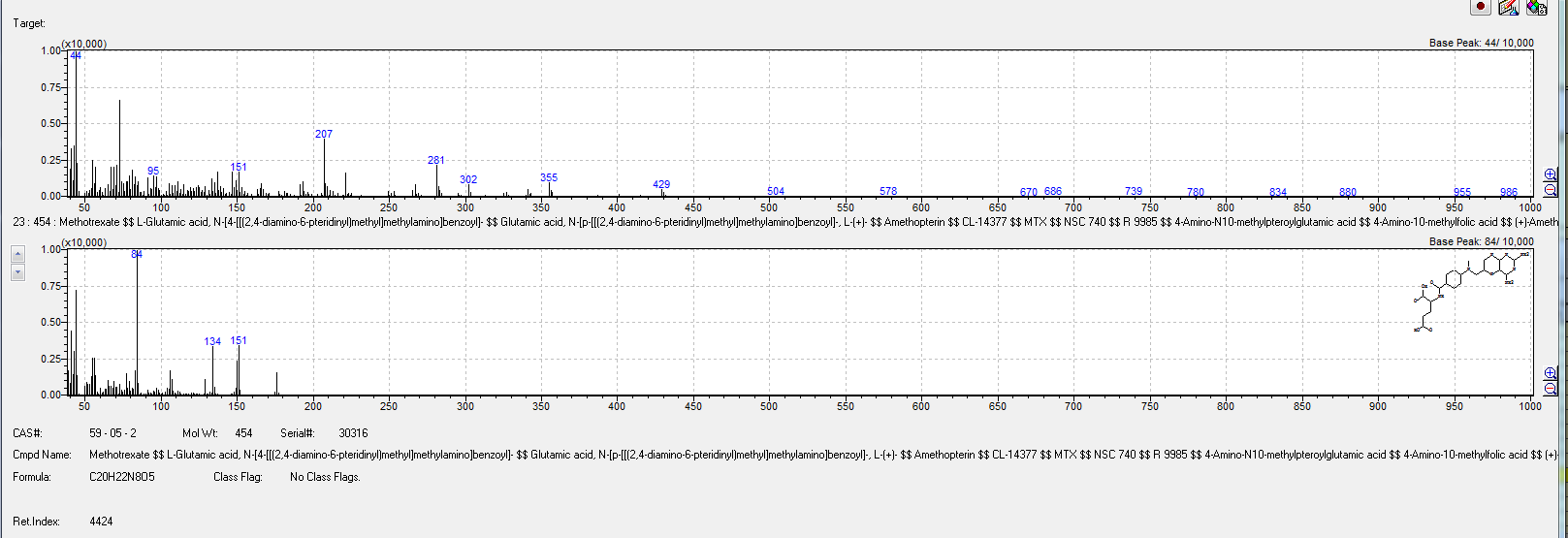 |
| Estradiol | 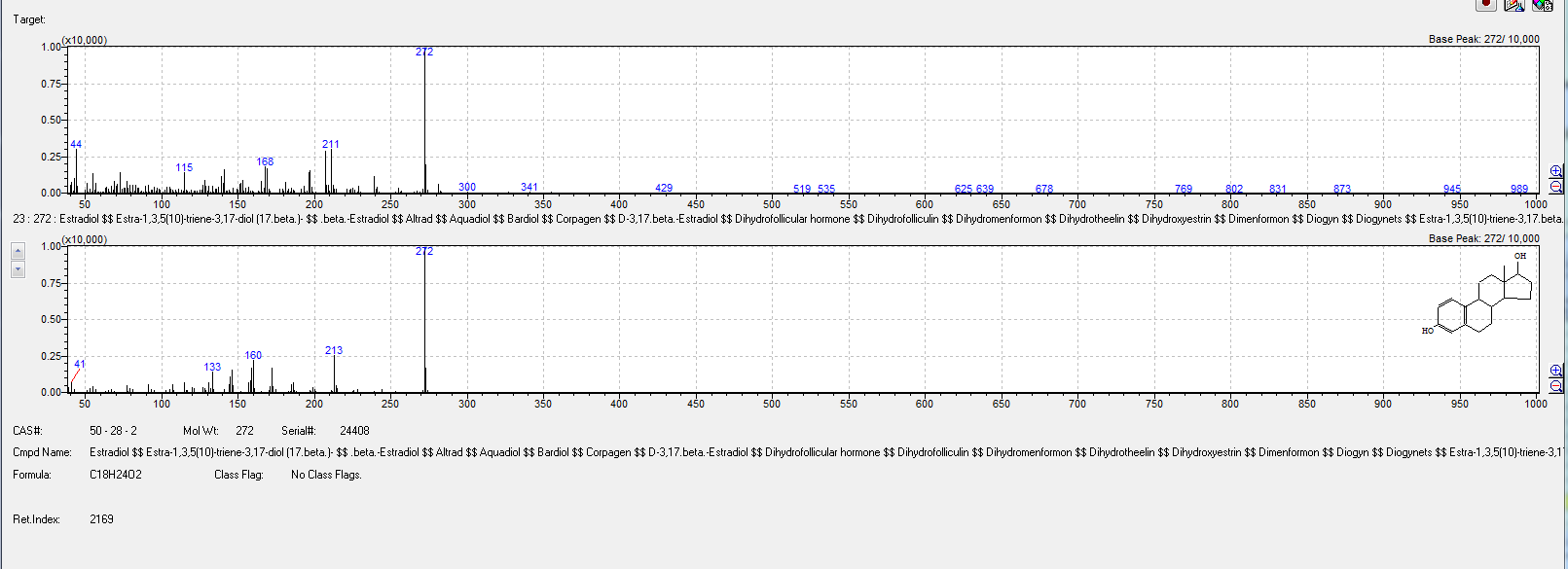 |
| Octadecanoic acid, 2-hydroxy-1,3-propanediyl | 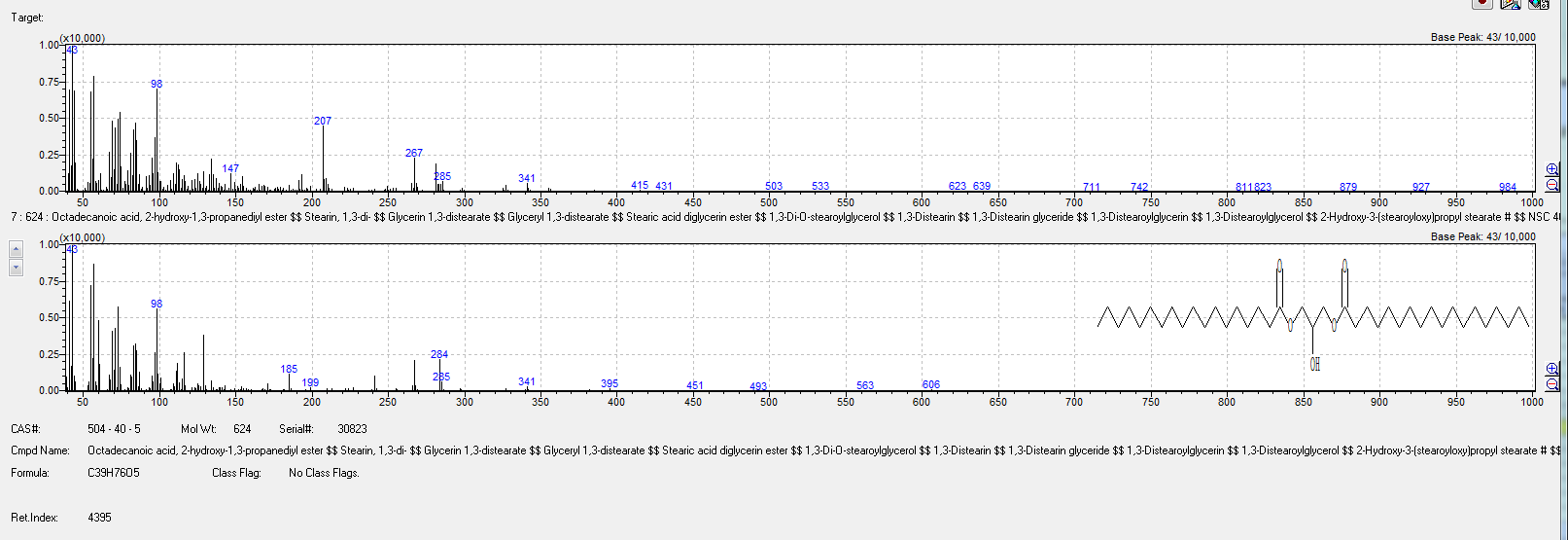 |
| Mebutamate | 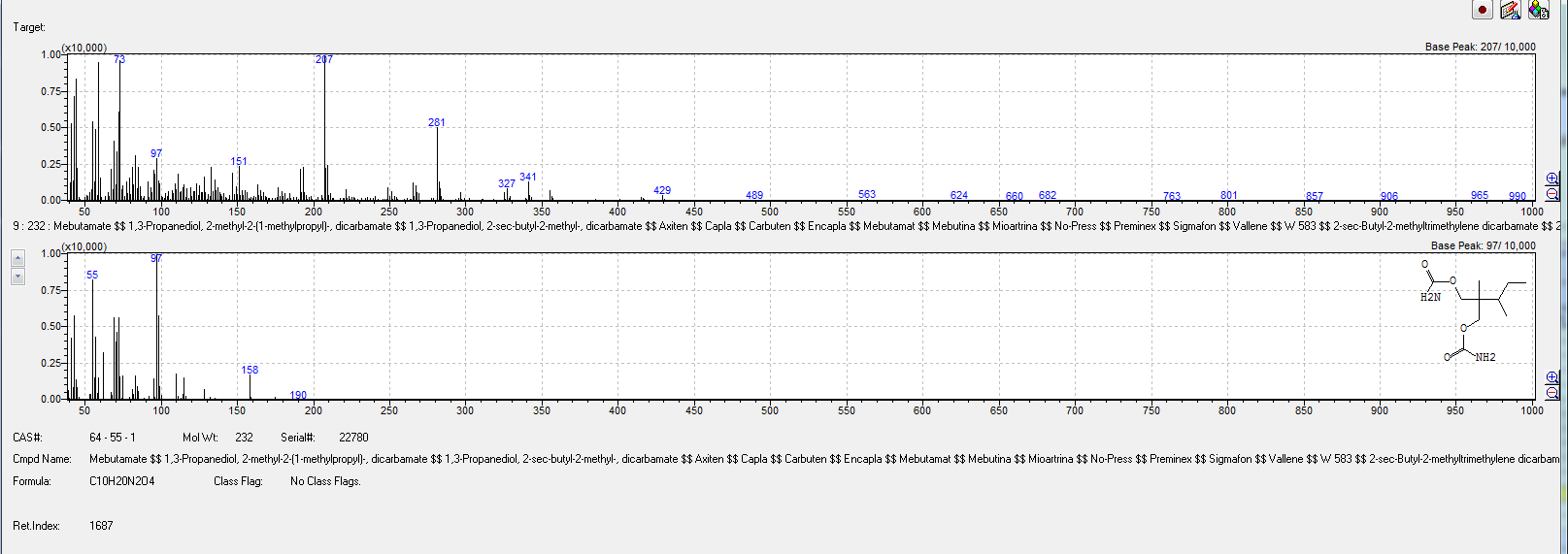 |
| Androsta-3,5-dien-3-ol, 17-acetyl-3-o-(t-butyl | 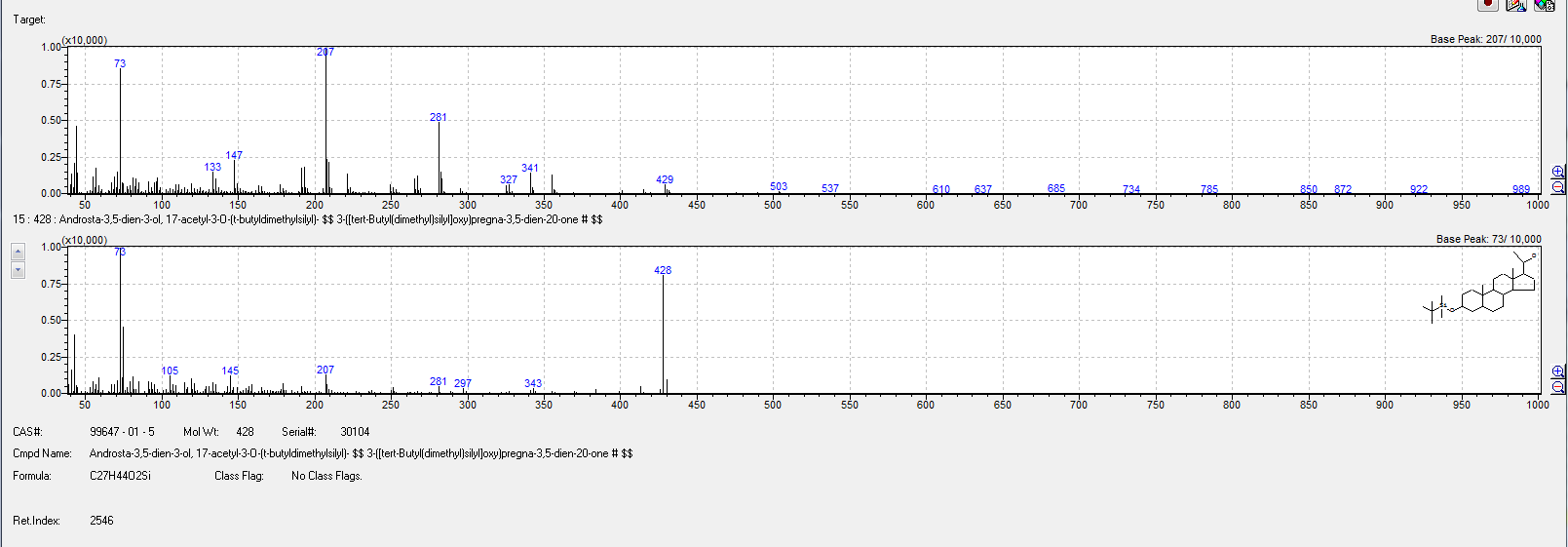 |

**Figure 3.** Fragmentation pattern of compounds identified from the methanol extract of *Sterculia villosa* (MESV).

| Cystine | 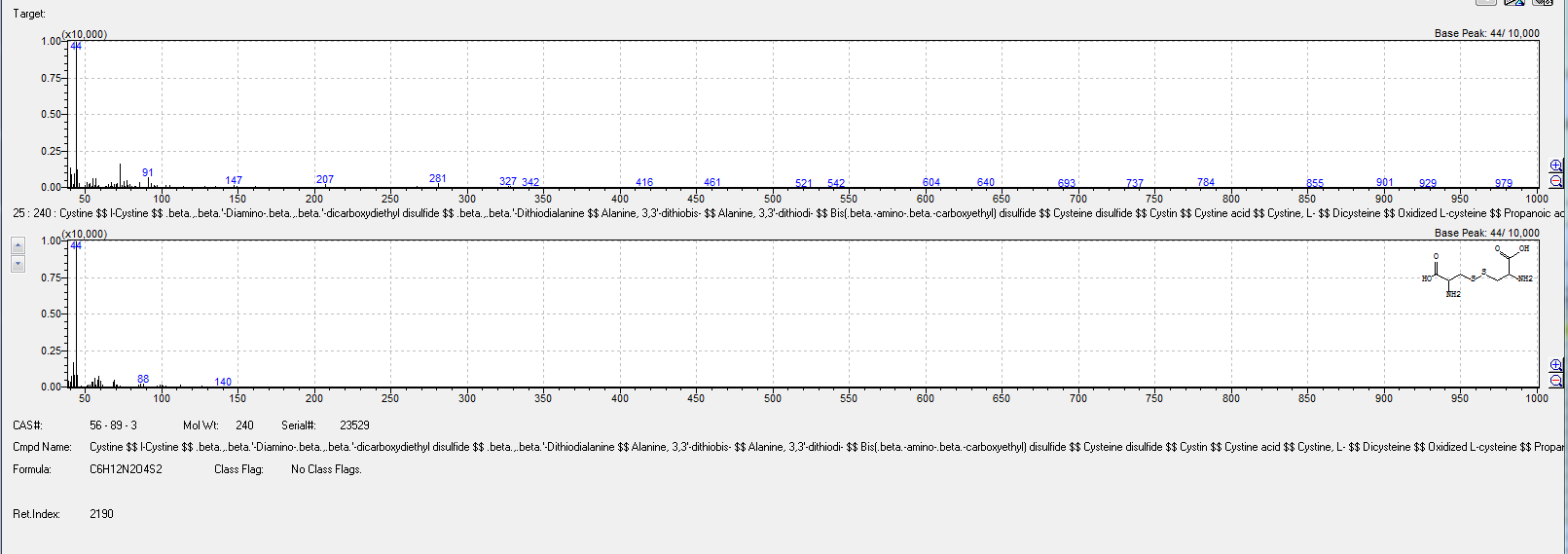 |
| --- | --- |
| D-Alanine | 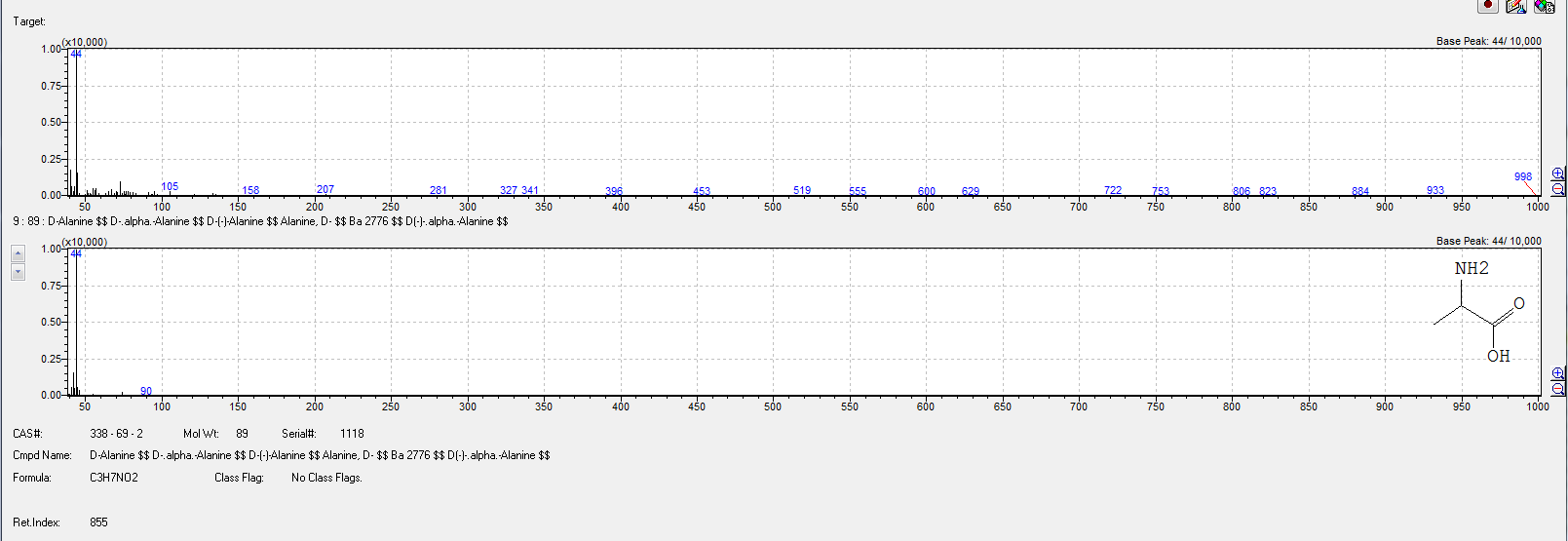 |
| Propanamide | 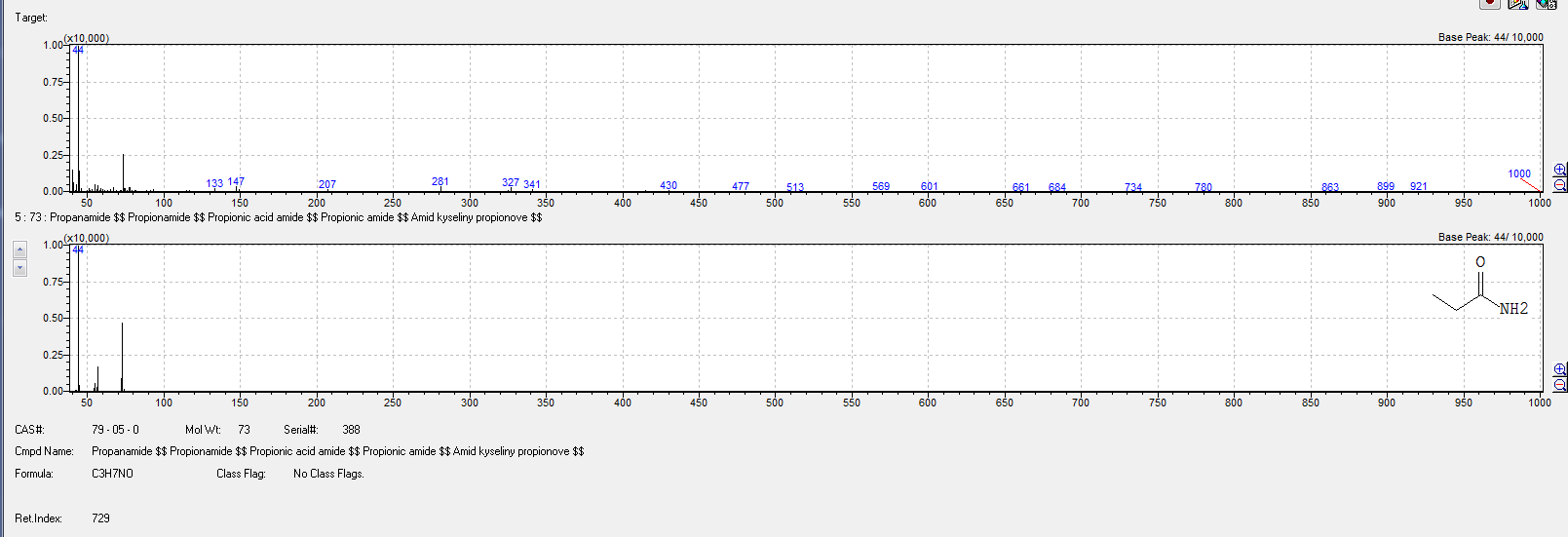 |
| (-)-Norephedrine | 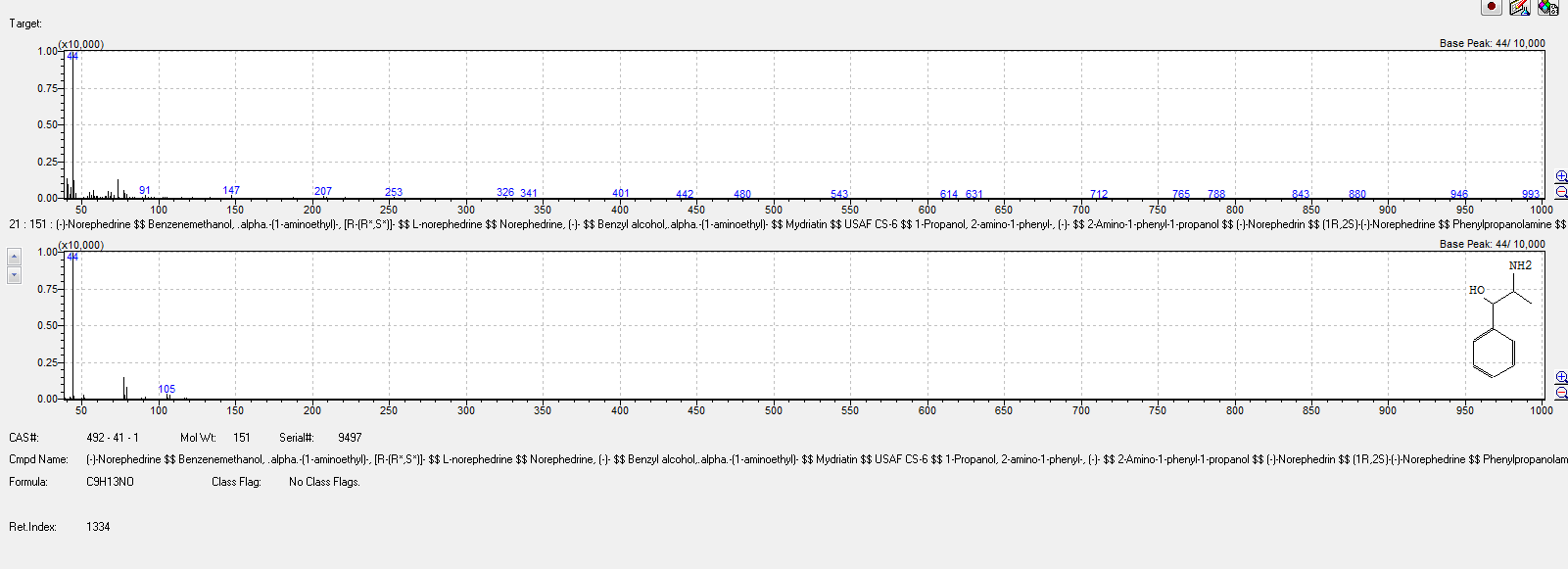 |
| Norpseudoephedrine | 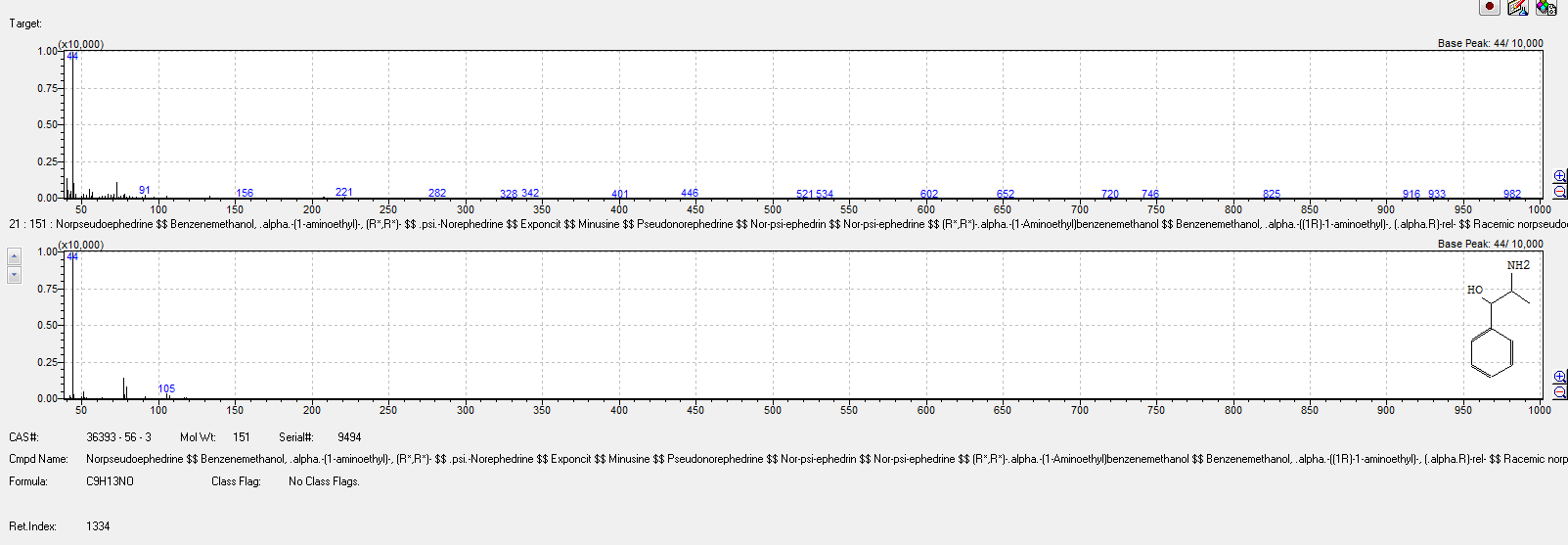 |
| dl-Phenylephrine | 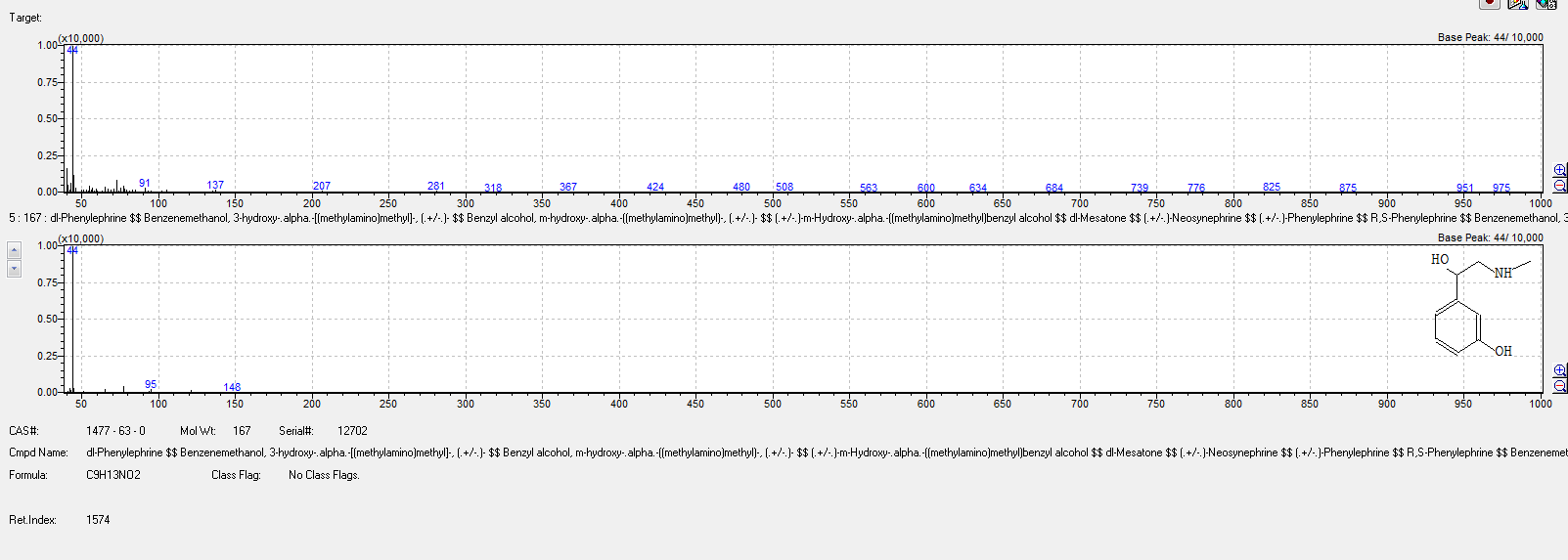 |
| Octodrine | 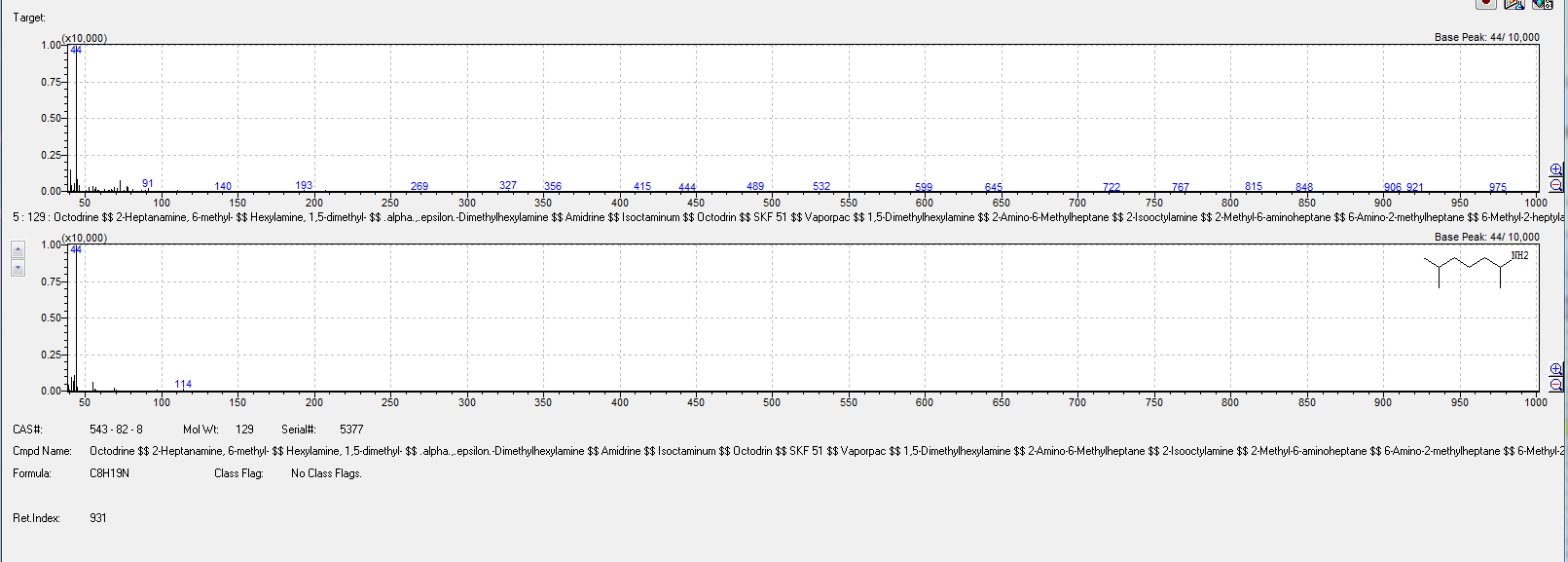 |
| 1,2-Ethanediamine, N-(2-aminoethayl)- | 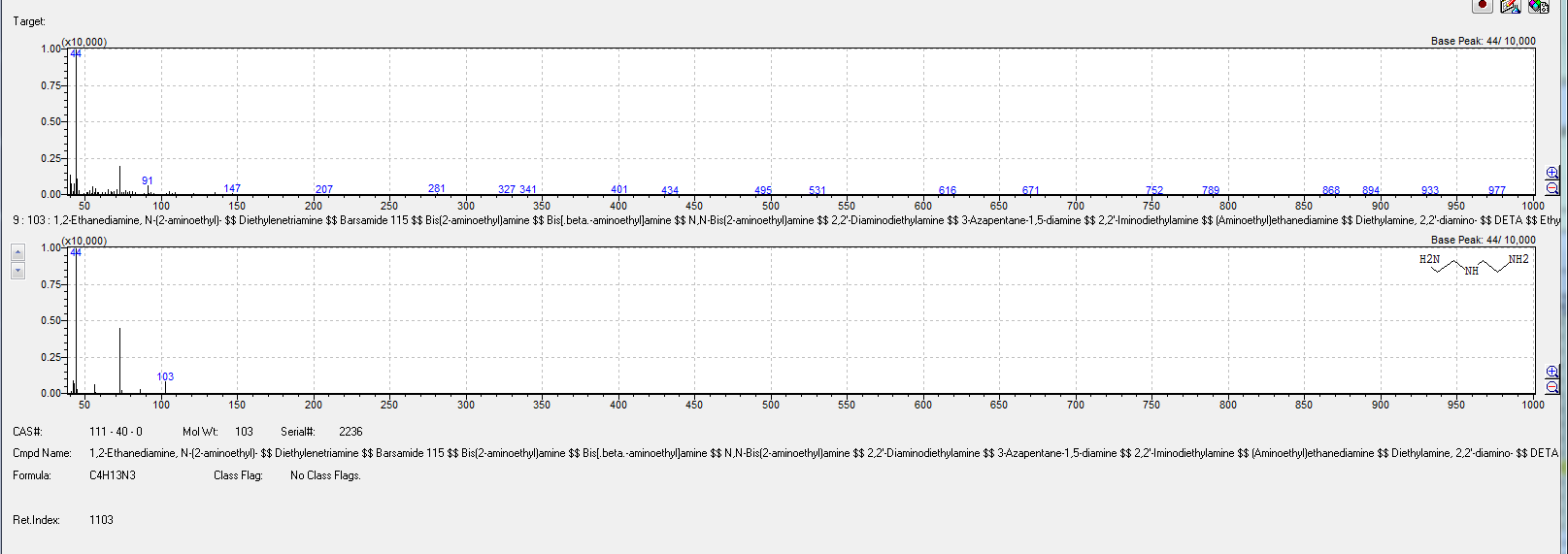 |
| Chlorodifluroacetamide | 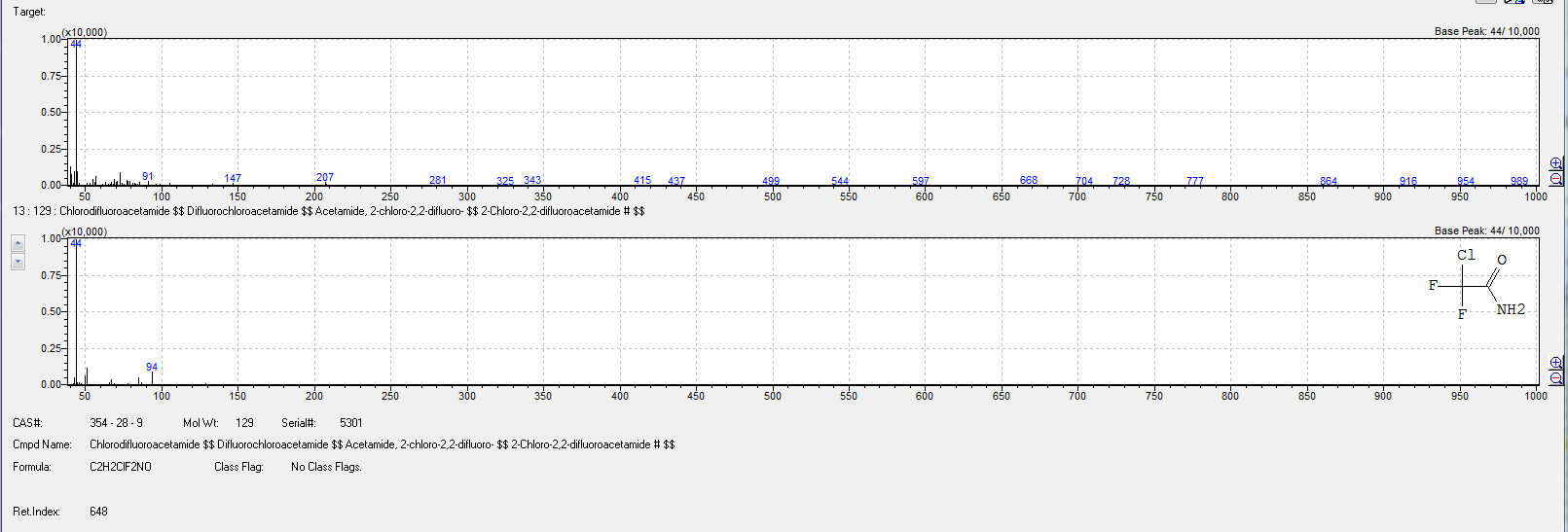 |
| Cathine | 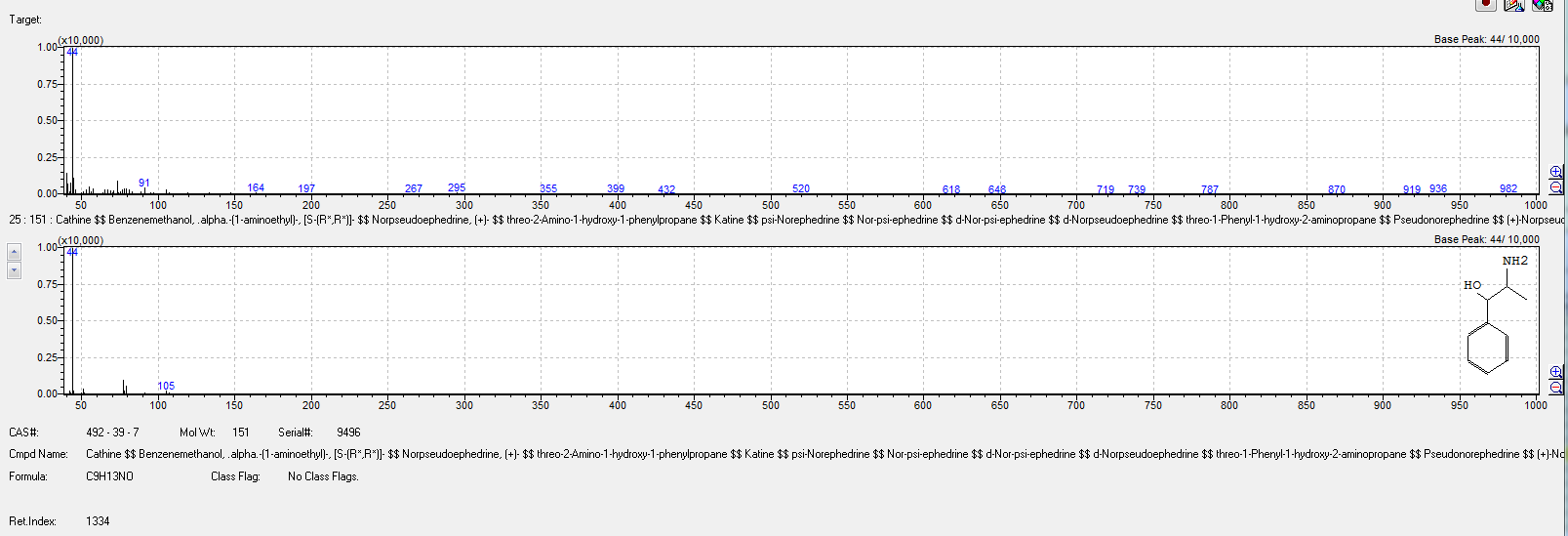 |
| Phloroglucitol | 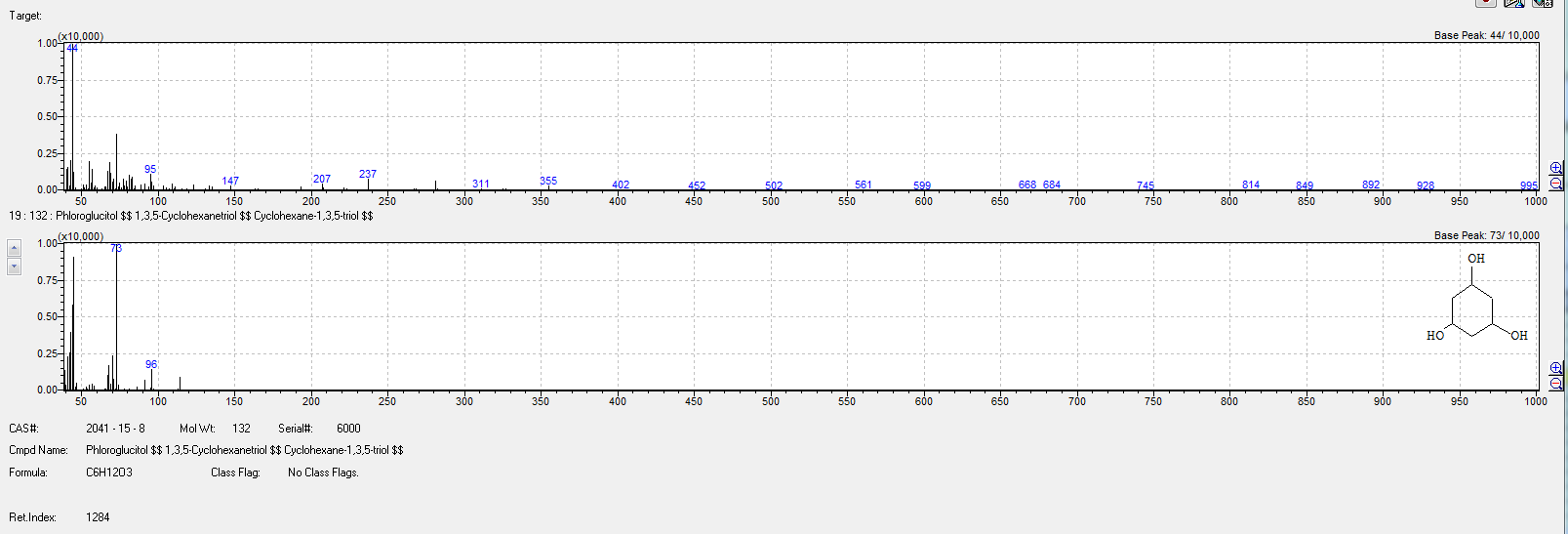 |
| 2-Octinoic acid | 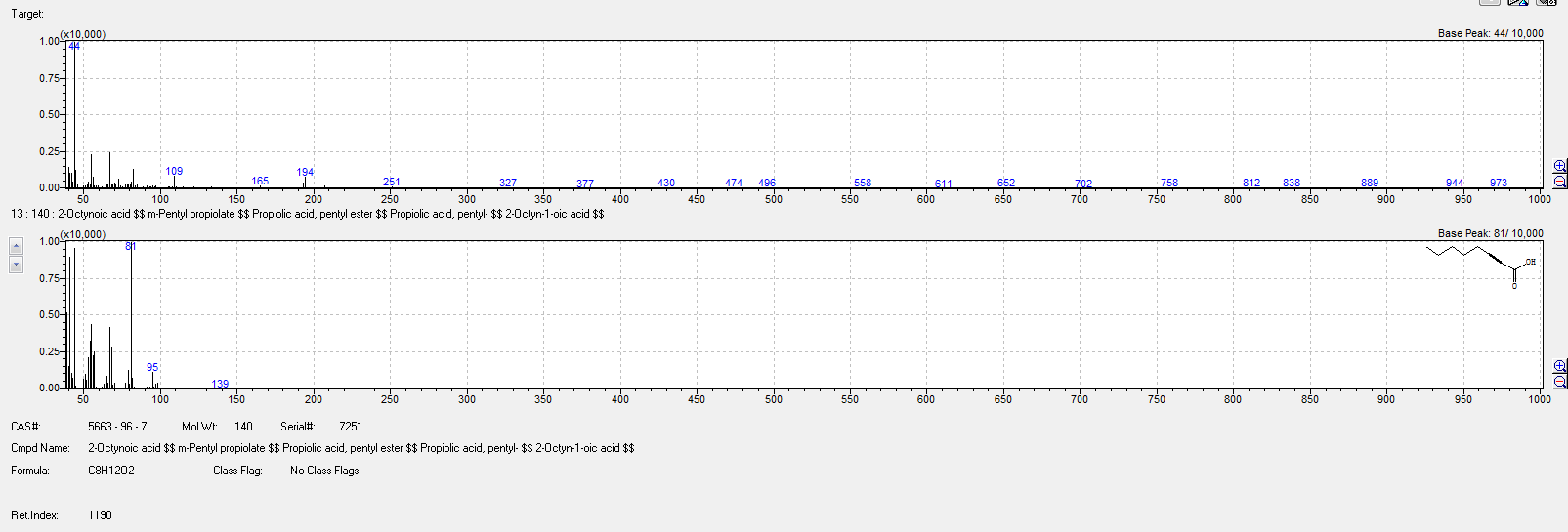 |
| Glutaraldihyde | 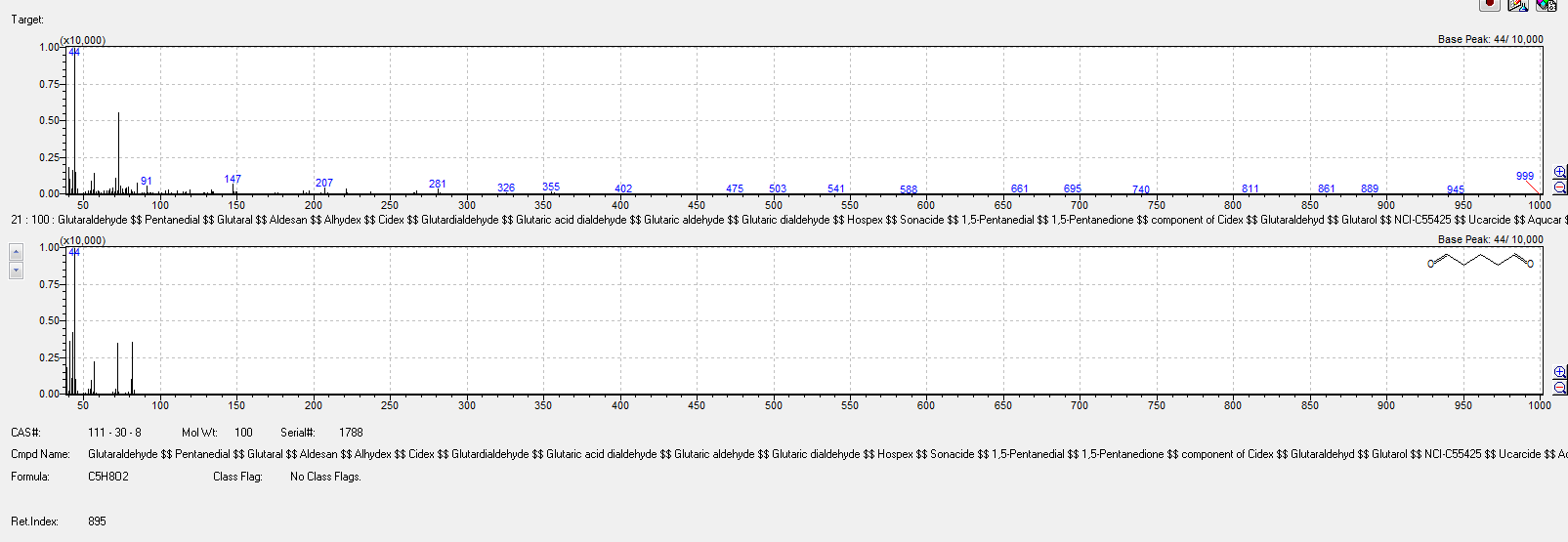 |
| Methyl Stearate | 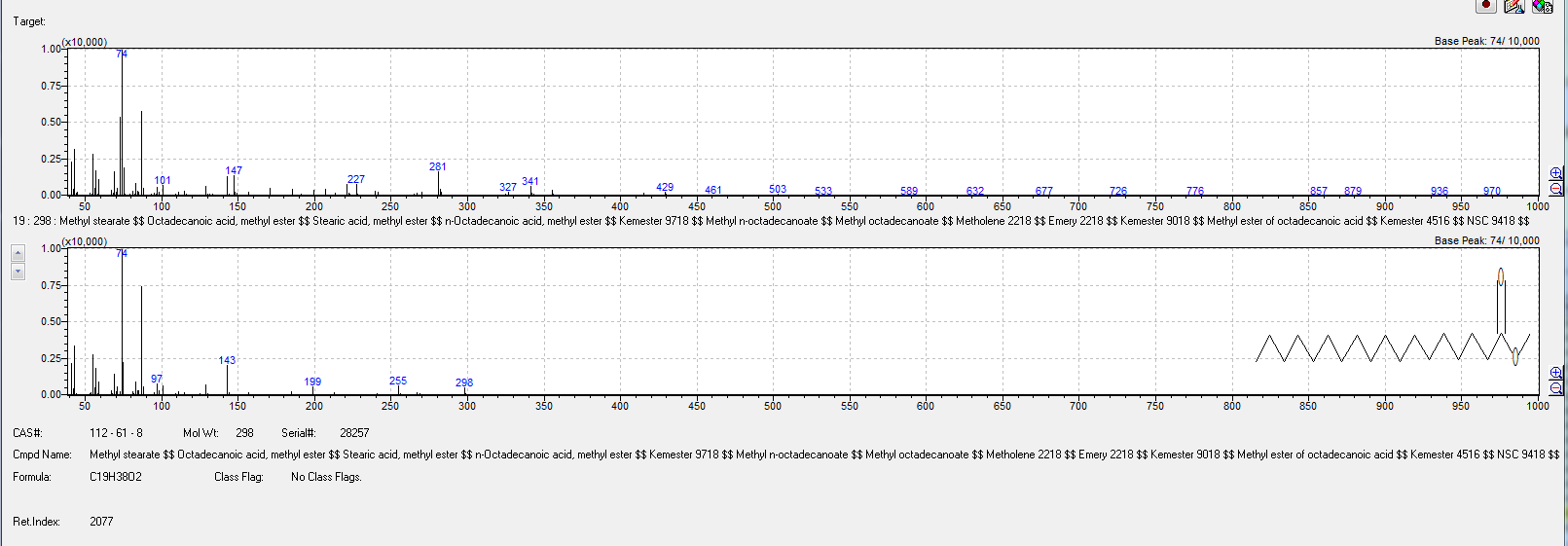 |
| Dibutyl pthalate | 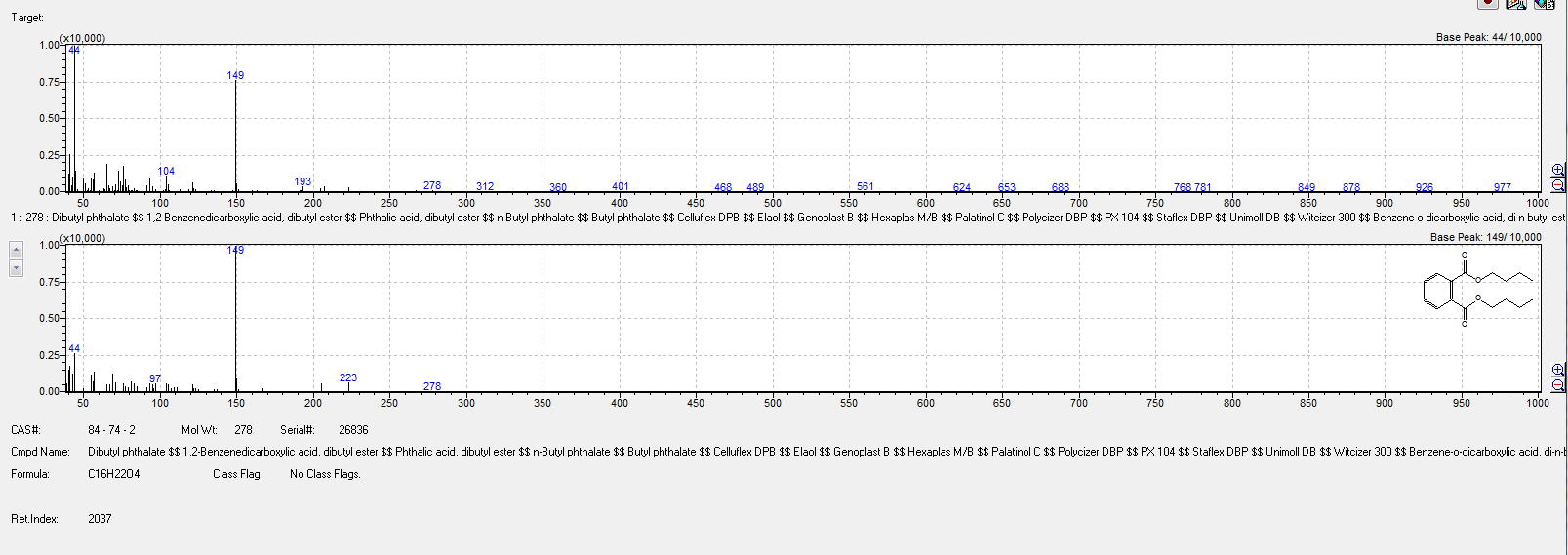 |
| 1-Dodecyne | 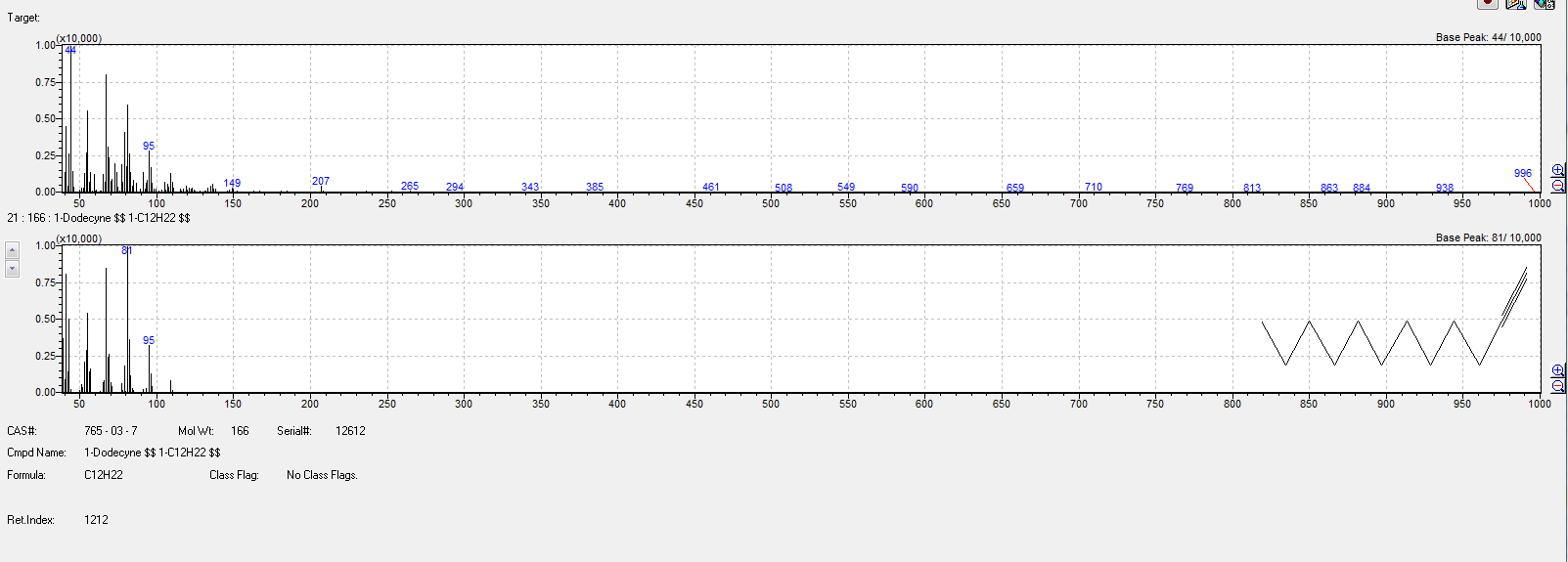 |
| 10-Undecenal | 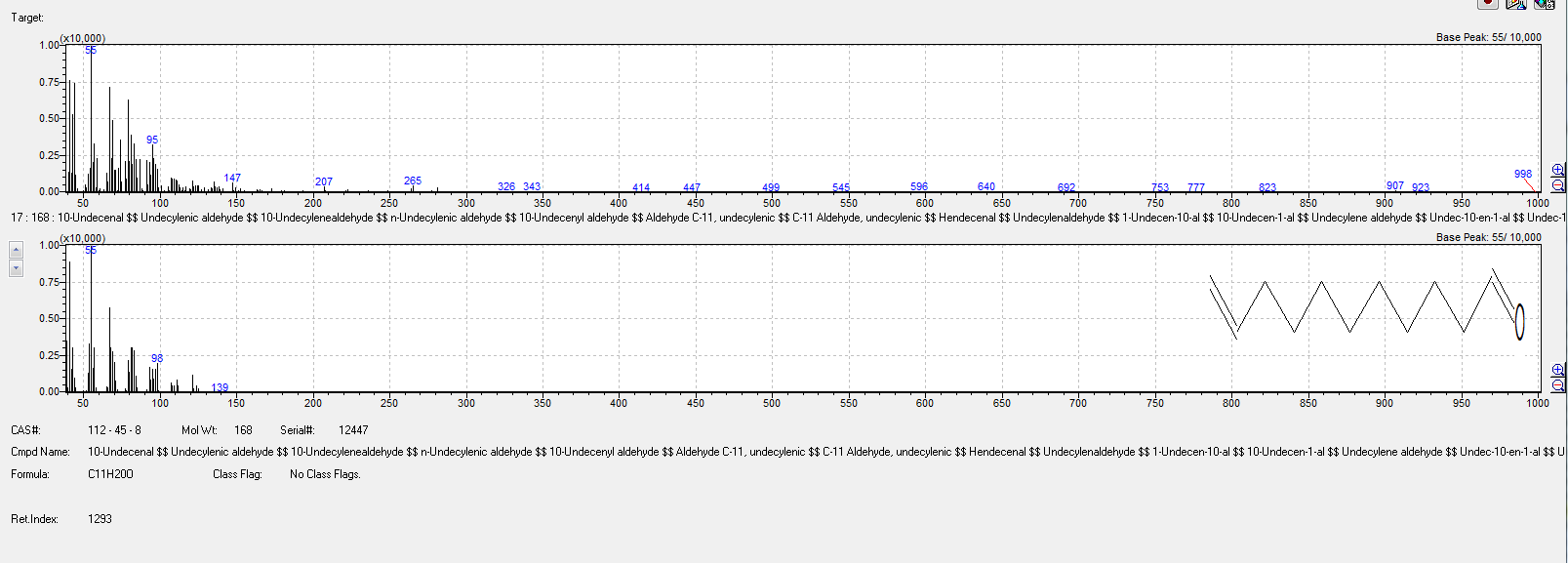 |
| Phytol | 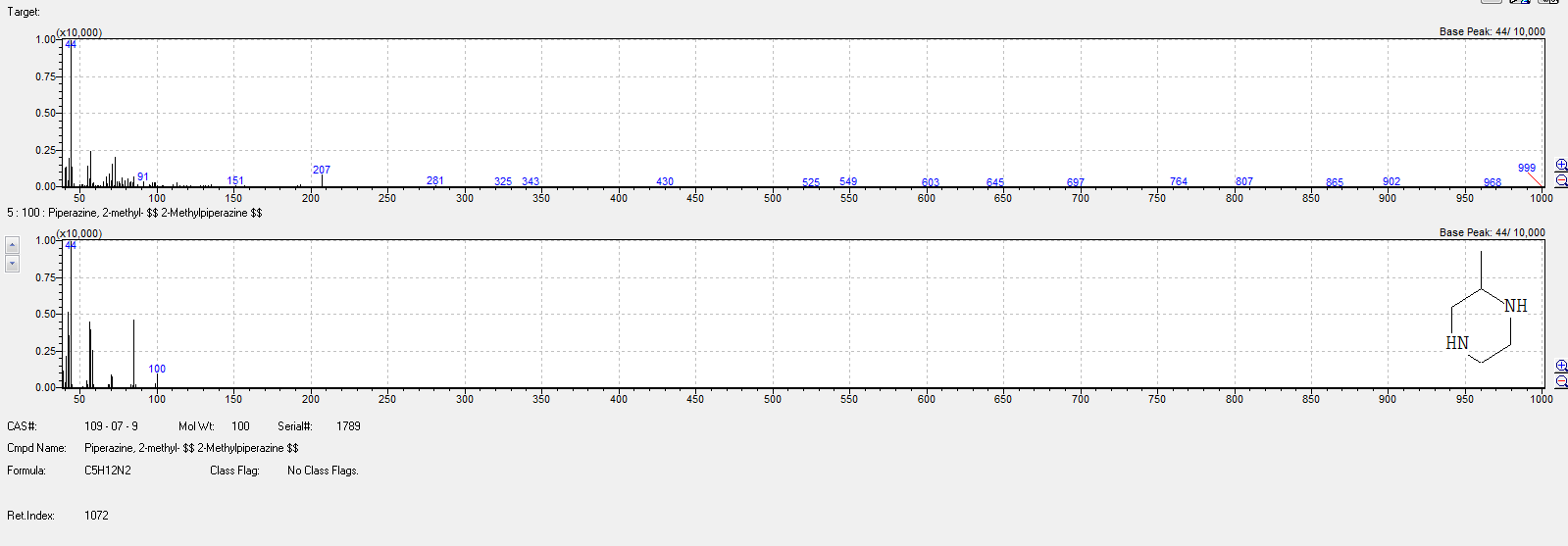 |
| Piperazine | 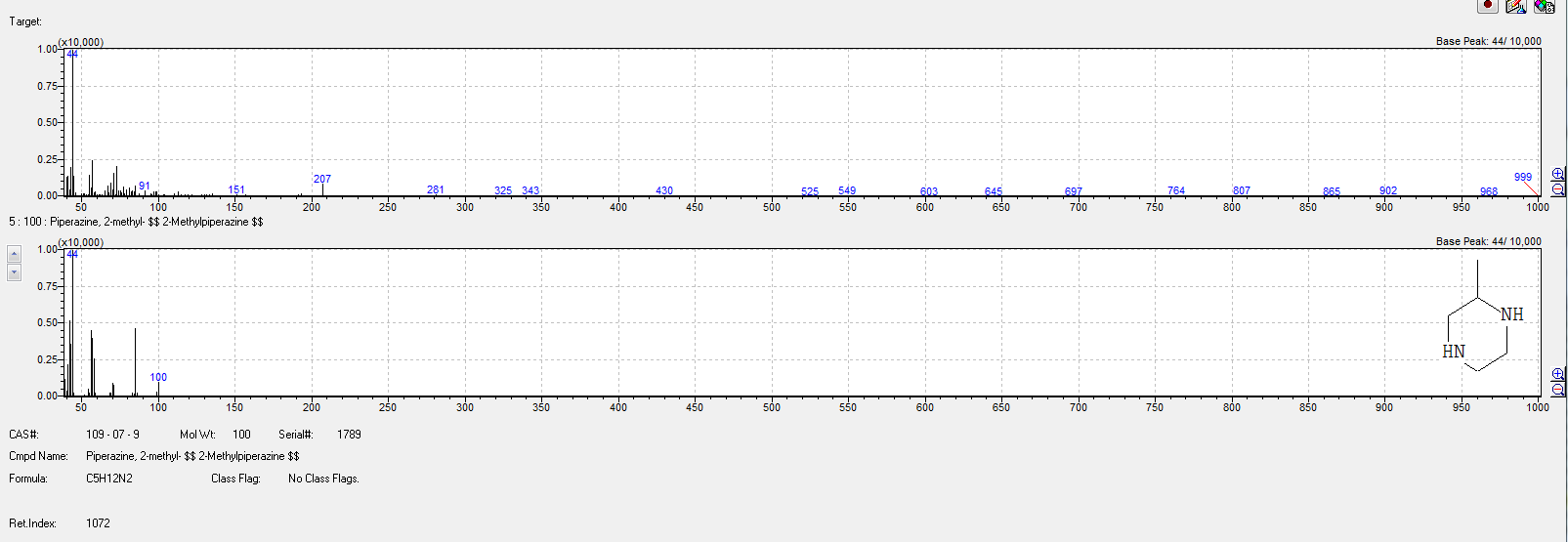 |
| D-Galactonic Acid,gamma-lactone | 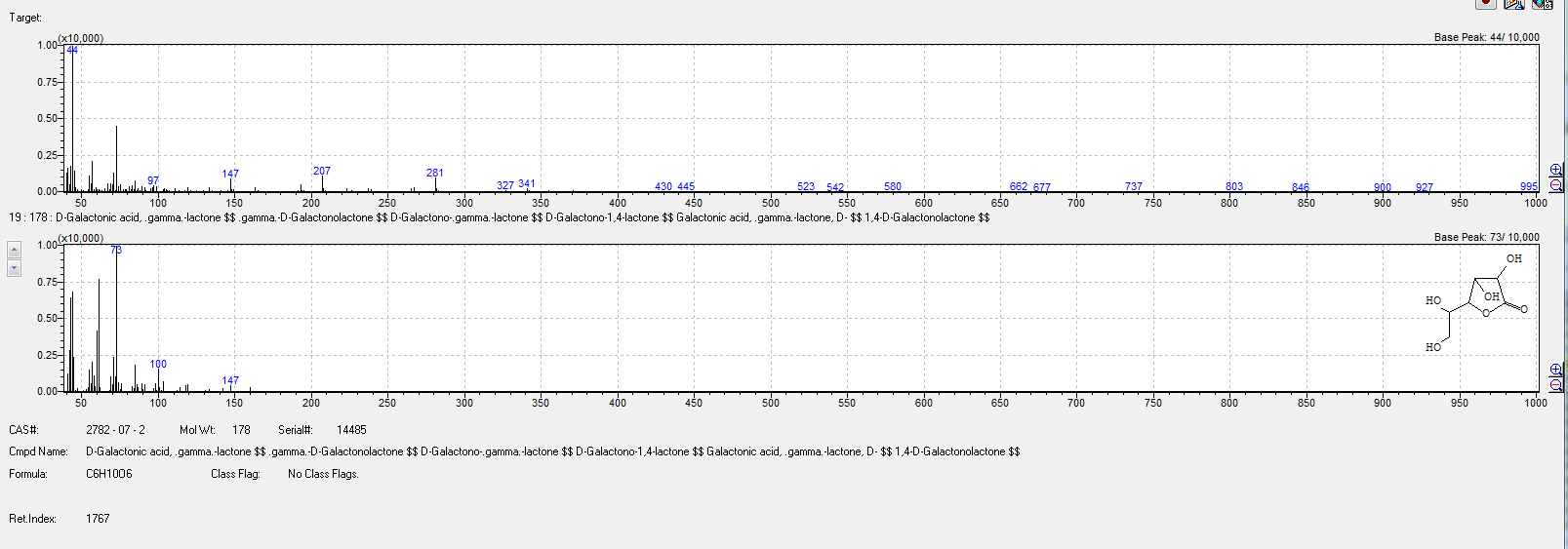 |
| 3,3-Iminobispropylamine | 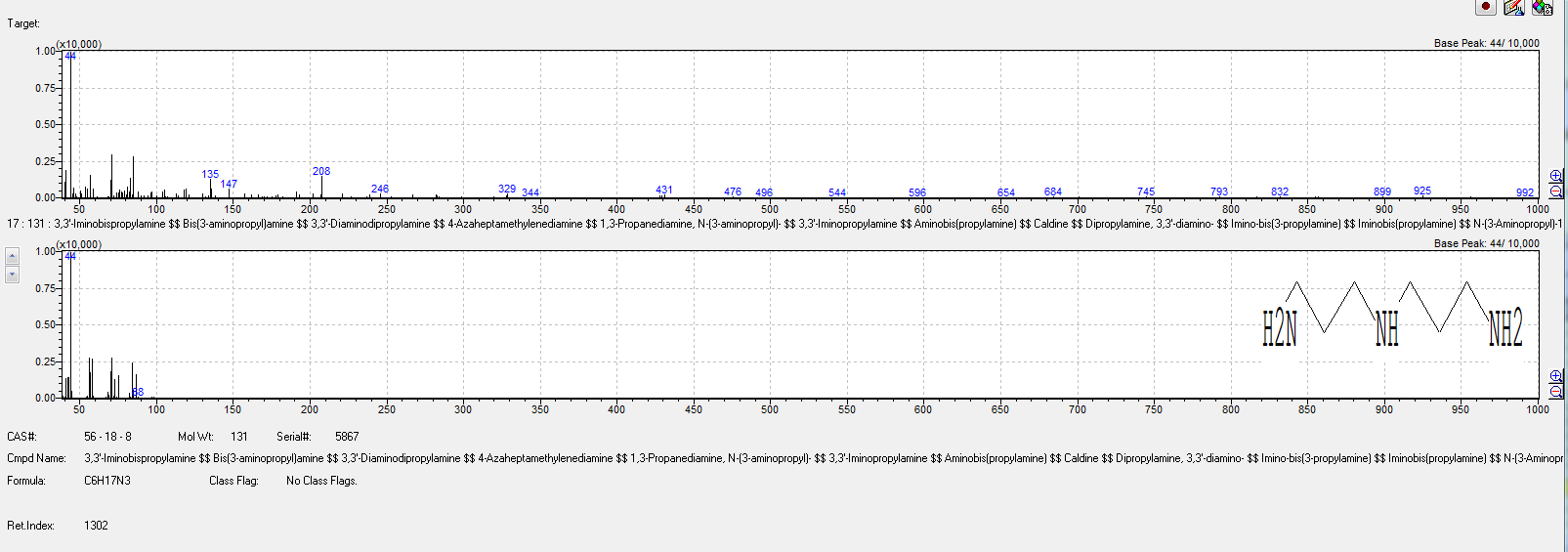 |
| Hexanal | 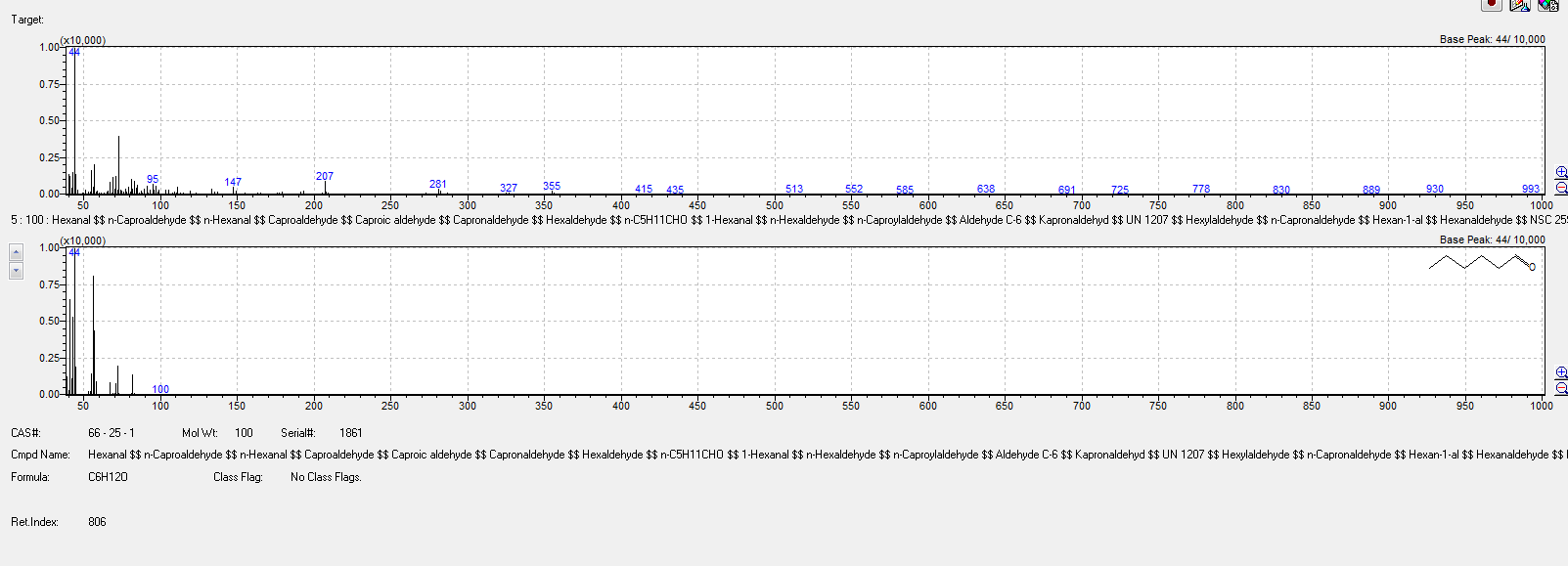 |
| Folic Acid | 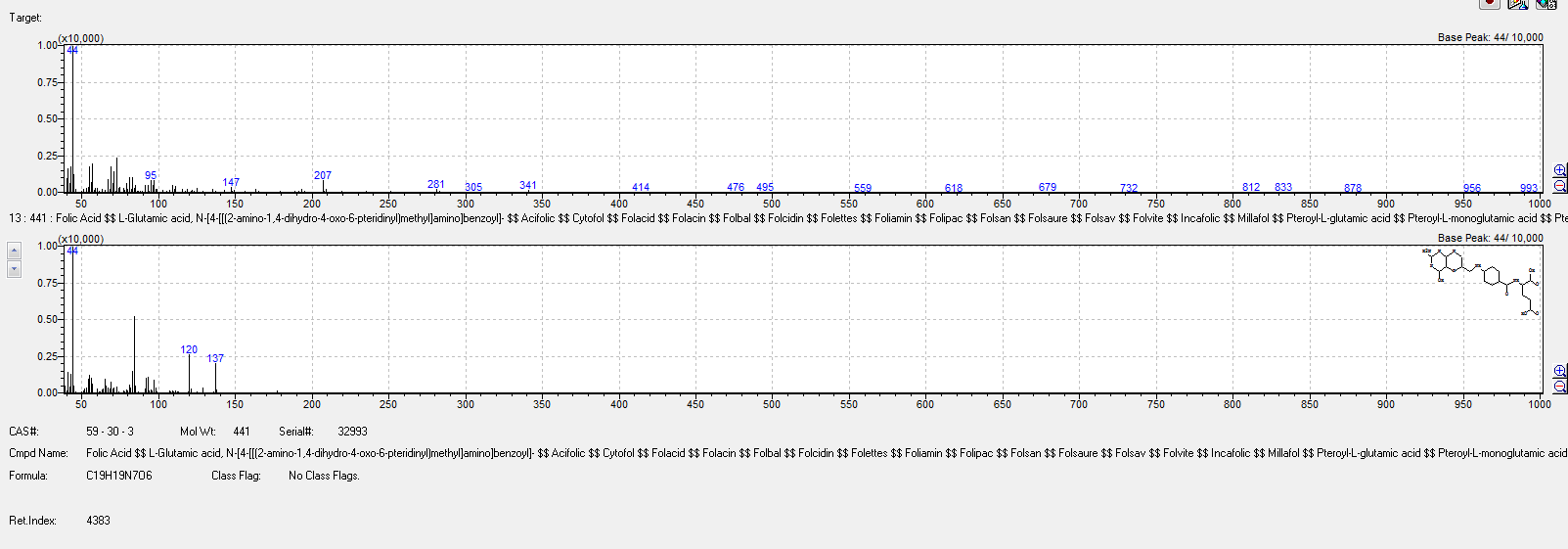 |
| Undecanal | 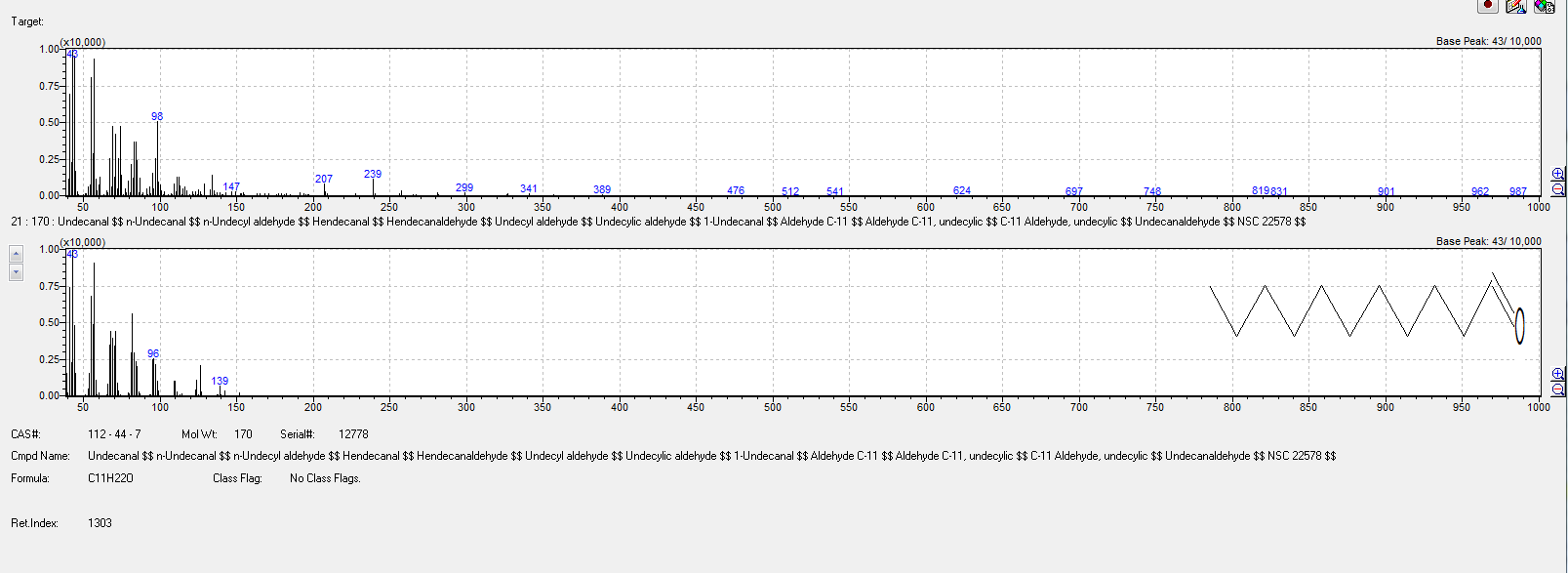 |
| 3,3-Dimethyl piperidine | 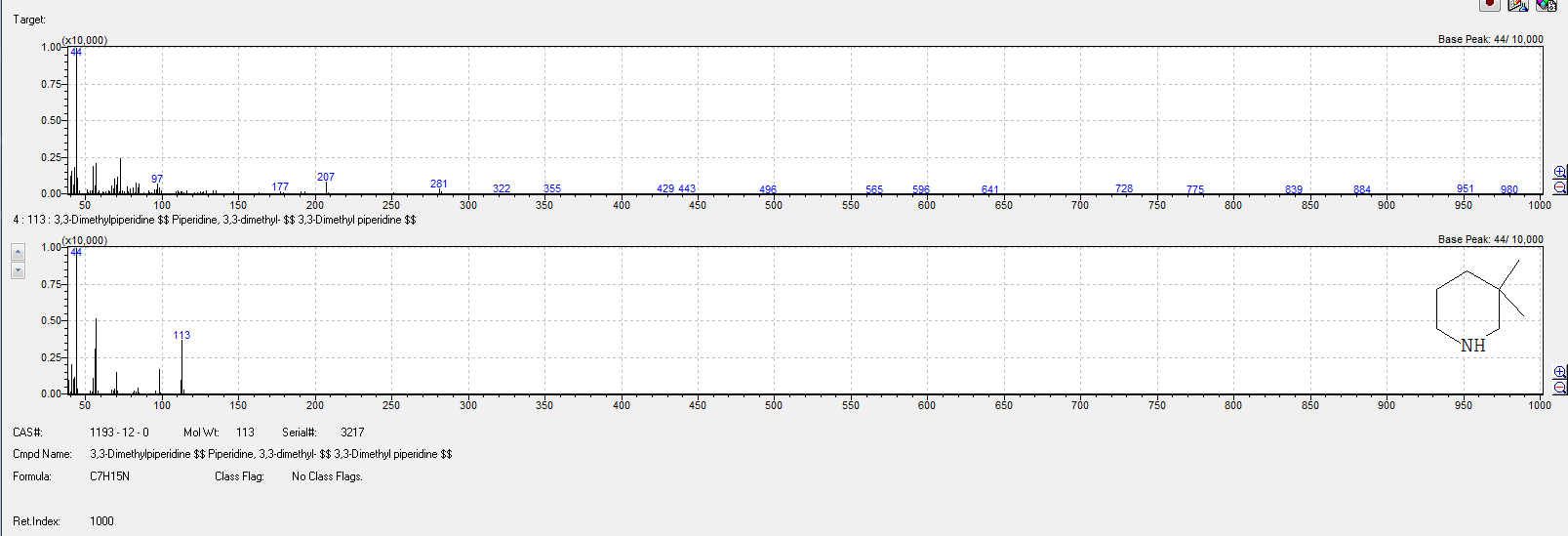 |
| Nonanal | 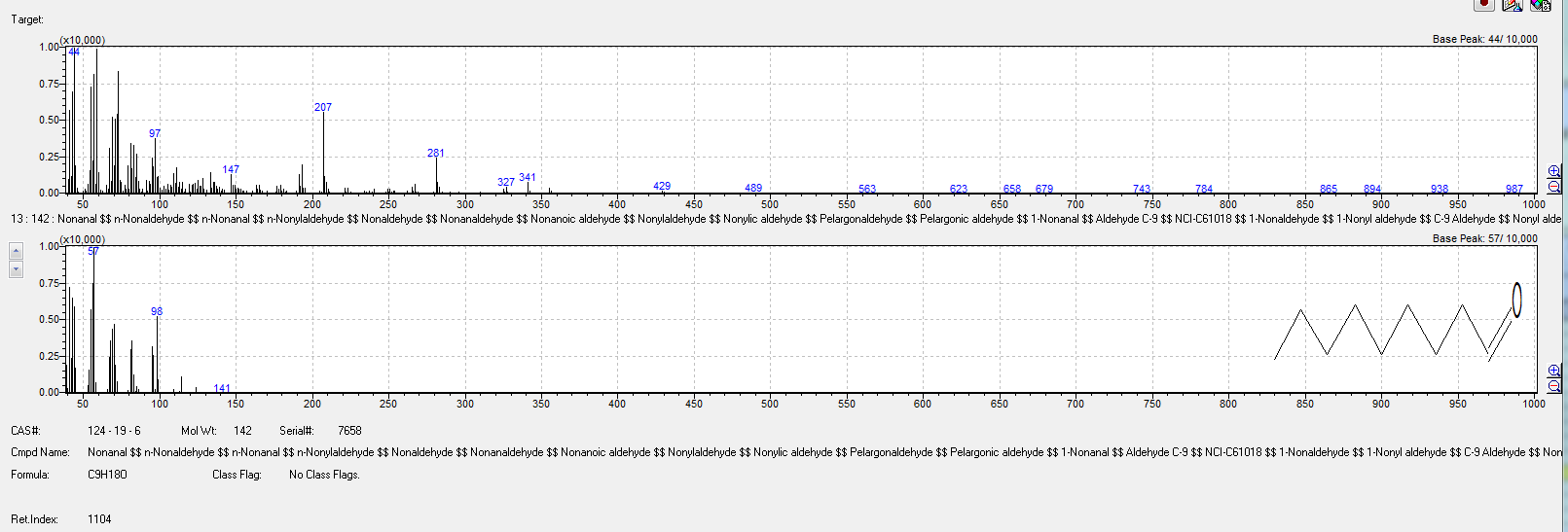 |
| 1-Ecosanol | 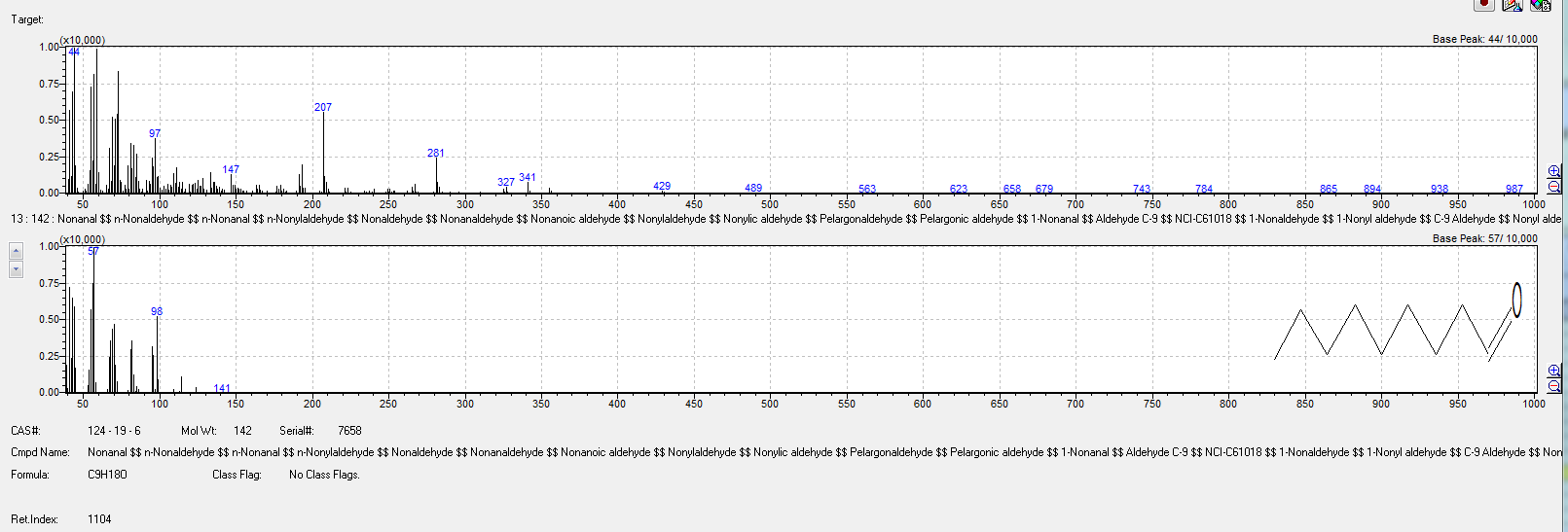 |

**Figure 4.** Fragmentation pattern of compounds identified from the methanol extract of *Vernonia patula* (MEVP).
